# 4-(Indol-3-yl)thiazole-2-amines and 4-ιndol-3-yl)thiazole Acylamines as Νovel Antimicrobial Agents: Synthesis, In Silico and In Vitro Evaluation

**DOI:** 10.3390/ph14111096

**Published:** 2021-10-28

**Authors:** Sergei Simakov, Victor Kartsev, Anthi Petrou, Ioannis Nicolaou, Athina Geronikaki, Marija Ivanov, Marina Kostic, Jasmina Glamočlija, Marina Soković, Despoina Talea, Ioannis S. Vizirianakis

**Affiliations:** 1 Laboratory of General Chemistry, Chemistry Department, Belgorod State University, 308015 Belgorod, Belgorod Oblast, Russia; sergei.smakov@yandex.ru; 2 InterBioScreen, 142432 Chernogolovka, Moscow Region, Russia; vkartsev@ibscreen.chg.ru; 3 School of Pharmacy, Department of Chemistry, Aristotle University of Thessaloniki, 54124 Thessaloniki, Greece; anthi.petrou.thessaloniki1@gmail.com (A.P.); inikolao@pharm.auth.gr (I.N.); 4 Mycological Laboratory, Department of Plant Physiology, Institute for Biological Research “Siniša Stanković”, National Institute of Republic of Serbia, University of Belgrade, 11060 Beograd, Serbia; marija.smiljkovic@ibiss.bg.ac.rs (M.I.); marina.kostic@ibiss.bg.ac (M.K.); jasna@ibiss.bg.ac.rs (J.G.); mris@ibiss.bg.ac.rs (M.S.); 5 Laboratory of Pharmacology, School of Pharmacy, Aristotle University of Thessaloniki, 54124 Thessaloniki, Greece; despoina@bio.auth.gr (D.T.); ivizir@pharm.auth.gr (I.S.V.); 6 Department of Life and Health Sciences, University of Nicosia, Nicosia CY-1700, Cyprus

**Keywords:** 4-(indol-3-yl)thiazole-2-amines, 4-ιndol-3-yl)thiazole acylamines, antimicrobial, antifungal, docking

## Abstract

This manuscript deals with the synthesis and computational and experimental evaluation of the antimicrobial activity of twenty-nine 4-(indol-3-yl)thiazole-2-amines and 4-ιndol-3-yl)thiazole acylamines. An evaluation of antibacterial activity against Gram (+) and Gram (−) bacteria revealed that the MIC of indole derivatives is in the range of 0.06–1.88 mg/mL, while among fourteen methylindole derivatives, only six were active, with an MIC in the range of of 0.47–1.88 mg/mL. *S. aureus* appeared to be the most resistant strain, while *S.* Typhimurium was the most sensitive. Compound **5x** was the most promising, with an MIC in the range of 0.06–0.12 mg/mL, followed by **5d** and **5m**. An evaluation of these three compounds against resistant strains, namely MRSA *P.* *aeruginosa* and *E.* *coli*, revealed that they were more potent against MRSA than ampicillin. Furthermore, compounds **5m** and **5x** were superior inhibitors of biofilm formation, compared to ampicillin and streptomycin, in terms Compounds **5d**, **5m**, and **5x** interact with streptomycin in additive manner. The antifungal activity of some compounds exceeded or was equipotent to those of the reference antifungal agents bifonazole and ketoconazole. The most potent antifungal agent was found to be compound **5g**. Drug likeness scores of compounds was in a range of −0.63 to 0.29, which is moderate to good. According to docking studies, *E. coli* MurB inhibition is probably responsible for the antibacterial activity of compounds, whereas CYP51 inhibition was implicated in antifungal activity. Compounds appeared to be non-toxic, according to the cytotoxicity assessment in MRC-5 cells.

## 1. Introduction

There is a universal current interest in the development of new antimicrobial agents due to the growing emergence of bacterial resistance to antibiotic therapy and to newly emerging pathogens. Antimicrobial resistance (AMR) poses a severe worldwide risk of growing concern to human, animal, and environment health. This is due to the appearance, spread, and persistence of multidrug-resistant (MDR) bacteria, or “superbugs” [1]. AMR is probably due to the unnecessary use of antibiotics in animals (food, pets, marine) and humans, antibiotics sold over the counter, increased global travel, and poor hygiene, especially in developing countries. Another reason could be the release of non-metabolized antibiotics or their residues into the environment through manure/feces. In the absence of the effective treatment, this leads to human losses and the prolonged stay of patients in hospital. Thus, there is an increasing demand for the introduction of new antimicrobial agents due to the developing resistance to conventional antibiotics [2].

**Figure 1:**
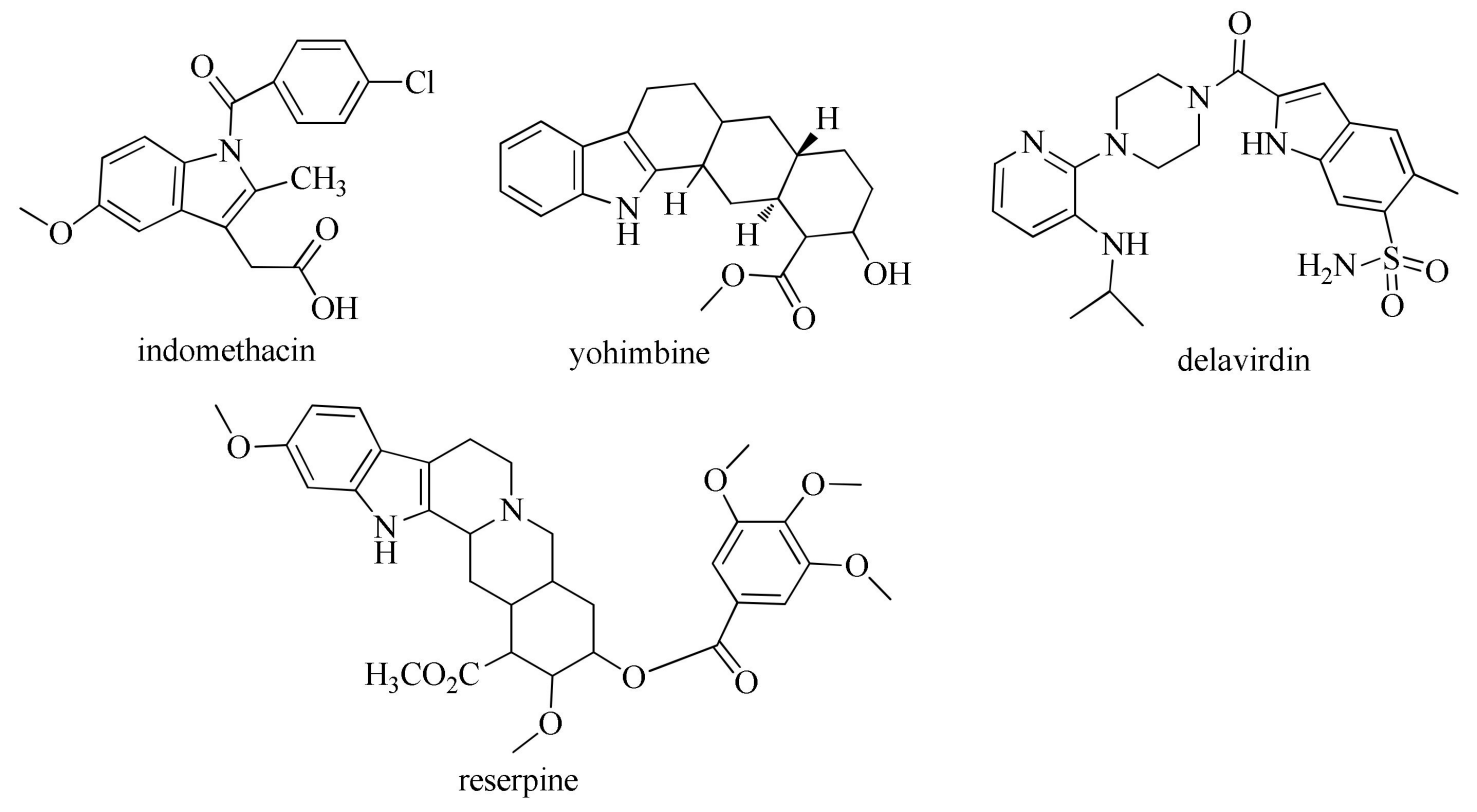
Approved drugs with indole moiety.

Indole, an aromatic heterocyclic organic compound, has attracted the interest of scientists since it possesses a wide variety of pharmacological properties, such as analgesic [3,4], anti-inflammatory [3,5,6], antimicrobial [7,8,9,10], anticancer [11,12,13], anticonvulsant [14,15] and antiviral [16,17], COX-inhibitory [18,19], antidiabetic [20,21], and antitubercular [22,23] activities. Furthermore, indole moiety is present in some drug molecules, such as: indomethacin; yohimbine, an approved drug for the treatment of sexual disorders [24,25]; delavirdine, anti-HIV drug [26]; reserpine (Figure 1), and many others. Indole-based pharmaceuticals represent a very important class of therapeutic molecules and will probably replace many existing pharmaceuticals in the future [27].

**Figure 2:**
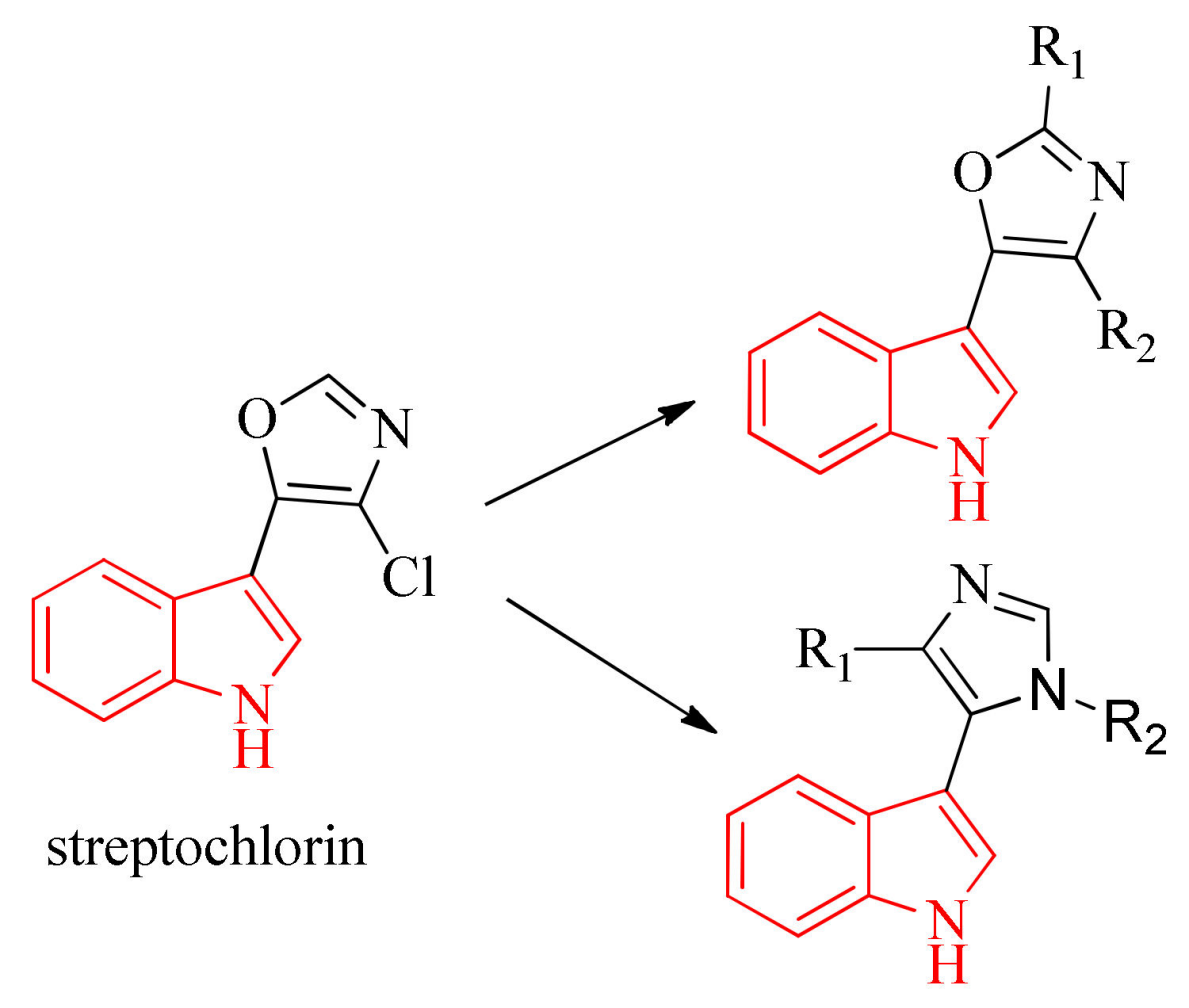
Oxazole and Imidazole analogues of streptochlorin.

On the other hand, it is well known that natural products have served as a powerful source for drug discovery. There have been many important drugs developed from plants and microorganisms [28]. One of them is streptochlorin, a bioactive compound first isolated from marine *Streptomyces* sp. by Japanese scientist H. Watanabe in 1988 [29]. Since streptochlorin lacks antifungal potency in low concentrations, Zhang et al. [30] and Gao et al. [31] made different structural modifications to it and showed the importance of an indole ring in the structure of streptochlorin, replacing the oxazole ring with imidazole as a bioisostere to discover novel streptochlorin analogues (Figure 2).

**Figure 3:**
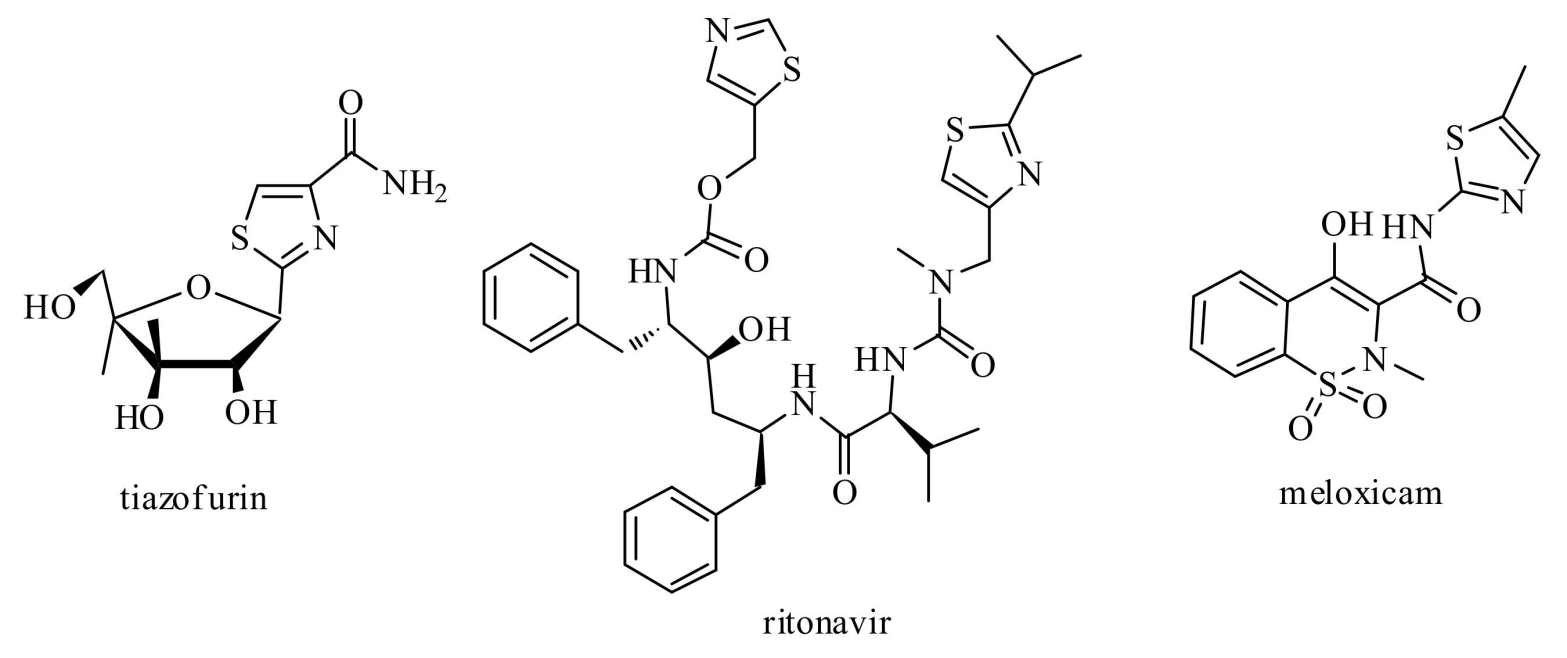
Approved drugs with thiazole moiety.

Another interesting core is thiazole. Its derivatives have been reported to possess a wide range of biological properties, such as antimicrobial [32,33,34], anticancer [35,36,37], anti-inflammatory [38,39,40], antiviral [41,42], anti-HIV [43,44], antidiabetic [45,46], carbonic anhydrase inhibitory [47,48,49], antitubercular [50,51], and many other [52,53,54] activities. Furthermore, penicillin derivatives [55]; bleomycin [56]; tiazofurin [57], an antineoplastic drug; vitamin B1 [58]; ritonavir, an approved drug for the treatment of HIV [59]; and meloxicam [60] (Figure 3) are also very important thiazole-containing agents approved for human use.

One approach in drug design is molecular hybridization based on the combination of two or more pharmacophores of different biologically active molecules in the frame of one single molecule [61]. The aim of this approach is mainly to improve the activity profile and to reduce undesired side effects [62].

Prompted by the factors mentioned above, as well as based on our previous results [63,64], we designed and synthesized new derivatives incorporating two pharmacophores, indole and thiazole moieties, in the frame of one molecule. 

The established human fibroblast-derived MRC-5 normal cell line was chosen to be applied in this work in order to test the cytotoxicity of the new synthesized chemical molecules since its application has been licensed by regulatory bodies for human vaccine production and new drug development [65,66]. To this end, it is reasonable for MRC-5 to be used to develop new antimicrobial and antiviral agents, as well as anticancer therapeutics, in order to evaluate its selectivity in cytotoxicity for bacteria, virus, and malignant cells opposite to normal human cells, as previously published [44,67,68]. 

## 2. Results and Discussion

### 2.1. Chemistry

**Scheme 1:**
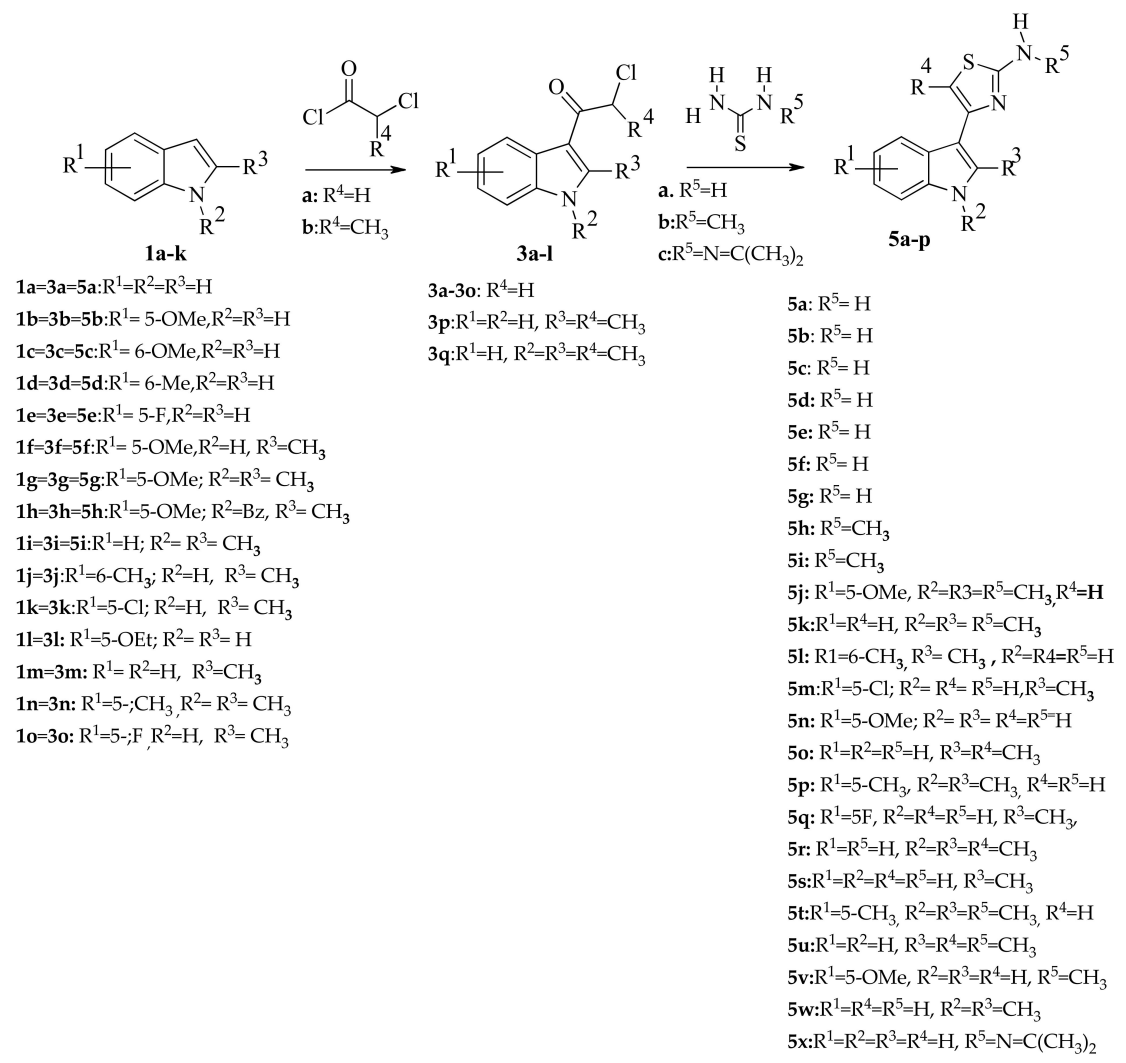
Synthesis of indole-based thiazoles **5a-5x.** Reagents and conditions. (a) pyridine, toluene, 55–60 °C, 1h C; (b) Ki, Na_2_CO_3_, abs. MeOH, reflux.

The title compounds were synthesized according to Scheme 1. Substituted 3-(α-chlorouracil) indoles (**3a–l**) obtained by acylation of the corresponding indoles (**1a–k**) with chloroacetic (**2a**) and α-chloropropionic acid chlorides (**2b**) were used as starting compounds for their synthesis. 3-(α-chlorouracil) indoles (**3a–l**), upon heating in methanol with thiourea (**4a**) and its derivatives (**4b**), in the presence of a base, were converted into the target 4- (indol-3-yl)thiazole-2-amines (**5a–o**) (Scheme 1). Compounds of both groups were obtained good yields in a range of 49–71% for indole-based thiazole derivatives and 49–77% for methylindole thiazole derivatives.

The structure of all of the obtained compounds was confirmed by ^1^H-NMR and ^13^C-NMR spectroscopy. In the ^1^H-NMR spectra of 3- (α-chlorouracil) indoles (**3a–l**), the signals of the protons of the –CO–CH_2_–Cl group are in the range of 4.5–4.7 ppm, while the signals of the NH protons of the indole ring were observed at 11.59–12.01 ppm. In the ^1^H-NMR spectra of 4- (indol-3-yl)thiazole-2-amines (**5a-n, 5p, 5q, 5s**) obtained on the basis of 3- (α-chloroacetyl) indoles (**3a–o**), there is a proton singlet in position 5 of the thiazole fragment in the range of 6.2–6.5 ppm. In the ^1^H-NMR spectra of compounds (**5o, 5r, 5u**) synthesized using 3- (α-chloropropionyl) indoles (**3p–3q**), the signal of the protons of the methyl group is in the range of 2.12–2.22 ppm. The signal of the protons of the NH_2_ group in 4-(indol-3-yl) thiazol-2-amines (**5a–5f, 5h–m**), formed upon condensation of 3-(α-chlorouracil) indoles with unsubstituted thiourea (**4a**), appears in the region of 6.6–6.8 ppm, while in the spectra of compounds (**5g, 5n, 5o**) obtained using N-methylthiourea (**4b**), the signal of the protons of the N-methyl group is in the range of 2.8–2.9 ppm. In the spectrum of compound **5p,** the protons of the methyl groups of the isopropenyl fragment are represented by a doublet at 2.12 ppm.

**Scheme 2:**
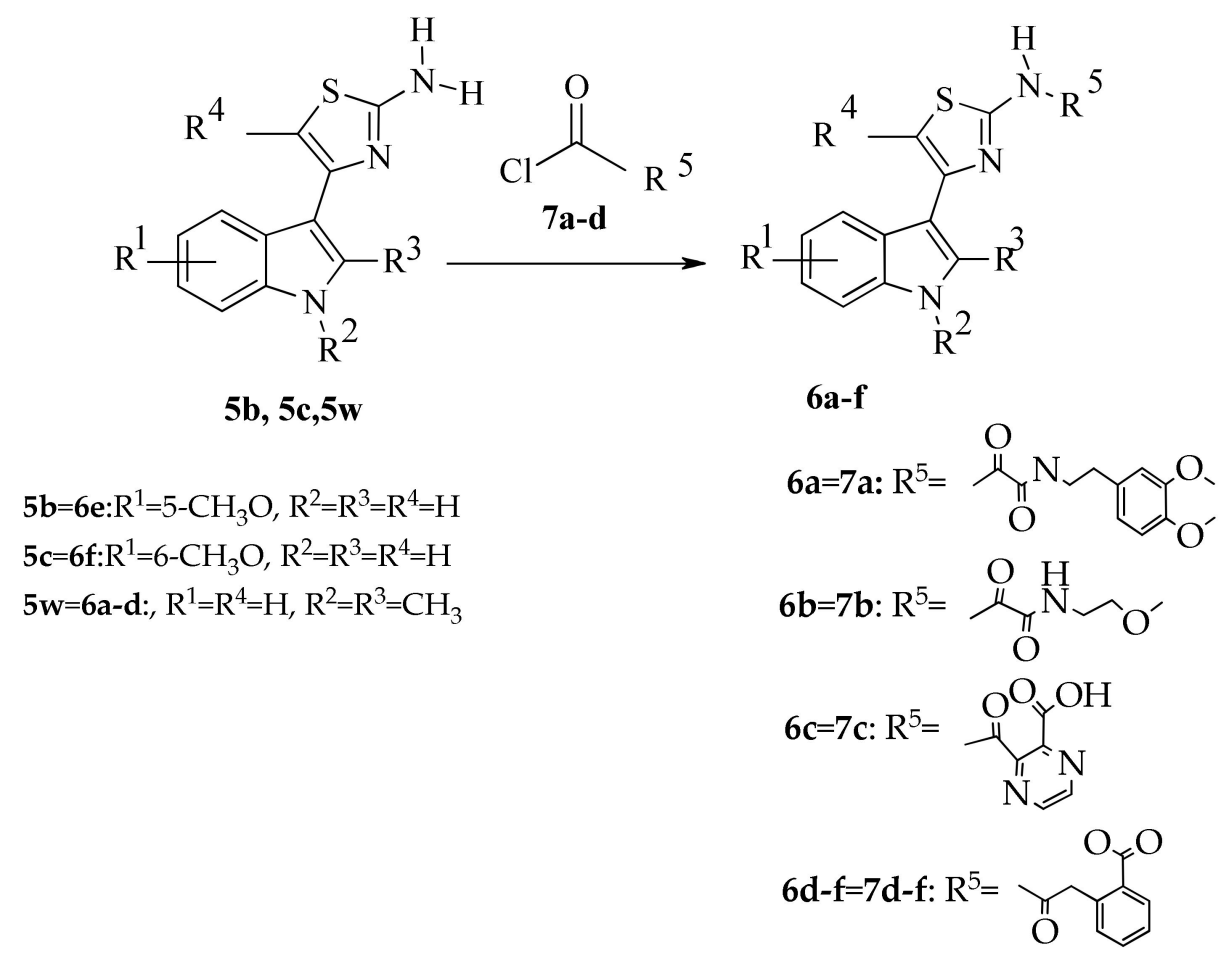
Synthesis of indole-based thiazoles **6a-f** by acylation of aminothiazoles **5b, 5c, 5w**. Reagents and conditions: pyridine, **5h**, stiurring stiurring.

For the synthesis of 4-(indol-3-yl)thiazol-2-amines (**6a–f**) containing an acyl residue in the amino group, 4- (indol-3-yl) thiazol-2-amines (**5b, 5c, 5w**) were treated with acid chlorides of the corresponding acids (**7a–d**) in pyridine (Scheme 2).

In the ^13^C-NMR spectra, the signal of the O–CH_3_ group was observed in the range of 55.84–55.97 ppm, with that of the methyl group in the range of 14.22–14.78 ppm. C–NH_2_ signals appeared at 167.11–167.34ppm, while C=O appeared at 158.27–170.02 ppm. The signals of C–OCH_3_ group are represented at 154.02–154.21 ppm, while those of COOH are represented at 172.12 ppm. Finally, the signals of the C–NHCH_3_ group were observed at 163.29 ppm.

In the ^1^H-NMR spectra of 4- (indol-3-yl) thiazol-2-amines (**6a–f**) formed as a result of acylation of compounds (**5b, 5c, 5w**), the signals of the acyl group protons were observed.

### 2.2. Biological Evaluation

All compounds (**29**) were evaluated for their possible antibacterial activity using the microdilution method and were divided in two subgroups. The first group consists of indole-based thiazole derivatives (**5a–f, 5i**, **5l–o, 5q, 5s, 5u, 5v,5x**), and the second group is methylindole-based thiazole derivatives (**5g-5k, 5p,5r, 5t, 5w, 6a–6f**). Ampicillin and streptomycin were used as reference drugs. All compounds of the first subgroup of indole-based thiazoles showed antibacterial activity, with MIC in the range of 0.06–1.88 mg/mL and MBC of 0.12–3.75 mg/mL. The antibacterial potency of the tested compounds is shown in Table 1, and activity order can be presented as follows: **5x > 5m > 5d > 5e > 5a > 5q > 5s > 5b > 5v > 5f > 5l > 5c = 5n = 5o > 5i > 5u**. Thus, the best activity was achieved for compound **5x**, with MIC and MBC of 0.06–0.12 mg/mL and 0.12–0.23 mg/mL, respectively, whereas compound **5u** showed the lowest activity, with MIC/MBC in the range of 0.47–3.75/0.94–3.75 mg/mL.

The most sensitive bacterium was found to be *S.* Typhimurium (ATCC 13311), with the lowest MIC of 0.06 mg/mL (**5x**) and 0.12 mg/mL (**5a**) and the highest at 1.88 mg/mL (**5o** and **5u**). *S. aureus* (ATCC 6538) was the most resistant strain, with the lowest MIC of 0.12 mg/mL (**5m** and **5x**), and the highest at 3.75 mg/mL (**5i**). In general, all strains were moderately sensitive to the compounds tested.

Compound **5e** showed promising activity against *B. cereus* and *L. monocytogenes*, with MIC/MBC of 0.12/0.23 mg/mL. Nevertheless, none of compounds exceeded the activity of the reference drugs.

Compound **5x** exhibited the highest activity among the tested compounds against *S.* Typhimurium (ATCC 13311), while compound **5m** exhibited the highest activity against *B. cereus* and the most resistant bacterium, *S. aureus*, (ATCC 6538) with MIC of 0.06 mg/mL and MBC of 0.12 mg/mL, exceeding the activity of ampicillin. Good activity against *S.* Typhimurium (ATCC 13311) was observed for compound **5a**, whereas compound **5e** showed good activity against *B. cereus* and *L. monocytogenes*, with MIC/MBC of 0.12/0.23 mg/mL. Nevertheless, none of other compounds exceeded the activity of the reference drugs.

According to structure-activity relationships, the presence of propan-2-ylidenhydrazine substituent at position 2 of the thiazole ring (**5x**) appeared to be most beneficial for antibacterial activity. The introduction of an Me group at position 2 and a 5-Cl substituent to the indole ring, as well as the replacement of propan-2-ylidenhydrazine by an amino group (**5m**) slightly decreased the activity. The presence of an amino group in position 2 of thiazole, as well as a 6-Me-group in the indole ring led to compound, **5d** less active than previous. The replacement of the 5-Cl of compound **5m** by a 5-OMe group and the introduction a methylamino group in position 2 of the thiazole ring (**5i**) appeared to be detrimental to antibacterial activity. The presence of 2-methylamino, as well as a methyl group, in position 5 of the thiazole ring (**5u**) had the most negative effect. It should be mentioned that derivatives with a 2-NH_2_ group in the thiazole ring, independent of substituents in the indole ring (**5a, 5d, 5e, 5m, 5q** and **5s**), were among the most potent.

Thus, it can be concluded that antibacterial activity depends not only on substituents and their position in the indole ring but also on substituents in position 2 of the thiazole moiety.

The three most active compounds (**5x, 5m** and **5d**) were also studied for their activity against resistant strains, including methicillin-resistant *S. aureus, P. aeruginosa*, and *E. coli*. From the results, presented in Table 2, it is obvious that all compounds appeared to be more potent against *MRSA* than ampicillin, whereas streptomycin did not exhibit bactericidal activity. As far as the other two resistant strains are concerned, these compounds were less active than both reference compounds, even though ampicillin did not show bactericidal activity. 

The compounds were evaluated then for their ability to stop biofilm formation. The obtained results are promising. Both compounds (**5m** and **5x**) showed stronger inhibition of biofilm formation than both reference compounds in terms of concentration of MIC. Compound **5x** was active at lower concentration compared to **5m** (0.47 mg/mL and 0.84 mg/mL, respectively). However, it should also be mentioned that the second, in order of activity, compound **5m**, was more potent against biofilm formation than both reference drugs, even at a concentration of 0.5 MIC, while the ability of compound **5d** was less not only than that of both reference drugs but also than that of the other two compounds. Both compounds **5m** and **5x** displayed strong antimicrobial potential, represented by both low MICs towards non-resistant (Table 1) and resistant strains (Table 3) and by strong antibiofilm potential towards *P. aeruginosa*. Since the majority of infections are associated with biofilm-forming microorganisms, these compounds have promising potential for the development of novel antibiofilm therapeutics since they can reduce growth of both planktonic and biofilm-associated microbial cells.

As far as the second subgroup of compounds is concerned (methylindols), they did not show remarkable antibacterial activity (Table S1, Antibacterial activity of methylindole derivatives. (MIC and MBC in mg/mL, Supplementary Files)). More than half of the compounds were of very low activity (MIC/MBC > 3.75 mg/mL), and only compounds **5g, 5h, 5i, 5j, 5k**, and **5w** showed moderate activity, with MIC of 0.47–1.88 mg/mL and MBC of 0.94–3.75 mg/mL against bacteria tested, except *S. aureus.* As in case of indole derivatives, *S. aureus* was the most resistant bacteria, followed by *L.monocytogenes*, while *B. cereus* was the most sensitive strain. 

According to structure-activity relationships, the presence of 2-Me, 6-OMe substitution in the methylindole ring and 2-NH_2_ substitution in the thiazole ring (**5g**) appeared to be the most beneficial. 

### 2.3. Additive Effect of Selected Indole Derivatives in Combination with Streptomycin

The three selected compounds were determined for the interactions with antibiotic streptomycin using checkboard assay. All of the examined compounds were additives with streptomycin (FICI 1.5, Table 2), suggesting, based on the in vitro data, that the combination of compounds with this antibiotic can reduce its MIC and subsequently increase its efficiency.

### 2.4. P. aeruginosa Time-Kill Curve Assay Efficient of P. aeruginosa Bactericidal Effect after 1 h 

**Figure 4:**
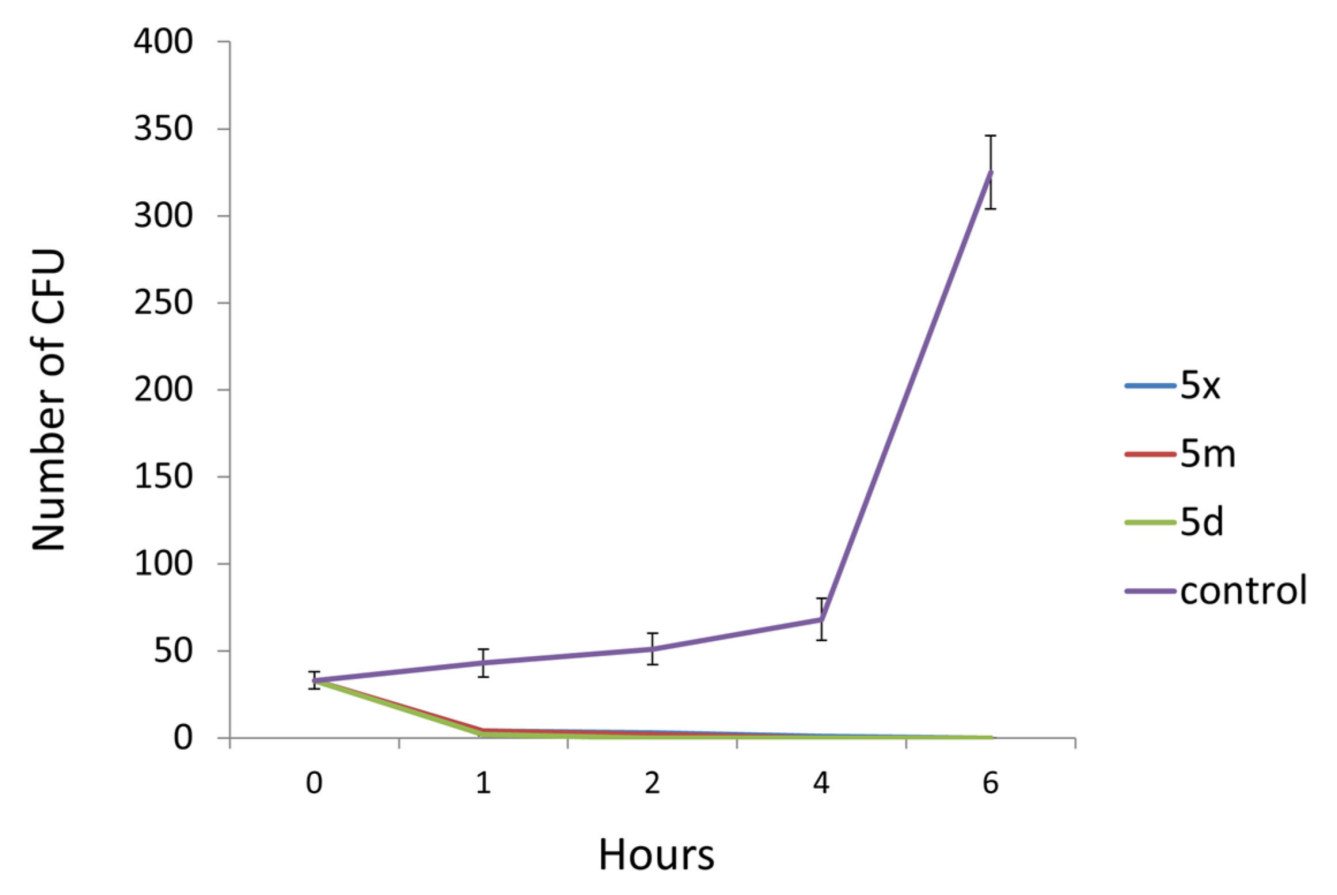
Number of *P. aeruginosa* CFU after different time intervals of antimicrobial treatment with MBC of tested compounds.

The bactericidal nature of three more active compounds, **5d, 5m**, and **5x** against *P. aeruginosa* was determined by a time-kill curve study. The treatment with the MBC of all selected compounds drastically reduced the number of *P. aeruginosa* CFU (Figure 4). Even after 1 h of treatment with compounds **5d**, **5m**, and **5x**, the number of bacterial CFU was reduced by more than 90%, while the 2-h treatment induced a reduction of more than 94%. After 6h, none of the *P. aeruginosa* colonies treated with the selected compounds (**5d, 5m, and 5x**) remained viable. 

### 2.5. Antifungal Activity

Compounds of both groups were then evaluated for antifungal activity. All compounds (indole-based thiazole derivatives) (Table 4) showed antifungal activity, with MIC in the range of 0.06–1.88 mg/mL and MBC of 0.12–3.75 mg/mL. The order of their activity can be presented as follows: **5x > 5l > 5d > 5b > 5m > 5v > 5n = 5o > 5e > 5a = 5q > 5u > 5s > 5i > 5f > 5c**. Thus, the most active compound appeared, again, to be compound **5x**, with MIC and MBC of 0.06–0.12 mg/mL and 0.12–0.23 mg/mL, respectively, while compound **5c** showed the lowest activity, with MIC in the range of 0.12–1.88 mg/mL and MBC of 0.23–3.75 mg/mL. 

It is worth noting that 7 (**5d, 5e, 5l, 5o, 5u, 5v**, and **5x**) out of 16 compounds appeared to be more potent than ketoconazole against some fungal species. Thus, compound **5x** was more active against all fungal species than bifonazole and ketoconazole, while compounds **5d** was almost equipotent to ketoconazole. Compounds **5l** and **5v** exhibited higher activity than both reference drugs against *A. niger* and *A. versicolor*, while **5l, 5q**, and **5v** appeared to be equipotent to ketoconazole against the most resistant strain, *A. fumigatus.* Additionally, compound **5q** was equipotent to ketoconazole against *A. niger*, while **5l** was almost equipotent to bifonazole against *T viride* and more potent than ketoconazole (MIC/MBC of 0.12/0.23 mg/mL) against *P.v.c.* On the other hand, compounds **5a, 5d, 5e, 5m, 5o** and **5u** were found to be equipotent to ketoconazole against *A.versicolor.* It is worth noting that all compounds exhibited superior activity to that of ketoconazole against *T. viride*. Furthermore, compound **5x** demonstrated higher activity than bifonazole against five species, being almost equipotent against *A. fumigatus*. It is known that clinically, aspergillosis, due to *A. fumigatus*, represents 90% of systemic fungal infections in immunocompromised patients.

The structure-activity relationships revealed that, like in case of antibacterial activity, the presence of a propan-2-ylidenhydrazine substituent in position 2 of the thiazole ring (**5x**) is very beneficial for antifungal activity. Additionally, the presence of a methyl group in positions 2 and 6 of the indole ring and an amino group in position 2 of the thiazole ring (**5l**) appeared to be beneficial. The removal of an Me group from position 2 of the indole ring led to compound **5d** with decreased activity. The replacement of a 6-Me group by a 5-OMe substituent in the indole ring led to a less active compound **5b** compared to compound **5d,** while the presence of a 6-Cl substitution in the indole ring and a 2-NH_2_ in the thiazole ring (**5m**) further decreased the activity. A negative influence on antifungal activity was observed with the presence of a methylamino group instead of an amino group in position 2 of the thiazole ring, the replacement of a 6-Cl substituent with 5-OMe, as well as the removal of a 2-Me group in the indole ring, leading to a less potent compound **5v.** Furthermore, the combination of a 2-NH_2_ substitution in the thiazole ring with 6-OMe in the indole ring (**5c**) appeared to be detrimental to antifungal activity. In general, it was observed that the presence of a methoxy group in either position 5 or 6 of the indole ring, in combination with 2-NH_2_ substitution in thiazole ring, has a negative effect on the antifungal activity of the compounds.

Regarding the second group, methylindole derivatives, all compounds showed promising antifungal activity, (Table 5) with MIC of 0.06–1.88 mg/mL and MFC in the range of 0.12–3.75 mg/mL. The order of their activity is: **5r > 5t = 6c = 6f > 5g > 6d > 5k > 6e > 5p = 6b > 5h > 5w.** Thus, the best activity in the group of methylindole derivatives was achieved for compound **5r**, with MIC in the range of 0.12–0.47 mg/mol and MFC of 0.23–0.94 mg/mL, whereas the less active compound appeared to be **5w** (MIC and MFC of 0.12–1.88 mg/mL and 0.23–3.75 mg/mL, respectively). The most sensitive fungal was found to be *T. viride*, followed by *A. niger*, while *P.v.c* was the most resistant one, followed by *P. funiculosum*. Bifonazole exhibited activity, with MIC in the range of 0.10–0.20 mg/mL and MFC of 0.20–0.25 mg/mL, while ketoconazole had MIC of 0.20–1.00 mg/mL and MFC of 0.30–1.50 mg/mL. Most of compounds displayed activity almost equipotent to ketoconazole against almost all fungi tested. Thus, compound **5g** showed promising activity against *A. niger*, with MIC/MFC of 0.06/0.12 mg/mL, better than bifonazole; compounds **5r** and **5w** appeared to be (MIC/MFC 0.12/0.23 mg/mL) almost equipotent to bifonazole (MIC/MFC 0.15/0.2 mg/mL); while compounds **5g**, **6b–6e,** and **6g**, with MIC/MFC of 0.23/0.47 mg/mL, were equipotent to ketoconazole. On the other hand, compounds **5g, 5r**, **5w**, as well as **6b** and **6d**, with MIC and MFC of 0.23 mg/mL and 0.47 mg/mL, respectively, appeared to be equipotent to ketoconazole against *A.versicolor,* whereas compounds **5h, 5p**, **5r** and **6e** appeared to be equipotent to ketoconazole against the second most resistant fungal, *P. funiculosum*. Finally, all compounds appeared to be more potent than ketoconazole against *T viride*, except **6b.**

The study of the structure-activity relationships of methylindole derivatives revealed that the presence of a 2-Me substituent in the methylindole ring, as well as 2-NH_2_ and 5-Me groups in the thiazole ring (**5r**) is very beneficial for antifungal activity. The removal of a methyl group from position 5 of the thiazole moiety of compound **5r** was detrimental to antifungal activity, leading to a less active compound (**5w**). The introduction of a methyl group in position 5 of the methylindole ring, as well as the replacement of a 2-NH_2_ group with a methylamino and removal of a 5-Me group of thiazole ring (**5t**) slightly decreased the activity. It should be mentioned that an *N*-(2-amino-3-acetylpyrazine-2-carboxylic acid substituent in position 2 of the thiazole ring (**6c**), as well as 2-(2-amino-2-oxopropyl-benzoic acid (**6f**), had the same influence on antifungal activity as previous substituents. In general, the presence of 2,5-di-Me groups in the methylindole ring, in combination with 2-NH_2_ substitution in the thiazole ring (**5p**), an *N-*(2-methoxyethyl)-2-oxopropanamide (**6b**) substituent, as well as 2-Me or 5-OMe substitution in the benzylindole ring and 2-NMe substitution in the thiazole ring (**5h**), had a negative effect on antifungal activity.

Thus, from all mentioned above, it is clear that antifungal activity of these compounds depends on substituents and their position in the methylindole ring, as well as on the nature of the substituents in the thiazole ring. 

Finally, it worth noting that methylindole derivatives displayed better antifungal activity than antibacterial but not better than indole derivatives.

### 2.6. Docking Studies

#### 2.6.1. Docking Studies to Antibacterial Targets

In order to estimate the probable mechanism of antibacterial action of the tested compounds, molecular docking studies on different antibacterial targets (DNA topo IV*, E coli* primase, Gyrase, Thymidyl kinase, *E. coli* MurB) were performed. The results are presented in Table 6 and reveal that the calculated free energy of binding to *E. coli* MurB enzyme (PDB:2Q85) of indole-based thiazolidinones was lower than that of the other enzymes for each compound. Therefore, it may be concluded that the inhibition of *E. coli* MurB enzyme is probably the most suitable antibacterial mechanism in the tested compounds.

**Figure 5:**
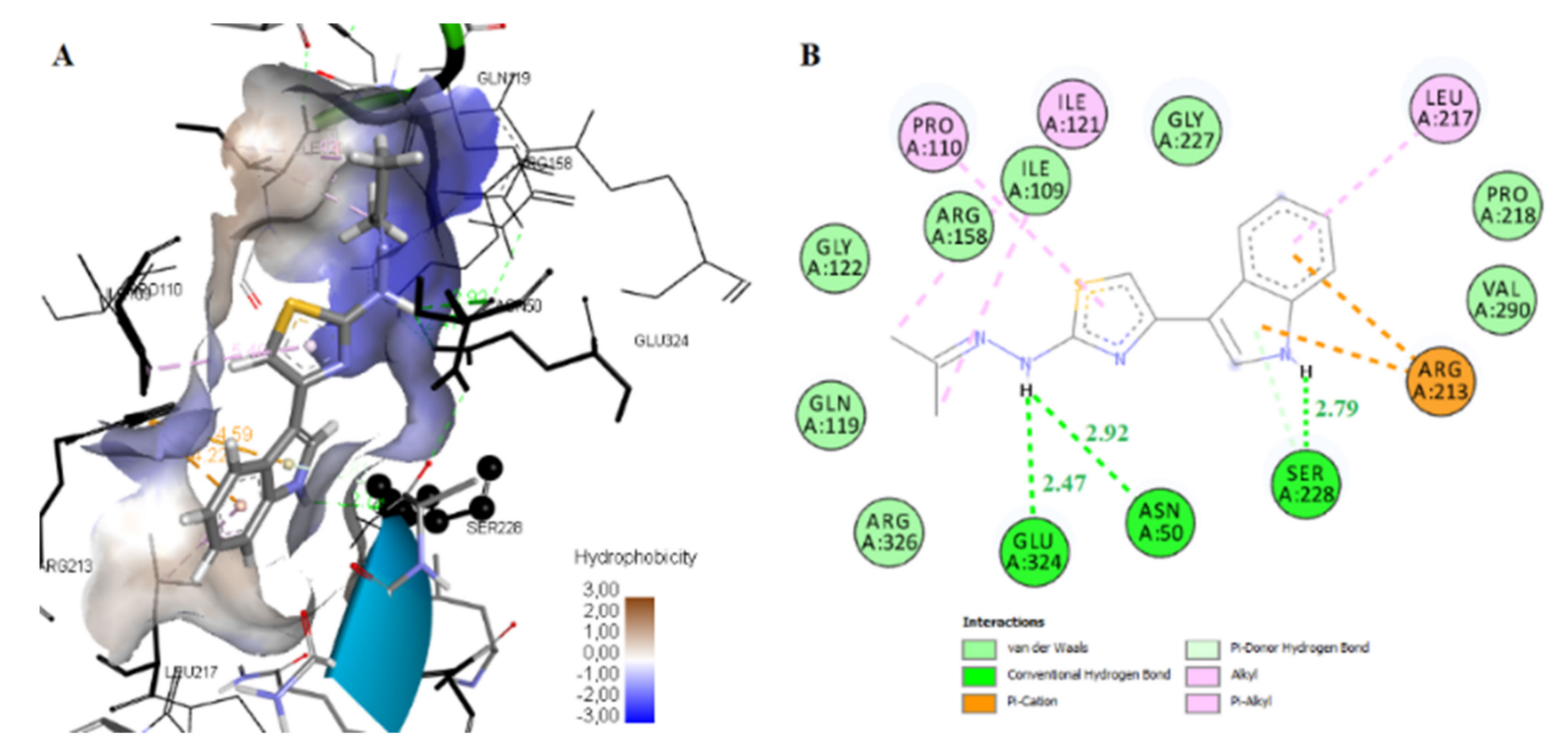
Binding mode in 3D (**A**) and 2D (**B**) of compound **5x** into the enzyme-active side of *E. coli* MurB (PBD: 2Q85). Green dotted lines represent the hydrogen bonds; pink-colored dotted lines and light green spheres represent pi-pi and hydrophobic interactions, respectively.

The most active compound, **5x**, exhibited the lowest free binding energy (−10.74 Kcal/mol) ) forming three hydrogen bonds: between the hydrogen of indole moiety and the oxygen atom of the side chain of the amino acid Ser228 (distance 2.79 Å), and another two hydrogen bonds between the hydrogen of NH group of the side chain of the compound and the oxygen atoms of the residues Asn50 and Glu324 respectively (2.92 and 2.47 Å respectively). The compound is directed into the *E. coli* MurB enzyme-active site, into a “channel” composed of amino acids Ile121, Pro110, Ile109, Arg158, Gly122, Gln119, and Arg326, with which it interacts hydrophobically. All these interactions stabilize the complex compound enzyme (Figure 5). It is important to highlight that the hydrogen bond with the residue, Ser228, is crucial for the inhibitory action of this compound because this residue takes part in the proton transfer at the second stage of peptidoglycan synthesis. Hydrogen bond interactions with this residue were also observed for most of the compounds (Table 6).

#### 2.6.2. Docking to Antifungal Targets

All compounds and the reference drug ketoconazole were docked to lanosterol 14α-demethylase from pathogenic yeast *C*. *albicans* (CYP51_Ca_) and dihydrofolate reductase in order to predict the possible mechanism of antifungal action. According to the docking score, it seems that CYP51_Ca_ was the most suitable enzyme for antifungal potency (Table S2).

**Figure 6:**
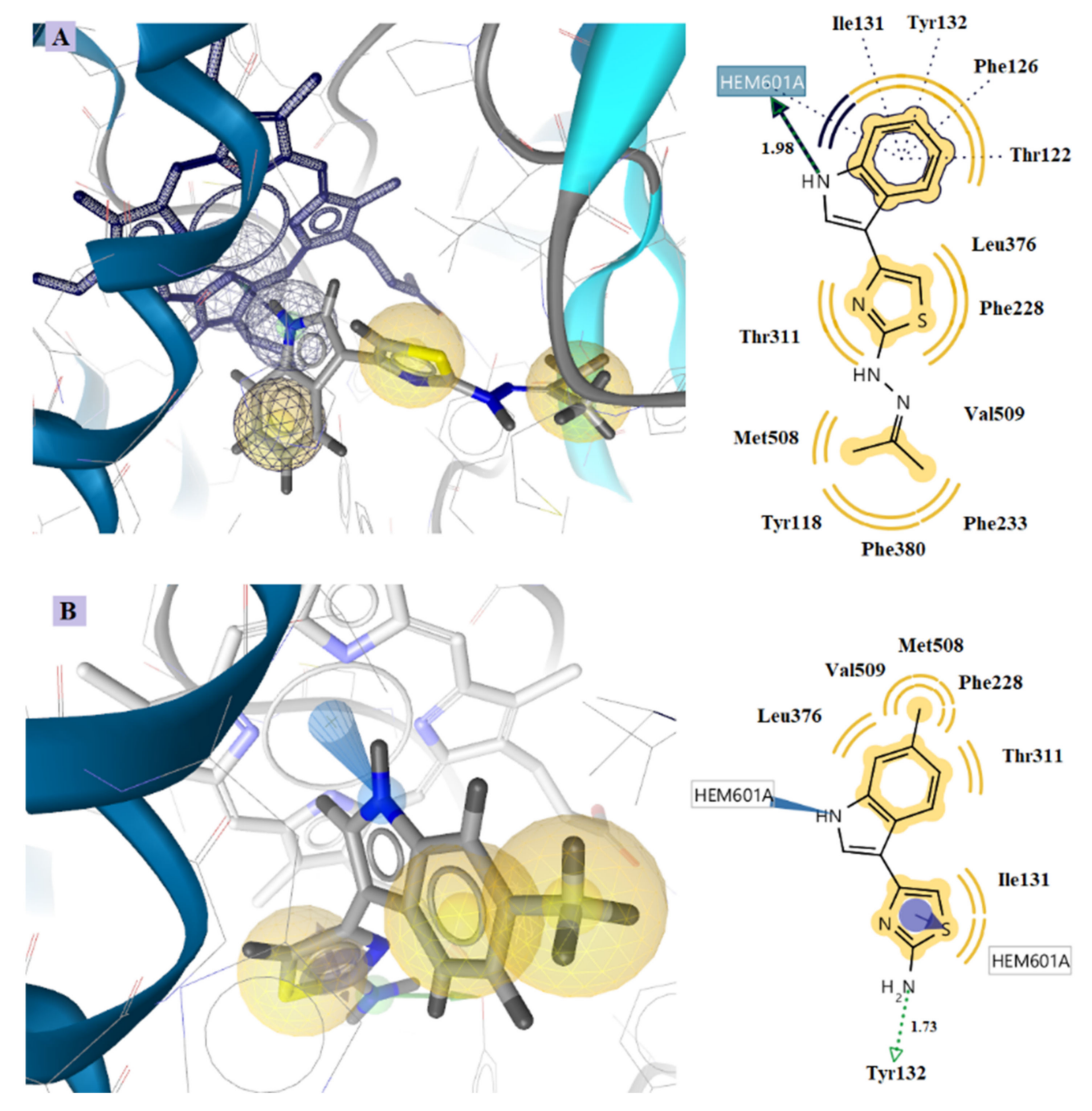
Docking of the most active compounds **5x** (**A**) and **5d** (**B**) to CYP51Ca receptor. Red and green dotted arrows indicate H-bond, blue arrows aromatic interactions and yellow spheres hydrophobic interactions.

**Figure 7:**
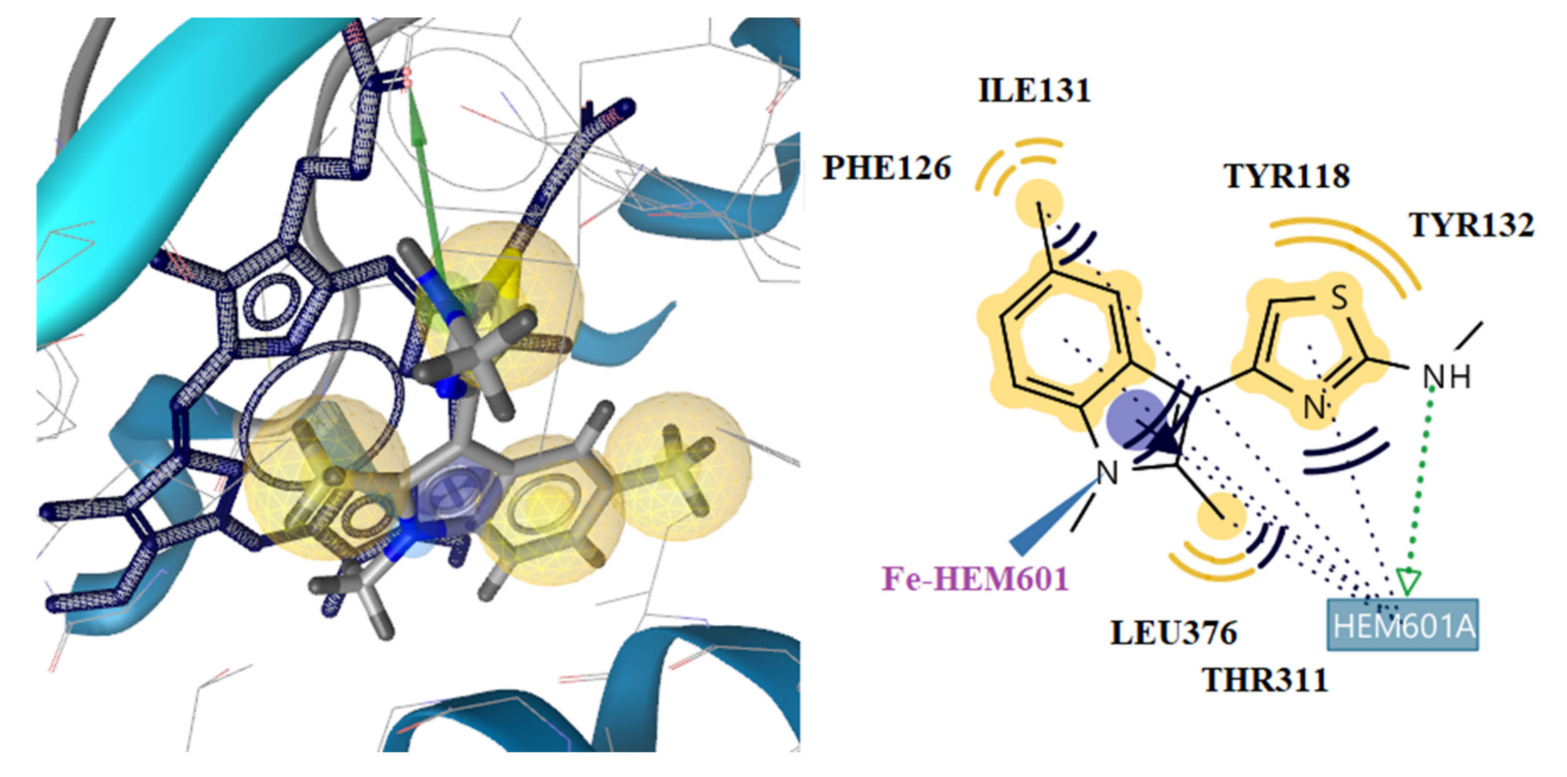
Docking of compound **5t** to CYP51_Ca_ receptor. Red and green dotted arrows indicate H-bond, blue arrows aromatic interactions and yellow spheres hydrophobic interactions.

Docking studies revealed that all the tested compounds may bind CYP51_Ca_ enzymes in an analogous mode to that of reference drug ketoconazole (Figure 6 and Figure 7). Compound **5x** showed the lowest free binding energy of −12.55 kcal/mol. This value is particularly due to the hydrogen bond formation between the nitrogen atom of the indole ring and the heme group of the enzyme (distance 1.98 A, Figure 6A). Hydrophobic interactions with the residues, Tyr118, Thr122, Phe126, Ile131, Tyr132, Phe288, Phe233, Thr311, Leu376, Phe380, Met508, and Val509 also contribute to the stability of the complex enzyme compound.

Furthermore, compound **5d** is placed inside the enzyme on the side of the heme group, binding by hydrophobic and aromatic interactions to the Fe of the heme group. This interaction with the Fe is stronger than hydrogen bonds, indicating the high inhibitory activity of this compound (Figure 6B). The benzene ring of ketoconazole also forms aromatic and hydrophobic interactions with the heme (Figure 7). However, compounds **5x** and **5d** form more stable complexes with the enzyme due to their stronger hydrogen bond formation and the interaction with the Fe of heme, respectively. This is probably the reason why these compounds have better antifungal activity than ketoconazole.

The results of docking of methyl indole derivatives are shown in Table S3.

**Figure 8:**
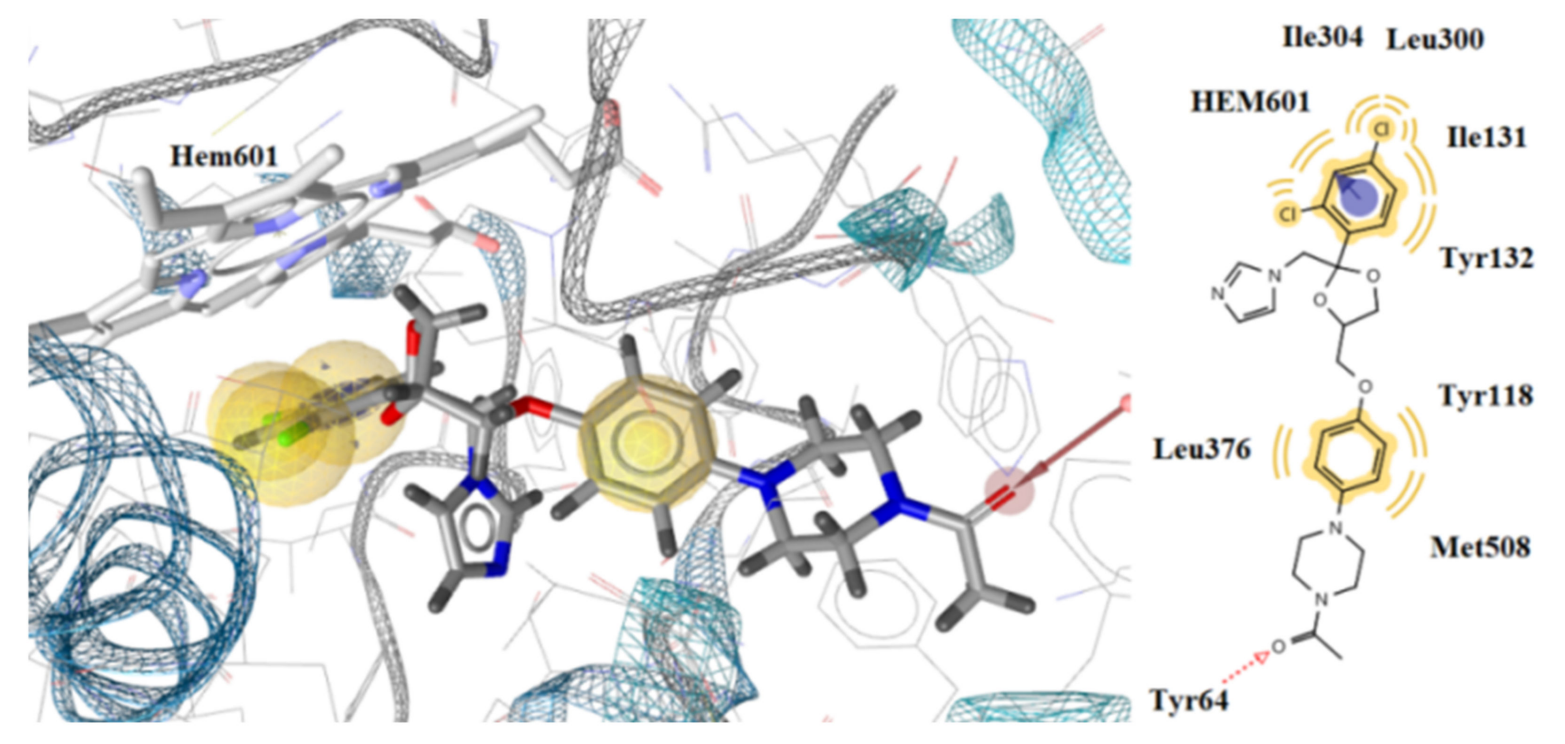
Docking of ketoconazole to CYP51_Ca_ receptor. Red and green dotted arrows indicate H-bond, blue arrows aromatic interactions and yellow spheres hydrophobic interactions.

According to the docking studies, all the tested methylindole-based thiazolidinone derivatives may bind to CYP51_Ca_ enzymes in an analogous mode to that of reference drug ketoconazole (Figure 8). Compound **5t** showed the lowest free binding energy, −11.28 kcal/mol. This value is particularly due to the hydrogen bond formation between the nitrogen atom of the methylindole ring of the compound and the hem group of the enzyme (distance 2.53 A, Figure 7). Hydrophobic interactions with the residues, Tyr118, Phe126, Ile131, Tyr132, Thr311, Leu376, and hem also contribute to the stability of the complex enzyme compound.

**Figure 9:**
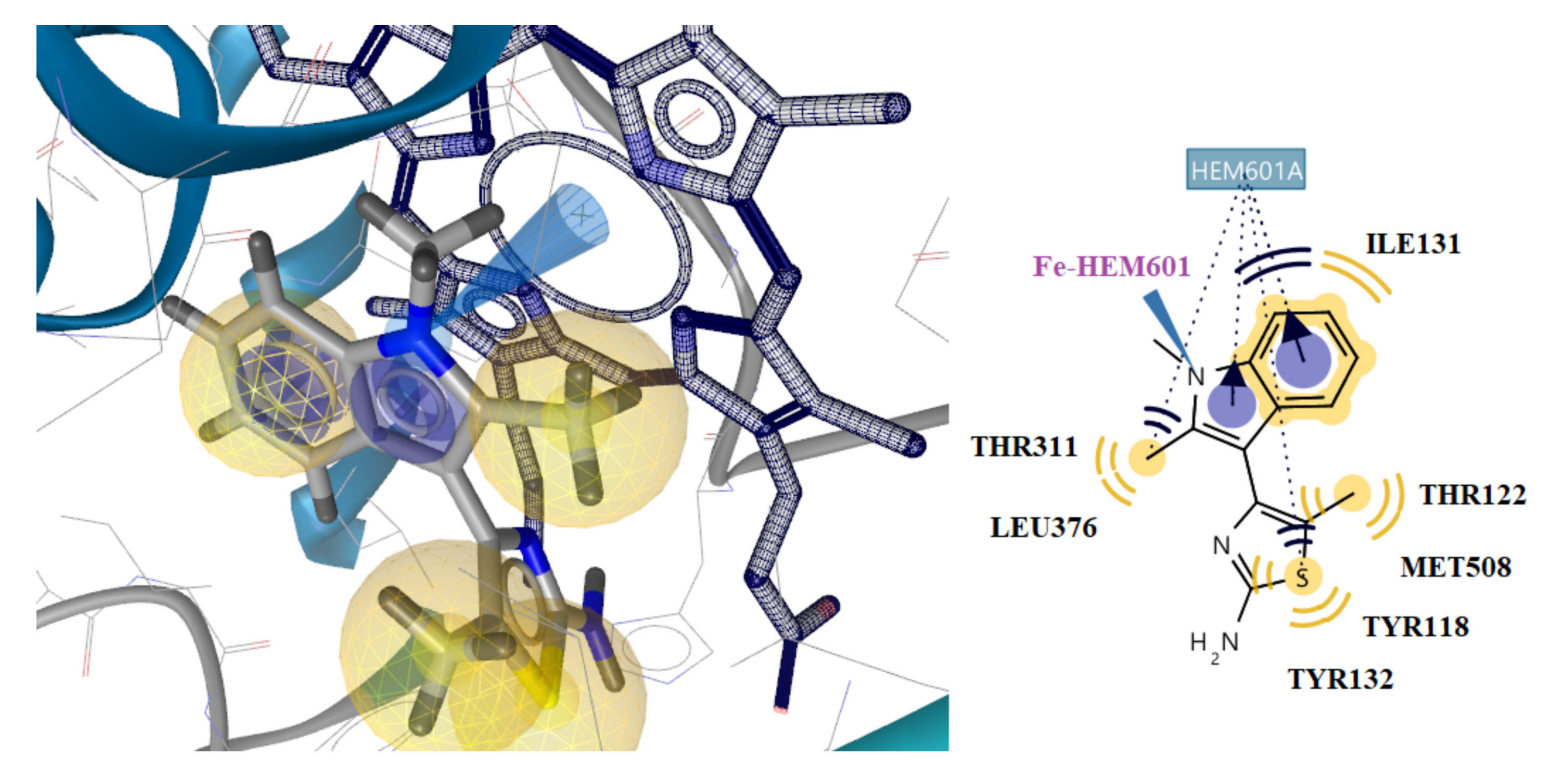
Docking of the most active compound **5r** to CYP51_Ca_ receptor. Red and green dotted arrows indicate H-bond, blue arrows aromatic interactions and yellow spheres hydrophobic interactions.

Furthermore, compounds **5t** and **5r** are placed inside the enzyme on the side of the hem group, binding to the Fe of hem through hydrophobic and aromatic interactions. (Figure 7 and Figure 9). However, compounds **5t** and **5r** form more stable complexes with the enzyme due to their stronger interaction with the Fe of hem than the interaction observed between the benzene of ketoconazole and hem (Figure 8). This is probably the reason why these compounds have better antifungal activity than ketoconazole.

### 2.7. Drug-Likeness

In order to see whether our compounds can be bioactive oral drug candidates, the prediction of drug-likeness was performed based on several rules [69,70,71,72,73].

**Figure 10:**
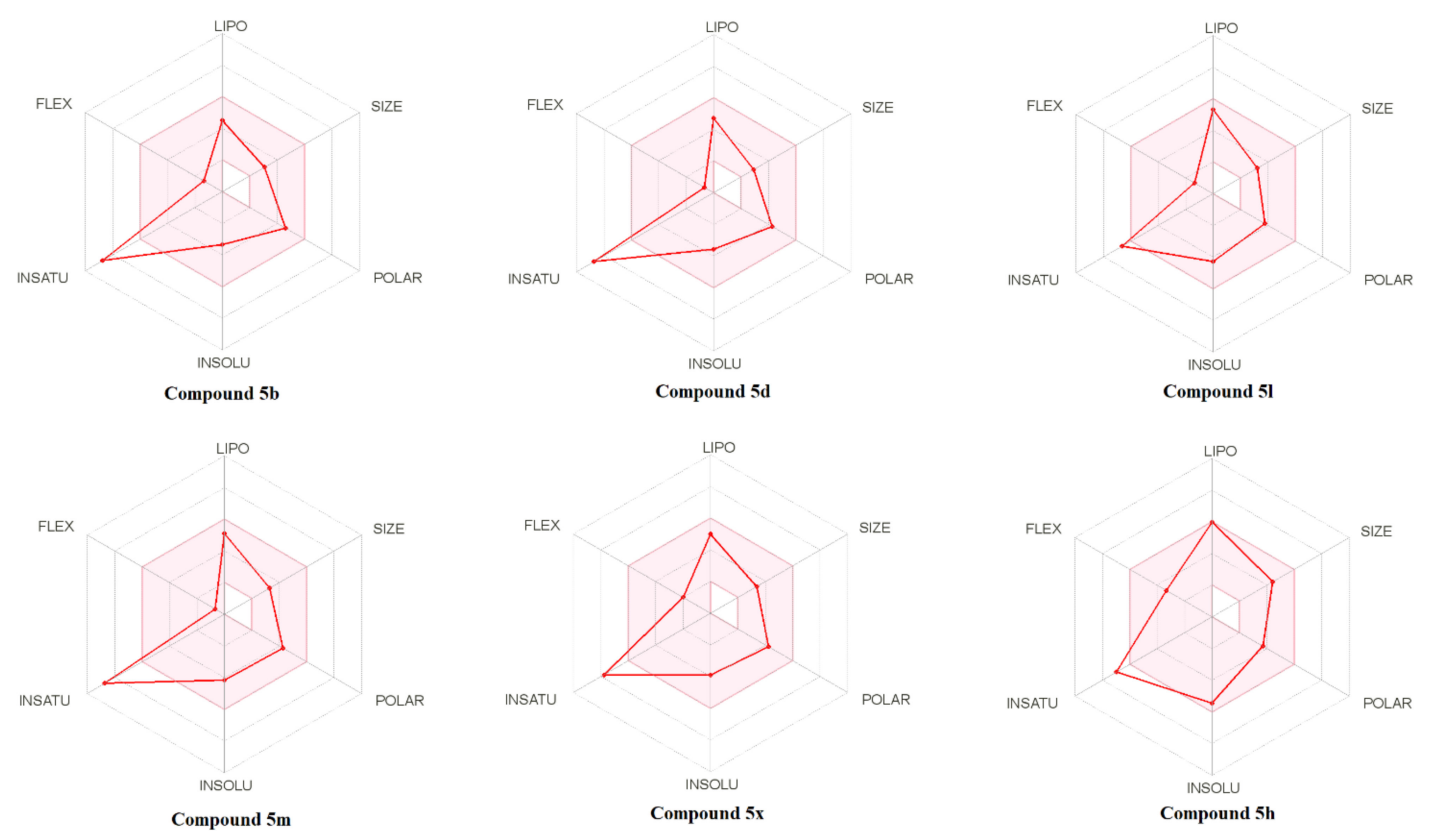
Bioavailability radar of the tested compounds. The pink area represents the optimal range for each property for oral bioavailability, (lipophilicity (LIPO): XLOGP3 between −0.7 and +5.0, molecular weight (SIZE): MW between 150 and 500 g/mol, polarity (POLAR): TPSA between 20 and 130 Å^2^, solubility (INSOLU): log S not higher than 6, saturation (INSATU): fraction of carbons in the sp3 hybridization not less than 0.25, and flexibility (FLEX): no more than 9 rotatable bonds).

The bioavailability and drug-likeness scores of all compounds are shown in Table S4. According to Table S4 none of the compounds violated any rule, and their bioavailability score was about 0.55. Moreover, all compounds displayed moderate-to-good drug-likeness scores (−0.63 to 0.29). Figure 10 presents the bioavailability radar of some of the compounds. The best of the in-silico predictions results was achieved for compounds with a drug-likeness score of 0.29.

### 2.8. Cytotoxicity Assessment

**Figure 11:**
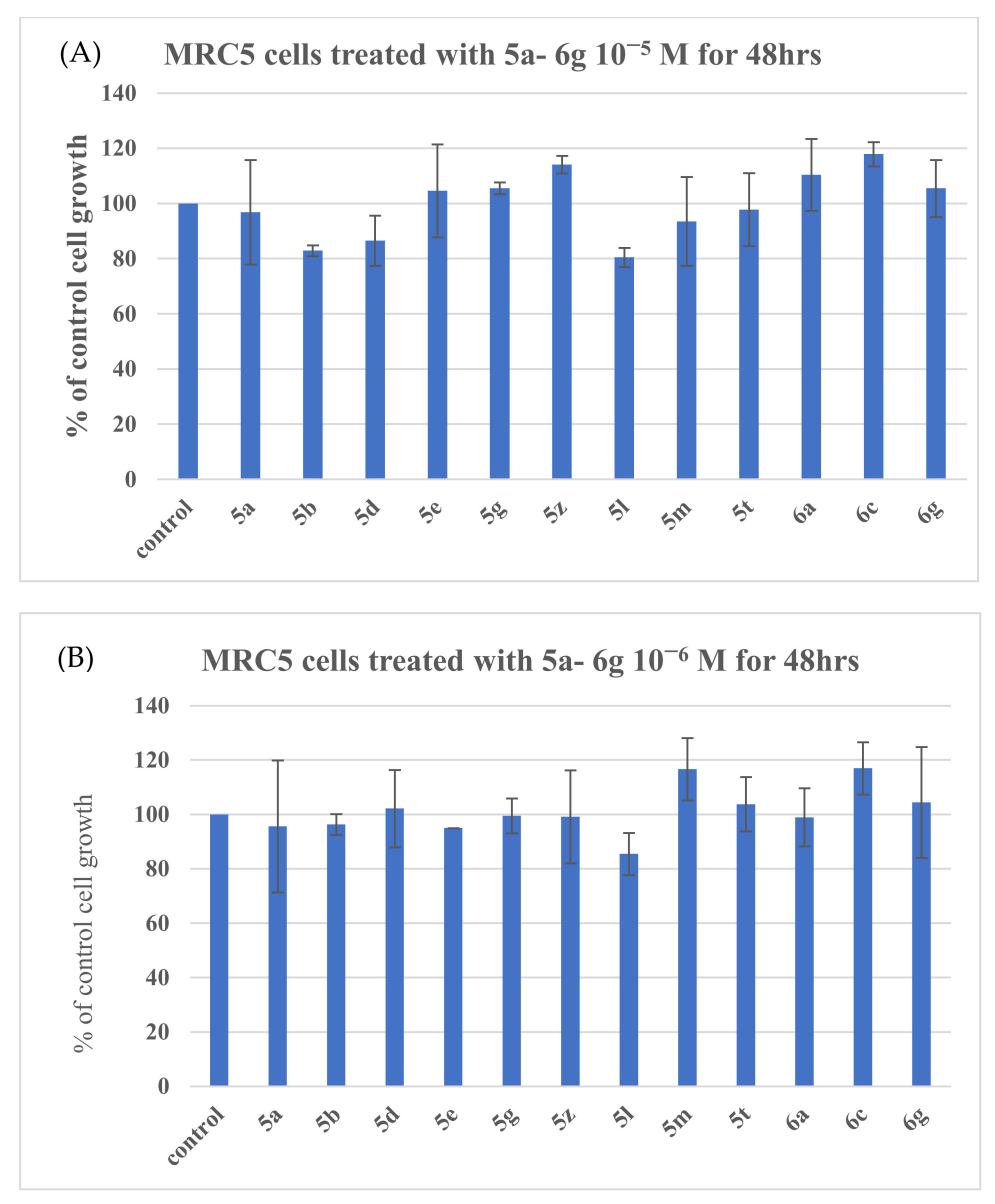
Assessment of cell proliferation of MRC-5 cells exposed to different compounds in culture. MRC-5 cells grown in culture were separately incubated with each of the compounds at concentrations 1 × 10^−5^ M (10 μΜ) (**panel A**) and 1 × 10^−6^ M (1 μM) (**panel B**) for 48 h [44].

The assessment of cellular cytotoxicity of the compounds in normal human MRC-5 cells was evaluated at two concentrations in culture, i.e., 1 × 10^−5^ M (Figure 11A,B) and 1 × 10^−6^ M (Figure 11A,B). No substantial effect on cell proliferation after 48 h exposure was observed in cultures, since the growth was ≥80% for all the tested agents compared to control untreated cultures (Figure 11A, B). Moreover, the percentage of dead cells accumulated in the cultures was very low, since the maximum number did not exceed that of 2–2.5% (Data not shown).

## 3. Materials and Methods

### 3.1. General Procedure for the Synthesis of 3- (α-chlorouracil) -Indoles **2a–q**

Starting indoles **1a, 1d, 1j,** and **1f** were commercially available, while starting indoles **1b, 1c, 1e,** and **1i** were obtained by decarboxylation of the corresponding indol-2-yl-carboxylic acids [74], and indole **1g** was obtained according to [75]. Indoles **1h** and **1i** were prepared by alkylation of indoles **1f** and **1m** with benzyl chloride and methyl iodide, respectively, according to [76]. Finally, indoles **1k, 1j и**, and **1o** were obtained following Chapman et al. [77].

### 3.2. General Method for Synthesis of 3- (α-chlorouracil) Indoles 

To a stirred solution of the corresponding indoles, **1a–o** (0.1 mol), and pyridine (8.1 mL, 0.1 mol) in toluene (250 mL) at 55–60 °C, the corresponding acyl chlorides, **2a–b**, (0.1 mol) were added dropwise over 1 h. The mixture was stirred at the same temperature for another 1 h, cooled to room temperature, poured into water (1 L), and kept for 3 h at a temperature of 5–8 °C. The precipitate that formed was filtered out, washed on a filter with water (0.5 L), dried, and recrystallized from an appropriate solvent.

3-(α-Chloroacetyl) indole (**3a**). Yield 49%; mp. 231–232 °C (from ethanol), according to [63] mp. is 230–232 °C.

5-Methoxy-3- (α-chloroacetyl) indole (**3b**). Yield 56%; mp.193–194 Co (from aqueous acetone), ^1^H-NMR (300 MHz, DMSO-d6, ppm) 11.73 (s, 1H NH), 8.15 (s, 1H, C2-H), 7.68 (s, 1H, C4-H), 7.32 (d, *J* = 9.0 Hz, 1H, C6), 6.75 (d, *J* = 6.0 Hz, 1H, C7-H) 4.63 (s, 2H, –CH_2_–Cl), 3.81 (s, 3H, 5-CH_3_O). Anal.Calcd.For C_11_H_10_ClNO_2_ (%):C, 59.07; H, 4.51; N, 6.26. Found (%): C, 59.03; H, 4.55; N. 6.30.

6-Methoxy-3-(α-chloroacetyl) indole (**3c**). Yield 52%; mp.175–177 °C (from aqueous acetone), ^1^H-NMR (300 MHz, DMSO-d6, ppm) 11.61 (s, 1H NH), 8.10 (s, 1H, C2-H), 8.04 (d, *J* = 3.0 Hz, 1H, C4-H), 6.92 (d, *J* = 3.0 Hz, 1H C7-H), 6.80 (dd, *J* = 9.0Hz, J = 6.0Hz, 1H, C5-H), 4.60 (s, 2H, –CH_2_–Cl), 3.83 (s, 3H, 6-CH_3_O). Anal.Calcd.For C_11_H_10_ClNO_2_ (%):C, 59.07; H, 4.51; N, 6.26. Found (%): C, 59.04; H, 4.56; N. 6.29.

6-Methyl-3-(α-chloroacetyl) indole (**3d**). Yield 56%, mp.163–164 °C (from ethanol), ^1^H-NMR (300 MHz, DMSO-d6, ppm) 11.59 (s, 1H NH), 7.93 (s, 1H, C2-H), 7.74 (d, *J* = 6.0 Hz, 1H, C4-H), 7.13 (s, 1H, C7-H), 6.95 (d, *J* = 9.0 Hz, 1H, C7-H), 4.55 (s, 2H, –CH_2_–Cl), 2, 48 (s, 3H, 6-CH_3_). Anal.Calcd.For C_11_H_10_ClNO (%):C, 63.62; H, 4.85; N, 6.75. Found (%): C, 63.58; H, 4.90; N. 6.70.

5-Fluoro-3- (α-chloroacetyl) indole (**3e**). Yield 64%; mp.165–166 °C (from ethanol), ^1^H-NMR (300 MHz, DMSO-d6, ppm) 12.01 (s, 1H NH), 8.34 (d, *J* = 3.0 Hz, 1H, C2-H), 7.84 (dd, *J* = 12.0 Hz, *J* = 3.0Hz, 1H, C4-H), 7.43 (dd, *J* = 9.0 Hz, *J* = 3.0 Hz, 1H, C7-H), 6.96 (m, 1H, C6-H), 4.66 (s, 2H, –CH_2_–Cl). Anal.Calcd.For C_10_H_7_ClFNO (%):C, 56.76; H, 3.33; N, 6.62. Found (%): C, 56.72; H, 3.35; N. 6.65.

2-Methyl-5-methoxy-3- (α-chloroacetyl) indole (**3f**). Yield 66%; mp.151–152 °C (from iso-propanol). ^1^H-NMR (300 MHz, DMSO-d6, ppm) 11.60 (s, 1H NH), 7.46 (s, 1H, C4-H), 7.21 (d, *J* = 6.0 Hz, 1H, C6-H), 6.74 (d, *J* = 4.0Hz, 1H, C7-H), 4.65 (s, 2H, –CH_2_–Cl), 3.83 (s, 3H, 5-CH_3_O), 2.69 (s, 3H, 2-CH_3_). Anal.Calcd.For C_12_H_12_ClNO_2_ (%):C, 60.64; H, 5.09; N, 5.89. Found (%): C, 60.66; H, 5.12; N, 5.85.

2,6-Dimethyl-3-(α-chloroacetyl) indole (**3g**). Yield 59%; mp. 163–165 °C (from ethanol), ^1^H-NMR (400 MHz, DMSO-d6, ppm) 11.61 (s, 1H NH), 7.74 (d, *J* = 6.0 Hz, 1H, C4-H), 7.13 (s, 1H, C7-H), 6.95 (d, *J* = 9.0Hz, 1H, C7-H), 4.65 (s, 2H, –CH_2_–Cl), 2.71 (s, 3H, 2-CH_3_). 2.43 (s, 3H, 6-CH_3_). Anal.Calcd.For C_12_H_12_ClNO (%):C, 65.02; H, 5.46; N, 6.32. Found (%): C, 65.06; H, 5.49; N, 6.35.

2-Methyl-5-chloro-3-(α-chloroacetyl) indole (**3h**). Yield 61%; mp. 134–135 °C (from ethanol), ^1^H-NMR (300 MHz, DMSO-d6, ppm) 11.99 (s, 1H NH), 7.94 (s, 1H, C4-H), 7.32 (d, *J* = 9.0 Hz, 1H, C7-H), 7.08 (dd, *J* = 9.0 Hz, *J* = 3.0 Hz, 1H, C6-H), 4.68 (s, 2H, –CH_2_–Cl), 2.73 (s, 3H, 2- CH_3_). Anal.Calcd.For C_11_H_9_Cl_2_NO (%):C, 54.57; H, 3.75; N, 5.79. Found (%): C, 54.60; H, 3.80; N, 6.75.

5-Ethoxy-3-(α-chloroacetyl) indole (**3i**). Yield 59%; mp. 149–151 °C (from ethanol), 1H-NMR (300 MHz, DMSO-d6, ppm) 11.73 (s, 1H NH), 8.25 (s, 1H, C2-H), 7.58 (s, 1H, C4-H), 7.52 (d*, J* = 9.0 Hz, 1H, C6), 6.81 (d, *J* = 6.0 Hz, 1H, C7-H) 4.59 (s, 2H, –CH_2_–Cl),), 4.12 (q, J = 20.0 Hz, 2H, -O-CH2 -), 1.41 (t, *J* = 6.0 Hz, 3H, CH_3_–CH_2_–O). Anal.Calcd.For C_12_H_12_ClNO_2_ (%):C, 60.64; H, 5.09; N, 5.89. Found (%): C, 60.62; H, 5.11; N, 5.86.

2-methyl-3-(α-chloroacetyl) indole (**3j**). Yield 74%; mp.221–222 °C (from acetonitrile), according to [6] so pl. 220–221 Co. ^1^H-NMR (300 MHz, DMSO-d6, ppm) 11.84 (s, 1H NH), 7.89 (d, *J* = 3Hz, 1H, C4-H), 7.34 (m, 1H, C7-H), 7.11 (m, 2H, C5-H, C6-H), 4.69 (s, 2H, –CH2–Cl), 2.74 (d, *J* = 3.0Hz, 3H, 2-CH_3_). Anal.Calcd.For C_11_H_10_ClNO (%):C, 63.62; H, 4.85; N, 6.75. Found (%): C, 63.64; H, 4.89; N, 6.73.

2-Methyl-5-fluoro-3-(α-chloroacetyl) indole (**3k**). Yield 72%; mp. 168–170 °C (from ethanol). ^1^H-NMR (400 MHz, DMSO-d6, ppm) 11.90 (s, 1H NH), 7.65 (dd, *J* = 12.0 Hz, *J* = 3.0 Hz, 1H, C4-H), 7.31 (dd, *J* = 9.0 Hz, *J* = 3.0 Hz, 1H, C7-H), 6.88 (m, 1H, C6-H), 4.67 (s, 2H, –CH_2_–Cl), 2.72 (s, 3H, 2-CH_3_). Anal.Calcd.For C_11_H_9_FClNO (%):C, 58.55; H, 4.02; N, 6.21. Found (%): C, 58.52; H, 4.05; N, 6.24.

2-Methyl-3-(α-chloropropionyl) indole (**3l**). Output 65%; mp. 211–213 °C (from ethanol). ^1^H-NMR (400 MHz, DMSO-d6, ppm) 11.63 (s, 1H NH), 7.79 (d, *J* = 3Hz, 1H, C4-H), 7.24 (m, 1H, C7-H), 6.99 (m, 2H, C5-H, C6-H), 4.69 (s, 1H, CH_3_–CH–Cl), 2.74 (d, *J* = 3.0Hz, 3H, 2-CH_3_), 2.45 (d, *J* = 4.0Hz, 3H, CH_3_–CH–Cl). Anal.Calcd.For C_11_H_11_NO (%):C, 76.28; H, 6.40; N, 8.09. Found (%): C, 76.31; H, 6.4.5; N, 8.05.

### 3.3. General Procedure for the Synthesis of Indol-2-ylthiazoles **5a–x**

A mixture of the corresponding 3-(α-chlorouracil)-indole, **3a-q** (0.1 mol), thiourea **4a–c (**0.125 mol)**,** potassium iodide (0.01 mol), and sodium carbonate (0.3 mol) in absolute methanol (250 mL) was refluxed with stirring until completion of the reaction (monitoring by TLC), cooled to room temperature, poured into water (1L), and an aqueous ammonia solution was added to pH 10. The precipitate formed was filtered off, washed on a filter with water (0.5 L), dried, and recrystallized from an appropriate solvent.

4-(1*H*-indol-3-yl) thiazol-2-amine (**5a**). Yield 56%, mp.148–150 °C (from isopropanol), 1H-NMR (300 MHz, DMSO-d6, ppm) 10.95 (s, 1H NH), 7.90 (d, *J* = 3.0 Hz, 1H, C4-H), 7.57 (s, 1H, CH tiazol), 7.37 (d, *J* = 3.0 Hz, 1H, C2-H), 6.89 (m, 3H, C6-H, C7-H), 6.43 (m, 1H, C5-H), 6.38 (m, 2H, NH_2_). ^13^C-NMR (CDCl_3_; 125 MHz) δ ppm: 109.25, 111.23, 111.76, 119.52, 121.03, 121.85, 125.34, 125.44, 135.14, 135.98, 167.34 (C-NH_2_). Anal.Calcd.For C_11_H_9_N_3_S (%): C, 61.37; H, 4.21; N, 19.52. Found (%): C, 61.35; H, 4.22; N, 19.54.

4-(5-Methoxy-1*H*-indol-3-yl) thiazol-2-amine (**5b**). Yield 66%; mp. 182–183 °C (from isopropanol), ^1^H-NMR (300 MHz, DMSO-d6, ppm) 10.80 (s, 1H NH), 7.53 (d, *J* = 3.0 Hz, 1H, C2-H), 7.40 (s, 1H, C4-H), 7.26 (d, *J* = 3.0 Hz, 1H, C7-H), 6.74 (dd, *J* = 9.0 Hz, *J* = 3.0 Hz, 1H, C6-H), 6.61 (s, 2H, NH_2_), 6.49 (s, 1H, CH thiazol), 3.83 (s, 3H, 5-CH_3_O). ^13^C-NMR (CDCl_3_; 125 MHz) δ ppm: 55.84 (O-CH_3_), 108.16, 109.16, 109.87, 112.16, 112.97, 124.05, 128.11, 129.13, 135.26, 153.79 (C-O-CH_3_), 167.34 (C-NH_2_). Anal.Calcd.For C_12_H_11_N_3_OS (%): C, 58.76; H, 4.52; N, 17.13. Found (%): C, 58.78; H, 4.50; N, 17.11.

4-(6-Methoxy-1*H*-indol-3-yl) thiazol-2-amine (**5c**). Yield 54%; m.p.266–267 °C (from aqueous acetone), ^1^H-NMR (200 MHz, DMSO-d6, ppm) 10.70 (s, 1H NH), 7.76 (d, *J* = 10.0 Hz, 1H, C2-H), 7.43 (d, *J* = 2.0 Hz, 1H, C5-H), 6.87 (d, *J* = 4.0 Hz, 1H, C5-H), 6.85 (d, *J* = 2.0 Hz, 1H), 6.69 (d, *J* = 2.0 Hz, 2H, NH2) 6.43 (s, 1H, CH tiazole), 3.81 (s, 3H, 6-CH_3_O). ^13^C-NMR (CDCl_3_; 125 MHz) δ ppm: 55.69 (O-CH_3_), 96.24, 108.11, 108.23, 111.87, 117.58, 121.03, 124.17, 135.88, 135.94, 156.17 (C–O–CH_3_), 167.32 (C-NH_2_). Anal.Calcd.For C_12_H_11_N_3_OS (%): C, 58.76; H, 4.52; N, 17.13. Found (%): C, 58.74; H, 4.58; N, 17.08.

4-(6-Methyl-1*H*-indol-3-yl) thiazol-2-amine (**5d**). Yield 54%; m.p.166–168 °C (from isopropanol), ^1^H-NMR (200 MHz, DMSO-d6, ppm) 10.79 (s, 1H NH), 7.74 (d*, J* = 8.0 Hz, 1H, C2-H), 7.52 (d, *J* = 2.0Hz, 1H, C4-H), 7.16 (s, 1H, C5-H), 6.87 (d, *J* = 8.0 Hz, 2H, NH2), 6.80 (s, 1H, C7-H), 6.47 (s, 1H, CH tiazole), 2.60 (s, 3H, 6-CH_3_). ^13^C-NMR (CDCl_3_; 125 MHz) δ ppm: 21.68 (CH_3_), 110.13, 111.25, 111.97, 120.63, 121.77, 121.93, 124.37, 131.59, 135.40, 135.92, 167.11 (C–NH_2_). Anal.Calcd.For C_12_H_11_N3S (%): C, 62.86; H, 4.84; N, 18.33. Found (%): C, 62.81; H, 4.87; N, 18.36.

4-(5-Fluoro-1*H*-indol-3-yl) thiazol-2-amine (**5e**). Yield 56%; m.p.142–143 °C (from isopropanol), ^1^H-NMR (300 MHz, DMSO-d6, ppm) 11.06 (s, 1H NH), 7.64 (d, *J* = 6.0 Hz, 2H, C2-H, C4- H), 7.33 (dd, *J* = 9.0 Hz, *J* = 3.0 Hz, 1H, C6-H), 6.85 (t, *J* = 15.0 Hz, 1H, C7-H), 6.68 (s, 2H, NH_2_) 6.45 (s, 1H, CH tiazole). ^13^C-NMR (CDCl_3_; 125 MHz) δ ppm: 109.63, 109.82, 111.00, 112.46, 115.69, 124.58, 130.12, 132.22, 135.73, 157.60 (C-F), 167.24 (C-NH_2_). Anal.Calcd.For C_11_H_8_FN_3_S (%):C, 56.64; H, 3.46; F, 8.14; N, 18.01. Found (%): C, 56.62; H, 3.48; N, 18.03.

4-(5-Methoxy-2-methyl-1*H*-indol-3-yl) thiazol-2-amine (**5f**). Yield 63%; m.p.177–179 °C (from aqueous acetone), ^1^H-NMR (200 MHz, DMSO-d6, ppm) 10.59 (s, 1H, NH), 7.36 (d*, J* = 2.0 Hz, 1H, C4-H), 7, 15 (d, *J* = 4.0 Hz, 1H, C6-H), 6.62 (d, *J* = 6.0 Hz, 2H, -NH2), 6.56 (d, *J* = 4.0 Hz, 1H, C7-H), 6.25 (s, 1H, CH tiazole), 3.81 (s, 3H, 5-CH_3_O), 2.55 (s, 3H, 2-CH_3_). ^13^C-NMR (CDCl_3_; 125 MHz) δ ppm: 14.22 (CH_3_), 55.82 (O–CH_3_), 99.71, 108.12, 109.14, 112.11, 112.15, 127.61, 129.43, 135.78, 136.55, 154.21 (C–O–CH_3_), 167.33 (C–NH_2_). Anal.Calcd.For C_13_H_13_N_3_OS (%): C, 60.21; H, 5.05; N, 16.20. Found (%): C, 60.14; H, 5.03; N, 16.23.

4-(5-Methoxy-1,2-dimethyl-1*H*-indol-3-yl) thiazol-2-amine (**5g**). Yield 61%; m.p.130–131 °C (from acetone), ^1^H-NMR (300 MHz, DMSO-d6, ppm) 10.61 (s, 1H, NH indole), 7.43 (s, 1H, C4-H), 7.14 (s, ^1^H, NH-CH3) 7.13 (d, *J* = 3.0 Hz, 1H, C7-H), 6.62 (d, *J* = 9.0 Hz, 1H, C6), 6.29 (s, 1H, CH tiazole), 3.80 (s, 3H, 5-CH_3_O), 2.64 (s, 3H, 2-CH_3_). ^13^C-NMR (CDCl_3_; 125 MHz) δ ppm: 14.25 (CH_3_), 55.97 (O–CH_3_), 99.79, 109.63, 109.72, 112.01, 112.13, 127.45, 129.41, 135.44, 136.56, 154.22 (C–O–CH_3_), 167.31 (C–NH_2_). Anal.Calcd.For C_14_H_15_N_3_OS (%): C, 61.51; H, 5.53; N, 15.37. Found (%): C, 61.52; H, 5.42; N, 15.33.

4-(1-Benzyl-5-methoxy-2-methyl-1*H*-indol-3-yl)-*N*-methylthiazol-2-amine (**5h**). Yield 56%; mp. 194–196 °C (from isopropanol), ^1^H-NMR (300 MHz, DMSO-d6, ppm) 10.47 (s, 1H, NH), 7.71 (d, *J* = 6.0 Hz, 1H, C4-H), 7.03 (s, 1H, C7-H), 6.78 (d, *J* = 9.0 Hz, 1H, C5-H), 6.43 (s, 2H, NH_2_), 6.27 (s, 1H, CH tiazole), 2.91 (s, 3H, 2-CH_3_) 2.42 (s, 3H, 6-CH_3._^13^C-NMR (CDCl_3_; 125 MHz) δ ppm: 11.12 (CH_3_), 32.14 (N–CH_3_), 55.86 (O–CH_3_), 52.03, 99.12, 108.42, 109.26, 112.33, 125.76, 127.62, 127.64, 128.11, 128.12, 128.55, 129.69, 135.67, 137.12, 138.01, 154.21(C–O–CH_3_), 163.22 (C–N–CH_3_). Anal.Calcd.For C_21_H_21_N_3_OS (%): C, 69.39; H, 5.82; N, 11.56. Found (%): C, 69.42; H, 5.80; N, 11.59.

4-(5-Chloro-2-methyl-1H-indol-3-yl) thiazol-2-amine (**5i**). Yield 56%; m.p.127–128 °C (from isopropanol), ^1^H-NMR (200 MHz, DMSO-d6, ppm) 10.98 (s, 1H NH), 7.89 (d, *J* = 2.0 Hz, 1H, C4-H), 7.22 (d*, J* = 10.0 Hz, 1H, C7-H), 6.93 (d*, J* = 4.0 Hz, 1H, C6-H), 6.68 (s, 2H, NH_2_), 6.25 (s, 1H, CH tiazole), 2.51 (s, 3H, 2-CH_3_). ^13^C-NMR (CDCl_3_; 125 MHz) δ ppm: 14.28 (CH_3_), 32.05 (N–CH_3_), 55.83 (O–CH_3_), 99.15, 108.17, 109.66, 112.03, 112.07, 127.51, 129.44, 134.42, 135.73, 154.01 (C–O–CH_3_), 164.42 (C–NH_2_). Anal.Calcd.For C_14_H_15_N_3_OS (%): C, 61.51; H, 5.53; N, 15.37;. Found (%): C, 61.47; H, 5.56; N, 15.32.

4-(5-Methoxy-1,2-dimethyl-1*H*-indol-3-yl)-*N*-methylthiazol-2-amine (**5j**). Yield 59%; mp.203–204 °C (from isopropanol), ^1^H-NMR (200 MHz, DMSO-d6, ppm) 10.77 (s, 1H NH), 7.53 (d, *J* = 4.0 Hz, 1H, C2-H), 7.30 (dd*, J* = 14.0 Hz, J = 10.0 Hz, 2H, C4-H, C7-H), 6.69 (s, 2H, NH_2_), 6.63 (s, 1H, C6-H), 6.43 (s, 1H, CH tiazole), 4.07 (q, *J* = 20.0 Hz, 2H, –O–CH_2_–), 1.42 (t, *J* = 6.0 Hz, 3H, CH_3_-CH_2_-O). ^13^C-NMR (CDCl_3_; 125 MHz) δ ppm: 10.15 (CH_3_), 30.26 (N–CH_3_), 32.02 (N–CH_3_), 55.81 (O–CH_3_), 99.62, 108.51, 109.41, 106.78, 112.33, 128.34, 129.75, 135.72, 138.02, 154.11 (C–O–CH_3_), 164.40 (C-NH_2_). Anal.Calcd.For C_15_H_17_N_3_OS (%): C, 62.69; H, 5.96; N, 14.62. Found (%): C, 62.75; H, 5.91; N, 14.58.

4-(1,2-Dimethyl-1*H*-indol-3-yl)-N-methylthiazol-2-amine (**5k**). Yield 62%; mp.184–185 °C (from isopropanol), ^1^H-NMR (200 MHz, DMSO-d6, ppm) 10.71 (s, 1H, NH) 7.35 (d*, J* = 8.0 Hz, 1H, C4-H), 7.28 (d*, J* = 8.0 Hz, 1H, C5-H), 6.92 (m, 2H, C6-H, C7-H), 6.32 (s, 2H, NH_2_), 2.48 (s, 3H, 2-CH_3_), 2.12 (s, 3H, CH_3_-tiazole). ^13^C-NMR (CDCl_3_; 125 MHz) δ ppm: 11.06 (CH_3_), 14.95 (CH_3_), 99.35, 113.23, 119.35, 121.03, 121.05, 128.65, 132.57, 134.45, 135.26, 140.38, 167.85 (C-NH_2_). Anal.Calcd.For C_14_H_15_N_3_S (%): C, 65.34; H, 5.87; N, 16.33 Found (%): C, 65.32; H, 5.88; N, 16.31.

4-(2,6-Dimethyl-1*H*-indol-3-yl)thiazol-2-amine (**5l**). Yield 49%; m.p.155–156 °C (from acetone), ^1^H-NMR (300 MHz, DMSO-d6, ppm) 10.89 (s, 1H, NH), 7.61 (d, *J* = 3.0 Hz, 1H, C4-H), 7.182 (d*, J* = 3.0 Hz, 1H, C7-H), 6.80 (m, 3H, C6-H indole + NH2), 6.26 (s, 1H CH tiazole) 2.62 (s, 3H, 2-CH_3_). ^13^C-NMR (CDCl_3_; 125 MHz) δ ppm: 14.24 (CH_3_), 21.13 (CH_3_), 99.13, 109.36, 111.04, 120.02, 121.67, 125.79, 131.42, 134.44, 135.12, 135.16, 167.38 (C–NH_2_). Anal.Calcd.For C_13_H_13_N_3_S (%): C, 64.17; H, 5.39; N, 17.27. Found (%): C, 64.15; H, 5.43; N, 17.30.

4-(5-Chloro-2-methyl-1*H*-indol-3-yl)thiazol-2-amine (**5m**). Yield 67%; m. 143–145 °C (from isopropanol), ^1^H-NMR (300 MHz, DMSO-d6, ppm) 10.79 (s, 1H, NH), 7.84 (d, *J* = 6.0 Hz, 1H, C4-H), 7.24 (d, *J* = 6.0 Hz, 1H, C7-H), 6.94 (dd, *J* = 12.0 Hz, J = 6.0 Hz, 2H, C5-H, C6-H), 6.61 (s, 2H, NH_2_), 6.28 (s, 1H, CH tiazole), 2.62 (s, 3H, 2-CH_3_). ^13^C-NMR (CDCl_3_; 125 MHz) δ ppm: 14.00 (CH_3_), 99.75, 109.36, 114.75, 121.87, 122.13, 125.43, 129.14, 134.55, 135.12, 135.67, 167.45 (C–NH_2_). Anal.Calcd.For C_12_H_10_ClN_3_S (%): C, 54.65; H, 3.82; Cl, 13.44; N, 15.93. Found (%): C, 54.62; H, 3.84; N, 15.96

4-(5-Ethoxy-1H-indol-3-yl)thiazol-2-amine (**5n**). Yield 56%; mp 210–212 °C (from aqueous acetone), ^1^H-NMR (300 MHz, DMSO-d6, ppm) 10.72 (s, 1H, NH), 7.31 (d, *J* = 6.0 Hz, 1H, C4-H), 7.22 (d, J = 6.0 Hz, 1H, C7-H), 6.96 (s, 1H, NH-CH_3_), 6.94 (dd, *J* = 12.0 Hz, *J* = 6.0 Hz, 2H, C5-H, C6-H), 2.84 (d, 3H, *J* = 6.0Hz, NH-CH_3_ tiazole), 2.48 (s, 3H, 2-CH_3_) 2.22 (s, 3H, C–CH_3_-ttiazole). ^13^C-NMR (CDCl_3_; 125 MHz) δ ppm: 14.05 (CH_3_), 64.00, 107.98, 108.01, 110.56, 110.58, 117.21, 122.43, 127.92, 128.67, 135.74, 153.11, 167.30 (C–NH_2)._ Anal.Calcd.For C_13_H_13_N_3_OS (%): C, 60.21; H, 5.05; N, 16.20. Found (%): C, 60.18; H, 5.07; N, 16.15.

5-Methyl-4-(2-methyl-1*H*-indol-3-yl)thiazol-2-amine (**5o**). Yield 59%; mp.132–133 °C (from acetone), ^1^H-NMR (200 MHz, DMSO-d6, ppm) 10.82 (s, 1H, NH-indole), 7.57 (s, 1H, C2-H), 7.44 (s, 1H, C4-H), 7.21 (d, *J* = 6.0 Hz, 1H, C7-H), 7.19 (s, 1H, NH–CH_3_), 6.72 (d*, J* = 4.0 Hz, 1H, C6-H), 6, 50 (s, 1H, CH tiazole), 3.84 (s, 3H, 5-OCH_3_), 2.98 (s, 3H, NH–CH_3_). ^13^C-NMR (CDCl_3_; 125 MHz) δ ppm: 11.06 (CH_3_), 14.95 (CH_3_), 99.35, 113.23, 119.35, 121.03, 121.05, 128.65, 132.57, 134.45, 135.26, 140.38, 167.85 (C–NH_2_). Anal.Calcd.For C_13_H_13_N_3_S (%): C, 64.17; H, 5.39; N, 17.27. Found (%): C, 64.18; H, 5.34; N, 17.32.

4-(1,2,5-Trimethyl-1*H*-indol-3-yl)thiazol-2-amine (**5p**). Yield 71%; mp 196–197 °C (from ethanol), 1H-NMR (400 MHz, DMSO-d6, ppm) 11.44 (s, 1H, NH-indole), 7.85 (s, 1H, C2-H), 7.81 (m, 1H, C4-H), 7.45 (m, 1H, C5-H), 7.14 (m, 2H, C6-H, C7-H), 7.01 (s, 1H, CH thiazole), 4.99 (s, 1H, –NH–N = C), 2.12 (d, 6H, –C–(CH_3_) _2_). ^13^C-NMR (CDCl_3_; 125 MHz) δ ppm: 10.56 (CH_3_), 21.73 (CH_3_), 30.64 (N-CH_3_), 99.63, 108.02, 109.64, 123.75, 128.11, 128.34, 128.72, 133.62, 135.71, 138.02, 167.32 (C–NH_2_. Anal.Calcd.For C_14_H_15_N_3_S (%): C, 65.34; H, 5.87; N, 16.33. Found (%): C, 65.38; H, 5.83; N, 16.35.

4-(5-Fluoro-2-methyl-1*H*-indol-3-yl)thiazol-2-amine (**5q**). Yield 49%; mp. 155–156 °C (from acetone), ^1^H-NMR (300 MHz, DMSO-d6, ppm) 10,89 (s, 1H, NH), 7.61 (d, *J* = 3.0 Hz, 1H, C4-H), 7.182 (d, *J* = 3.0 Hz, 1H, C7-H), 6,80 (m, 3H, C6-H indole +NH_2_), 6.26 (s, 1H C–H tiazole) 2.62(s, 3H, 2-CH_3_). ^13^C-NMR (CDCl_3_; 125 MHz) δ ppm: 14.36 (CH_3_), 99.65, 109.52, 110.25, 112.49, 116.02, 130.00, 130.85, 134.12, 135.97, 158.11 (C–F), 167.21 (C–NH_2_). Anal.Calcd.For C_12_H_10_FN_3_S (%): C, 58.28; H, 4.08; F, 7.68; N, 16.99. Found (%): C, 58.30; H, 4.05; N, 16.96.

4-(1,2-Dimethyl-1*H*-indol-3-yl)-5-methylthiazol-2-amine (**5r**). Yield 49%; mp.210–212 °C (from acetone), ^1^H-NMR (200 MHz, DMSO-d6, ppm) 7.37 (d, *J* = 4.5 Hz, 2H, C4-H, C5-H,), 7.27 (*d, J* = 3.0 Hz, 1H), 6.99 (dt, *J* = 4.0 Hz, 18.0 Hz, 2H, C7-H, C6-H), 6.39 (s, 2H, NH_2_), 3.74(s, 3H, N–CH_3_) 2.40 (s, 3H, 2-CH_3_), 2,12 (s, 3H, CH_3_ tiazole). ^13^C-NMR (CDCl_3_; 125 MHz) δ ppm: 10.52 (CH_3_), 10.78 (CH_3_), 30.34 (N–CH_3_), 99.65, 109.85, 120.12, 121.14, 121.48, 128.24, 132.67, 136.24, 138.07, 140.53, 167.40 (C–NH_2_). Anal.Calcd.For C_14_H_15_N_3_S (%): C, 65.34; H, 5.87; N, 16.33. Found (%): C, 65.32; H, 5.86; N, 16.38. Anal.Calcd.For C_14_H_15_N_3_S (%): C, 65.34; H, 5.87; N, 16.33. Found (%): C, 65.32; H, 5.86; N, 16.38.

4-(2-Methyl-1H-indol-3-yl)thiazol-2-amine (**5s**). Yield 67%; mp.153–154 °C (from i-propanol), ^1^H-NMR (300 MHz, DMSO-d6, ppm) 10.79 (s, 1H, N-H), 7.84 (d, *J* = 6.0 Hz, 1H, C4-H), 7.24 (d, *J* = 6.0 Hz, 1H, C7-H), 6.94 (dd, *J* = 12.0 Hz, *J* = 6.0 Hz, 2H, C5-H, C^6^-H), 6.61(s, 2H, NH_2_), 6.28(s, 1H, C-H tiazole), 2.62(s, 3H, 2-CH_3_). ^13^C-NMR (CDCl_3_; 125 MHz) δ ppm: 14.29 (CH_3_), 99.62, 109.42, 110.99, 119.68, 121.00, 121.83, 129.03, 134.44, 135.76, 167.30 (C–NH_2_). Anal.Calcd.For C_12_H_11_N_3_S (%): C, 62.86; H, 4.84; N, 18.33. Found (%): C, 62.84; H, 4.82; N, 18.36.

*N*-Methyl-4-(1,2,5-trimethyl-1H-indol-3-yl)thiazol-2-amine (**5t**). Yield 64%; mp 169–171 °C (i-propanol), ^1^H-NMR (200 MHz, DMSO-d6, ppm) 7.60 (s, 1H, C4-H), 7.25 (s, 1H, NH–CH_3_), 7.15(d, *J* = 6.0 Hz, 1H, C7-H), 6.89 (d, *J* = 6.0 Hz, 1H, C6-H), 6.33(s,1H, C-H tiazole), 3.70(s, 3H, N–CH3), 2.97(s, 3H, NH–CH_3_ tiazole), 2.68(s, 3H, 2-CH_3_), 2.43(s, 3H, 5-CH_3_). ^13^C-NMR (CDCl_3_; 125 MHz) δ ppm: 10.61 (CH_3_), 20.74 (CH_3_), 30.74 (CH_3_), 31.32 (N–CH_3_), 99.74, 108.03, 109.61, 123.71, 127.89, 128.31, 128.93, 133.22, 138.21, 135.72, 163.92 (C–NH_2_). Anal.Calcd.For C_15_H_17_N_3_S (%): C, 66.39; H, 6.31; N, 15.48. Found (%): C, 66.35; H, 6.28; N, 15.52.

4-(2-Methyl-1*H*-indol-3-yl)- N,5-dimethyl-thiazol-2-amine (**5u**). Yield 5%6; mp. 184–185 °C (agues acetone), ^1^H-NMR (300 MHz, DMSO-d6, ppm)10.72 (s, 1H, N-H), 7.31 (d, *J* = 6.0 Hz, 1H, C4-H), 7.22 (d, *J* = 6.0 Hz, 1H, C7-H), 6.96 (s, 1H, NH–CH_3_), 6.94 (dd, J = 12.0 Hz, *J* = 6.0 Hz, 2H, C5-H, C6-H), 2.84(d, 3H, *J* = 6.0 Hz, NH–CH_3_ tiazole),2.48(s, 3H, 2-CH_3_) 2,22 (s, 3H,C–CH_3_- tiazole). ^13^C-NMR (CDCl_3_; 125 MHz) δ ppm: 14.78 (CH_3_), 31.56 (NH-CH_3_), 55.82 (O–CH_3_), 99.26, 108.37, 109.82, 112.20, 112.31, 126.43, 126.51, 134.88, 135.22, 154.02 (C–O–CH_3_), 163.43 (C–NHCH_3_). Anal.Calcd.For C_14_H_15_N_3_S (%): C, 65.34; H, 5.87; N, 16.33. Found (%): C, 65.36; H, 5.84; N, 16.32.

4-(5-Methoxy-1H-indol-3-yl)-N-methylthiazol-2-amine (**5v**). Yield 59%; mp.132–133 °C (acetone), ^1^H-NMR (200 MHz, DMSO-d6, ppm) 10.82 (s, 1H, NH-indole), 7.57 (s, 1H, C2-H), 7.44 (s, 1H, C4-H), 7.21 (d, *J* = 6.0 Hz, 1H, C7-H), 7.19 (s, 1H, NH–CH_3_), 6.72 (d, *J* = 4.0 Hz, 1H, C6-H), 6,50 (s, 1H,C–H tiazole), 3,84 (s, 3H, 5-OCH_3_), 2,98 (s, 3H, NH–CH_3_). ^13^C-NMR (CDCl_3_; 125 MHz) δ ppm: 31.25 (NH-CH_3_), 55.88 (O–CH_3_), 108.26, 109.57, 111.13, 112.06, 112.54, 124.63, 128.96, 129.71, 135.74, 154.12 (C–O–CH_3_), 163.29 (C–NHCH_3_). Anal.Calcd.For C_13_H_13_N_3_OS (%): C, 60.21; H, 5.05; N, 16.20. Found (%): C, 60.25; H, 5.04; N, 16.23.

4-(1,2-Dimethyl-1*H*-indol-3-yl)thiazol-2-amine (**5w**). Yield 63%; mp.162–164 °C (i-propanol), ^1^H-NMR (400 MHz, DMSO-d6, ppm) 7.83 (d, *J* = 6.0 Hz, 1H, C4-H), 7.28 (d, *J* = 6.0 Hz, 1H, C7-H), 7.03(m, 2H, C5-H, C^6^-H), 6.48(s, 2H, NH_2_), 6.32(s,1H, C–H tiazole), 3.73 (s, 3H, N–CH_3_), 2.67(s, 3H, 2-CH_3_). ^13^C-NMR (CDCl_3_; 125 MHz) δ ppm: 10.23 (CH_3_), 30.32 (N-CH_3_), 99.15, 109.62, 109.66, 120.04, 121.41, 121.82, 128.25, 136.21, 138.11, 167.32 (C–NH_2_). Anal.Calcd.For C_13_H_13_N_3_S(%): C,,64.17; H,5.39: N, 17.27. Found (%): C, 64.14; H, 5.41; N, 17.30.

4-(1*H*-Indol-3-yl)-2-(2-(propan-2-ylidene)hydrazinyl)thiazole (**5x**). Yield 71%; mp. 233–35 °C (ethanol), ^1^H-NMR (400 MHz, DMSO-d6, ppm) 11.44 (s, 1H, NH-indole), 7.85 (s, 1H, C2-H), 7.81 (m, 1H, C4-H), 7.45 (m, 1H, C5-H), 7.14 (m, 2H, C6-H, C^7^-H), 7.01(s, 1H, C–H tiazole), 4,99 (s, 1H, -NH–N=C), 2.12 (d, 6H, –C–(CH_3_)_2_). ^13^C-NMR (CDCl_3_; 125 MHz) δ ppm: 19.35 (CH_3_), 25.47 (CH_3_), 109.63, 111.00, 111.13, 119.88, 121.83, 122.03, 124.35, 124.91, 135.18, 135.45, 147.50, 170.28 (N–C–(CH_3_)_2_).Anal.Calcd.For C_14_H_14_N_4_S (%): C, 62.20; H, 5.22; N, 20.72. Found (%): C, 62.17; H, 5.24; N, 20.74.

### 3.4. General Procedure for the Synthesis of indol-2-ylthiazoles **6a–f**

To a solution of the corresponding aminothiazole (**5b, 5c, 5w**) (10 mmol) in pyridine (50 mL), acid chloride was added (**7a-b**) (10 mmol). The reaction mass was stirred for 5 h and poured into water (200mL). The precipitate that formed was filtered off, washed thoroughly with water, dried in air, and recrystallized from ethanol. 

*N^1^*-(3,4-Dimethoxyphenethyl)-N^2^-(4-(1,2-dimethyl-1*H*-indol-3-yl)thiazol-2-yl)oxalamide (**6a**). Yield 69%;mp.155–156 °C (from ethanol), ^1^H-NMR (200 MHz, DMSO-d6, ppm) 9.14 (t, 1H, C2-H Ar), 8.01 (d, *J* = 6.0 Hz, 1H, C^5^-H Ar), 7.45 (d, *J* = 6.0 Hz, 1H, C6-H Ar), 7.26 (s, 1H, C-H tiazole), 7.10 (m, 2H, C^4^-H, C7-H indole), 6.83 (s, 2H, NH–CO), 6,79 (m, 2H, C5-H, C6-H indole), 3.73 (s, 6H, OCH_3_), 3.45 (m, 2H, –CH_2_–), 3.33 (s, 3H, N-CH_3_), 2.75 (m, 2H, –CH_2_–), 2,69 (s, 3H, 2-CH_3_). ^13^C-NMR (CDCl_3_; 125 MHz) δ ppm: 19.35 (CH_3_), 25.47 (CH_3_), 109.63, 111.00, 111.13, 119.88, 121.83, 122.03, 124.35, 124.91, 135.18, 135.45, 147.50, 170.28 (N–C–(CH_3_)_2_). Anal.Calcd.For C_25_H_26_N_4_O_4_S (%):C, 62.74; H, 5.48; N, 11.71. Found (%): C, 62.72; H, 5.49; N, 11.70. 

*N*^1^-(4-(1,2-Dimethyl-1*H*-indol-3-yl)thiazol-2-yl)2-N^2^-(2-methoxyethyl)oxalamide (**6b**). Yield 76%; mp.172–173 °C (ethanol), ^1^H-NMR (200 MHz, DMSO-d6, ppm) 12.47 (s, 1H, -NH-C=O), 9.07 (d, *J* = 2.0 Hz, 1H, O=C–NH–CH_2_–), 8.01 d,d, *J* = 6.0 Hz, 1H, C4-H), 7.45 (d, *J* = 6.0 Hz, 1H, C7-H), 7.27 (s, 1H, C–H tiazole), 7.10 (m, 2H, C5-H, C6-H), 3.73 (s, 3H, N–CH_3_), 3.38 (m, 4H, –CH_2_–CH_2_–), 3.33 (s, 3H, CH_3_–O), 2.69 (s, 3H, 2- CH_3_). ^13^C-NMR (CDCl_3_; 125 MHz) δ ppm: 10.72 (CH_3_), 30.23 (N–CH_3_), 35.41, 40.63, 56.81 (O–CH_3_), 56.83 (O-CH_3_), 99.26, 108.53, 109.11, 112.31, 112.84, 120.03, 121.45, 121.67, 122.13, 128.73, 130.24, 135.11, 136.74, 138.06, 148.15, 150.32 (C–O–CH_3_), 161.25 (C=O), 162.47(C=O), 163.18. Anal.Calcd.For C_18_H_20_N_4_O_3_S (%): C, 58.05; H, 5.41; N, 15.04. Found (%): C, 58.02; H, 5.44; N, 15.10. 

3-((4-(1,2-Dimethyl-1*H*-indol-3-yl)thiazol-2-yl)carbamoyl) pyrazine-2-carboxylic acid (**6c**). Yield 75%; mp.304–305 °C (ethanol), ^1^H-NMR (DMSO-d6; 125 MHz) δ ppm: 2.49 (s, 3H, CH_3_), 3.70 (Ss, 3H, N–CH_3_), 4.20 (s, 2H, –CH_2_–), 7.11 (s, 2H, Ar, benz), 7.18–7.50 (m, 2H, Ar), 7.51–7.69 (m, 1H, Ar), 7.76–7.82 (m, 2H, Ar), 8.98 (s, br, 1H, NH–C=O), 11.02 (s, br, 1H, –OH). ^13^C-NMR (CDCl_3_; 125 MHz) δ ppm: 11.61 (CH_3_), 29.59 (N–CH_3_), 39.81, 58.91 (O–CH_3_), 70.36, 107.34, 106,35, 106,84, 119.18, 120.10, 121.30, 126.42, 135.64, 136.66, 146.84, 155.06, 157.14(C=O), 158.27 (C=O). Anal.Calcd.For C_19_H_15_N_5_O_3_S (%): C, 58.01; H, 3.84; N, 17.80. Found (%): C, 58.05; H, 3.82; N, 17.78.

2-(2-((4-(1,2-Dimethyl-1*H*-indol-3-yl)thiazol-2-yl)amino)-2-oxoethyl)benzoic acid (**6d**)**.** Yield 77%; mp.205–206 °C (ethanol), ^1^H-NMR (200 MHz, DMSO-d6, ppm) 12.77 (s, 1H, OH), 12.19 (s, 1H, –NH–C=O), 7,93 (d, *J* = 4.0 Hz, 2H, C4-H, C-H Ar), 7.54 (t, *J* = 10.0 Hz, 1H, C6-H), 7.42 (dd, *J* = 10.0 Hz, *J* = 6.0 Hz, 3H, C5-H, C6-H, C-H Ar), 7.10 (m, 2H, C7-H, C-H Ar), 7.04 (s, 1H, C-H tiazole), 4.23 (s, 2H, –CH_2_–Ar), 3.73 (s, 3H, N–CH_3_), 2.67 (s, 3H, 2-CH_3_). 17 ^13^C-NMR (CDCl_3_; 125 MHz) δ ppm: 10.42 (CH_3_), 29.88 (N–CH_3_), 99.65, 108.93, 109.25, 109.31, 120.12, 121.43, 121.82, 128.74, 135.14, 136.25, 138.03, 142.63, 14377, 143.81, 161.76 (C=O), 162.64, 163.48 (COOH). Anal.Calcd.For C_22_H_19_N_3_O_3_S (%): C, 65.17; H, 4.72; N, 10.36. Found (%): C, 65.15; H, 4.74; N, 10.38.

2-(2-((4-(5-Methoxy-1*H*-indol-3-yl)thiazol-2-yl)amino)-2-oxoethyl)benzoic acid (**6e**). Yield 71%; mp. 245–247 °C (ethanol), ^1^H-NMR (DMSO-d6; 125 MHz) δ ppm: 3.83 (s, 5H, O-CH_3,_ CH_2_), 6.65 (d, 1H, Ar), 6.88 (s, 1H, Ar), 7.22–7.31 (m, 3H, Ar), 7.38–7.46 (m, 2H, Ar), 7.71–7.82 (m, 1H, Ar), 8.67 (s, 1H, =CH–NH), 9.25 (s, br, 1H, NH–C=O), 10.18 (s, br, 1H, NH), 11.09 (s, br, 1H, OH.) ^13^C-NMR (CDCl_3_; 125 MHz) δ ppm: 10.71 (CH_3_), 30.62 (N-CH_3_), 95.22, 109.32, 109.55, 119.02, 121.31, 121.52, 123.82, 128.66, 128.70, 130.11, 131.10, 132.05, 134.82, 135.24, 136.83, 138.02, 162.77, 164.73 (C=O), 167.64 (COOH). Anal.Calcd.For C_21_H_17_N_3_O_4_S (%):C, 61.90; H, 4.21; N, 10.31. Found (%):C, 61.88; H, 4.22; N, 10.34.

2-(2-((4-(6-Methoxy-1*H*-indol-3-yl)thiazol-2-yl)amino)-2-oxoethyl)benzoic acid (**6f**). Yield 69%; 229–230 °C (ethanol), ^1^H-NMR (DMSO-d6; 125 MHz) δ ppm: 3.84 (s, 5H, O–CH_3,_ CH_2_), 6.68 (d, 1H, Ar), 6.90 (s, 1H, Ar), 7.11 (s, 1H, thiaz), 7.18–7.25 (m, 1H, Ar), 7.35–7.40 (m, 2H, Ar), 7.61–7.68 (m, 3H, Ar), 8.62 (s, 1H, =CH-NH), 9.21 (s, br, 1H, NH–C=O), 10.05 (s, br, 1H, NH), 11.08 (s, br, 1H, –OH). ^13^C-NMR (CDCl_3_; 125 MHz) δ ppm: 37.13, 55.82 (O–CH_3_), 95.22, 109.31, 109.66, 111.21, 117.41, 121.34, 124.35, 125.96, 127.54, 130.02, 132.33, 134.42, 135.11, 136.76, 138.39, 156.52 (C–OCH_3_), 162.63, 170.02 (C=O), 172.12 (COOH). Anal.Calcd.For C_21_H_17_N_3_O_4_S (%): C, 61.90; H, 4.21; N, 10.31. Found (%): C, 61.88; H, 4.19; N, 10.37.

### 3.5. Biological Evaluation

#### 3.5.1. Antibacterial Action 

The following Gram-negative bacteria: *Escherichia coli* (ATCC 35210), *Enterobacter cloacae (clinical isolate), Salmonella typhimurium* (ATCC 13311), as well as Gram-positive bacteria*: Listeria monocytogenes* (NCTC 7973), *Bacillus cereus* (clinical isolate), and *Staphylococcus aureus* (ATCC 6538) were used. The bacterial strains were supplied by the Mycological Laboratory, Department of Plant Physiology, Institute for Biological Research” Siniša Stankovic”, Belgrade, Serbia.

The minimum inhibitory and bactericidal (MIC/MBC) concentrations were defined, as described previously [78,79]. Resistant strains used were isolates of *S. aureus*, *E. coli*, and *P.* obtained as reported by Kartsev et al. [78]

#### 3.5.2. Biofilm Formation Inhibition 

Evaluation was performed as described previously [80], with some modifications. The calculation of % inhibition was performed using the following equation:

[(A_620_ control − A_620_ sample)/A_620_ control] × 100 (1)

#### 3.5.3. Checkboard Assay

A checkboard assay was used for the determination of interactions among the selected compounds and antibiotic and streptomycin. The assay was carried out with 96-well microplates containing TSB medium for the resistant *P.aeruginosa* strain, supplemented with examined compounds in concentrations ranging from 1/16 to 4 × MIC, as described previously, [81] in the checkboard manner. The microplates were incubated for 24 h at 37 °C. The MIC of the combinations of examined compounds with streptomycin was determined as for the antimicrobial assay. The fractional inhibitory concentration index (FICI) was calculated by following equation:

FICI = FIC1^0^/MIC1^0^ + FIC2^0^/MIC2^0^ (2)

FIC1^0^ and FIC2^0^ are the MIC values of the combination of tested compounds and antibiotics, and MIC1^0^ and MIC2^0^ represent the MIC values of individual agents. The following cut-offs: FIC ≤ 0.5 synergistic, >0.5 < 2 additive, ≥2 < 4 indifferent, and FIC > 4 antagonistic effects were used for the discussion of obtained results.

#### 3.5.4. Time-Kill Curve Assay

The impact of time on the bactericidal effects of selected compounds was evaluated as described in [82], with some modifications. *P. aeruginosa* cells were incubated with the MBC of compounds with a total volume of 100 µL, which was rubbed into plate-count agar plates with a sterile spreader after 1, 2, 4, and 6 h of treatment. Plates were incubated at 37 °C, and the number of colonies was counted after 24 h.

#### 3.5.5. Antifungal Activity

The strains supplied by Institute for Biological Research ‘‘Siniša Stankovic were: *Aspergillus niger* (ATCC 6275), *Aspergillus fumigatus* (ATCC 1022), *Aspergillus versicolor* (ATCC 11730), *Penicillium funiculosum* (ATCC 36839), *Trichoderma viride* (IAM 5061), and *Penicillium verrucosum var. cyclopium* (food isolate). All experiments were performed in duplicate and repeated three times [83,84]. 

### 3.6. Docking Studies 

Docking simulation was performed using AutoDock 4.2^®so^ software, according to our previous paper [78].

#### 3.6.1. Docking Studies for Prediction of the Mechanism of Antibacterial Activity 

In order to predict the possible mechanism of antibacterial activity of the tested compounds, the enzymes, *E. coli* DNA gyrB, thymidylate kinase, *E. coli* primase, *E. coli* MurB, and DNA topo IV were chosen for docking studies. As the first step, all the co-crystalized original ligands were redocked in the active sites of all enzymes in order to validate the protocol. The RMSD values were in the range of 0.86 to 1.63 Å.

#### 3.6.2. Docking Studies for Prediction of the Mechanism of Antifungal Activity

In order to predict the possible mechanism of antifungal activity of the tested compounds, enzymes CYP51 14α-lanosterol demethylase and dihydrofolate reductase were used. The X-ray crystal structures 5V5Z and 4HOF respectively for each enzyme were obtained for the Protein Data Bank. The docking box was centered on the heme molecule, at the active center of the CYP51 14α-lanosterol demethylase enzyme, both with a target box of 50 × 50 × 50 Å. All selected X-ray crystal structures were in complex with inhibitors. Docking of these inhibitors to their enzyme structures was performed for verification of the method with RMSD values 0.85 and 1.36 Å for CYP51 14α-lanosterol demethylase and dihydrofolate reductase, respectively (Figure S1). Furthermore, the reference drug, ketoconazole, was docked to the active site of 5V5Z structure. 

### 3.7. In-Silico Predictive Studies

Drug-likeness prediction of all compounds was performed as described in our previous paper [85].

### 3.8. Assessment of Cytotoxicity

The growth of MRC-5 cells was previously described [44]. For the assessment of cytotoxicity, the cells were seeded in a 96-well plate at an initial concentration of 5 × 10^4^ cells/mL and allowed to attach for at least 3h before the addition of the compounds at two different concentrations: 1 × 10^−5^ M (10 μΜ) and 1 × 10^−6^ M (1 μΜ). Note that the concentration of DMSO in culture was ≤ 0.2% *v*/*v*, in which no detectable effect on cell proliferation was observed (1). The evaluation of cytotoxicity of each compound and the measure of the number of dead cells was described previously [44,67,68].

## 4. Conclusions

This manuscript reported on the design, synthesis, and in silico and biological evaluation of twenty-nine 4-(indol-3-yl)thiazole-2-amines (**5a–5x**) and 4-indol-3-yl)thiazole acylamines (**6a–6f**) as antimicrobial agents.

The subgroup of indole-based thiazolidinone derivatives (**5a–f, 5i**, **5l–o, 5q, 5s, 5u, 5v, 5x**) showed antibacterial activity, with MIC in the range of 0.06–1.88 mg/mL and MBC of 0.12–3.75 mg/mL. Nevertheless, only one compound, **5x**, exceeded the activity of ampicillin against *S. typhimurium.* The most sensitive bacteria was found to be *S. typhimurium,* while *S. aureus* was the most resistant one The three most active compounds, **5d**, **5m**, and **5x**, appeared to be active against three resistant strains MRSA, *E. coli*, and *P. aeruginosa*, showing better activity against MRSA than both reference drugs. An evaluation of their ability to stop biofilm formation revealed that two compounds (**5m** and **5x**) exhibited stronger inhibition of biofilm formation than both reference drugs in concentration of MIC. Additionally, compound **5m** was more potent against biofilm formation than both reference drugs, even in concentrations of 0.5 MIC.

The determination of the interactions of these selected compounds with antibiotic streptomycin using checkboard assay demonstrated that all compounds were additive with streptomycin, suggesting, based on the in vitro data, that a combination of compounds with this antibiotic can reduce its MIC and subsequently increase its efficiency. Furthermore, the bactericidal nature of three more active compounds, **5d, 5m**, and **5x**, against *P. aeruginosa* was determined by a time-kill curve study. It was found that after 1 h of treatment with compounds **5d**, **5m**, and **5x**, the number of bacterial CFU was reduced by more than 90%, while after 6h, none of the *P. aeruginosa* colonies treated with the selected compounds (**5d, 5m and 5x**) remained viable. 

In the case of methylindole-based thiazolidinones, only 6 out of 14 compounds showed moderate activity; the rest were of very low activity.

According to results of an antifungal activity evaluation, the tested compounds displayed promising potency against all the fungi. In the subgroup of indole-based thiazolidinone derivatives, 7 (**5d, 5e, 5l, 5o, 5u, 5v and 5x**) out of 16 compounds appeared to be more potent than ketoconazole against some fungal species. Compound **5x** demonstrated higher activity than bifonazole against five species, being almost equipotent against *A. fumigatus*, the most resistant strain.

Regarding the second group, methylindole-based thiazolidinone derivatives, all compounds showed promising antifungal activity, with most of them displaying activity almost equipotent to ketoconazole against almost all fungi tested. It should be mentioned that compound **5g** (MIc = 0.06 mg/mL) was more potent than bifonazole (MIC of 0.15 mg/mL), while two compounds (**5r** and **5w**) were equipotent. The most sensitive fungal strain appeared to be *T. viride*, followed by *A. niger*, while *P.v.c* was the most resistant one, followed by *P. funiculosum*.

The diverse sensitivity of bacteria and fungi to the tested compounds demonstrates the dependence of activity on substituents and their position in indole/methylindole rings, as well as on the nature of substituent of the thiazole ring. 

According to docking studies, *E. coli* MurB inhibition is probably responsible for the antibacterial activity of compounds, whereas CYP51 inhibition is implicated in antifungal activity.

All compounds exhibited moderate-to-good drug-likeness scores, ranging from −0.63 to 0.29.

An evaluation of the cytotoxicity of the compounds in normal human MRC-5 cells revealed that the compounds are not toxic.

Finally, compound **5x,** *4*-(1*H*-indol-3-yl)-2-(2-(propan-2-ylidene)hydrazinyl)thiazole, can be considered as lead compound for further development of more potent and safe antibacterial and antifungal agents.

## Figures and Tables

**Table 1:** Antibacterial activity of indole derivatives. (MIC and MBC in mg/mL).

Compounds		*S.a.*	*B.c.*	*L.m.*	*E.c.*	*S.T*	*En.cl.*
**5a**	MIC	0.94 ± 0.00	0.47 ± 0.01	0.23 ± 0.00	0.23 ± 0.00	0.12 ± 0.00	0.23 ± 0.00
	MBC	1.88 ± 0.03	0.94 ± 0.02	0.47 ± 0.01	0.47 ± 0.02	0.23 ± 0.00	0.47 ± 0.03
**5b**	MIC	0.94 ± 0.00	0.47 ± 0.00	0.94 ± 0.00	0.94 ± 0.02	0.47 ± 0.00	0.94 ± 0.02
	MBC	1.88 ± 0.05	0.94 ± 0.02	1.88 ± 0.05	1.88 ± 0.05	0.94 ± 0.02	1.88 ± 0.02
**5c**	MIC	1.88 ± 0.03	0.94 ± 0.02	0.94 ± 0.02	1.88 ± 0.05	0.94 ± 0.02	1.88 ± 0.03
	MBC	3.75 ± 0.08	1.88 ± 0.03	1.88 ± 0.03	3.75 ± 0.05	1.88 ± 0.00	3.75 ± 0.03
**5d**	MIC	0.23 ± 0.00	0.23 ± 0.00	0.23 ± 0.00	0.23 ± 0.00	0.23 ± 0.00	0.23 ± 0.00
	MBC	0.47 ± 0.03	0.47 ± 0.02	0.47 ± 0.02	0.47 ± 0.03	0.47 ± 0.00	0.47 ± 0.03
**5e**	MIC	0.47 ± 0.00	0.12 ± 0.00	0.12 ± 0.00	0.94 ± 0.00	0.23 ± 0.00	0.12 ± 0.00
	MBC	0.94 ± 0.05	0.23 ± 0.00	0.23 ± 0.00	1.88 ± 0.04	0.47 ± 0.00	0.23 ± 0.01
**5f**	MIC	1.88 ± 0.07	0.47 ± 0.01	0.94 ± 0.02	0.47 ± 0.01	0.94 ± 0.03	0.94 ± 0.05
	MBC	3.75 ± 0.06	0.94 ± 0.02	1.88 ± 0.03	0.94 ± 0.03	1.88 ± 0.06	1.88 ± 0.06
**5i**	MIC	3.75 ± 0.06	0.94 ± 0.02	0.94 ± 0.03	0.94 ± 0.03	0.47 ± 0.01	0.94 ± 0.02
	MBC	>3.75	1.88 ± 0.05	1.88 ± 0.05	1.88 ± 0.05	0.94 ± 0.02	1.88 ± 0.03
**5l**	MIC	1.88 ± 0.07	0.94 ± 0.02	0.94 ± 0.02	1.88 ± 0.05	0.94 ± 0.02	0.94 ± 0.03
	MBC	3.75 ± 0.08	1.88 ± 0.03	1.88 ± 0.05	3.75 ± 0.07	1.88 ± 0.05	1.88 ± 0.05
**5m**	MIC	0.12 ± 0.00	0.06 ± 0.00	0.12 ± 0.00	0.23 ± 0.00	0.23 ± 0.00	0.23 ± 0.00
	MBC	0.23 ± 0.00	0.12 ± 0.00	0.23 ± 0.00	0.47 ± 0.00	0.47 ± 0.00	0.47 ± 0.00
**5n**	MIC	1.88 ± 0.06	0.94 ± 0.02	0.94 ± 0.02	1.88 ± 0.05	0.94 ± 0.02	1.88 ± 0.02
	MBC	3.75 ± 0.08	1.88 ± 0.06	1.88 ± 0.05	3.75 ± 0.08	1.88 ± 0.08	3.75 ± 0.06
**5o**	MIC	1.88 ± 0.06	0.94 ± 0.02	0.94 ± 0.02	1.88 ± 0.05	1.88 ± 0.05	0.94 ± 0.02
	MBC	3.75 ± 0.08	1.88 ± 0.06	1.88 ± 0.06	3.75 ± 0.10	3.75 ± 0.08	1.88 ± 0.05
**5q**	MIC	0.23 ± 0.00	0.23 ± 0.00	0.23 ± 0.00	1.88 ± 0.05	0.47 ± 0.02	0.23 ± 0.01
	MBC	0.47 ± 0.03	0.47 ± 0.02	0.47 ± 0.02	3.75 ± 0.08	0.94 ± 0.02	0.47 ± 0.02
**5s**	MIC	0.94 ± 0.05	0.47 ± 0.02	0.23 ± 0.01	0.94 ± 0.03	0.94 ± 0.03	0.47 ± 0.02
	MBC	1.88 ± 0.07	0.94 ± 0.02	0.47 ± 0.02	1.88 ± 0.05	1.88 ± 0.06	0.94 ± 0.03
**5u**	MIC	1.88 ± 0.06	0.94 ± 0.01	0.47 ± 0.02	3.75 ± 0.08	1.88 ± 0.06	0.94 ± 0.03
	MBC	3.75 ± 0.08	1.88 ± 0.03	0.94 ± 0.03	>3.75	3.75 ± 0.10	1.88 ± 0.05
**5v**	MIC	1.88 ± 0.06	0.47 ± 0.02	0.47 ± 0.02	0.94 ± 0.03	0.47 ± 0.02	0.94 ± 0.03
	MBC	3.75 ± 0.08	0.94 ± 0.02	0.94 ± 0.05	1.88 ± 0.05	0.94 ± 0.04	1.88 ± 0.06
**5x**	MIC	0.12 ± 0.00	0.12 ± 0.00	0.12 ± 0.00	0.12 ± 0.00	0.06 ± 0.00	0.12 ± 0.00
	MBC	0.23 ± 0.00	0.23 ± 0.00	0.23 ± 0.00	0.23 ± 0.00	0.12 ± 0.00	0.23 ± 0.00
**Streptomycin**	MIC	0.10 ± 0.00	0.02 ± 0.00	0.15 ± 0.00	0.10 ± 0.00	0.10 ± 0.00	0.02 ± 0.00
	MBC	0.20 ± 0.01	0.05 ± 0.00	0.30 ± 0.01	0.20 ± 0.00	0.20 ± 0.01	0.05 ± 0.00
**Ampicillin**	MIC	0.10 ± 0.00	0.10 ± 0.00	0.15 ± 0.00	0.15 ± 0.00	0.10 ± 0.00	0.10 ± 0.00
	MBC	0.15 ± 0.00	0.15 ± 0.00	0.30 ± 0.02	0.20 ± 0.01	0.20 ± 0.00	0.15 ± 0.01

**Table 2:** FICI indexes of combinations of selected compounds with streptomycin.

Compound	FICI
**5d**	1.5
**5m**	1.5
**5x**	1.5

**Table 3:** Antibacterial activity against resistant strains (MIC/MBC in mg/mL) and inhibition of biofilm formation (%).

Compounds		*MRSA*	*P. a*	*E. c.*	MIC	0.5 MIC
**5d**	MIC	0.94 ± 0.00	0.23 ± 0.00	1.88 ± 0.06	39.38 ± 9.25	20.62 ± 3.22
	MBC	1.88 ± 0.06	0.47 ± 0.01	3.75 ± 0.00		
**5m**	MIC	0.23 ± 0.00	0.94 ± 0.00	0.47 ± 0.01	80.30 ± 5.62	69.55 ± 11.45
	MBC	0.47 ± 0.01	1.88 ± 0.06	0.94 ± 0.00		
**5x**	MIC	0.47 ± 0.01	0.47 ± 0.01	0.47 ± 0.01	75.52 ± 11.99	21.19 ± 3.50
	MBC	0.94 ± 0.00	0.94 ± 0.00	0.94 ± 0.00		
**Streptomycin**	MIC	0.10 ± 0.00	0.05 ± 0.00	0.10 ± 0.00	63.56 ± 8.28	29.12 ± 1.22
	MBC	/	0.10 ± 0.00	0.20 ± 0.01		
**Ampicillin**	MIC	/	0.20 ± 0.01	0.20 ± 0.01	70.00 ± 10.23	52.36 ± 3.67
	MBC	/	/	/		

**Table 4:** Antifungal activity of indole derivatives. (MIC and MBC in mg/mL).

Compounds		*A.f.*	*A.n.*	*A.v.*	*P.f.*	*T.v.*	*P.v.c.*
**5a**	MIC	0.47 ± 0.00	0.47 ± 0.00	0.23 ± 0.00	0.47 ± 0.00	0.23 ± 0.00	0.23 ± 0.00
	MFC	0.94 ± 0.03	0.94 ± 0.03	0.47 ± 0.00	0.94 ± 0.07	0.47 ± 0.02	0.47 ± 0.02
**5b**	MIC	1.88 ± 0.08	0.23 ± 0.01	0.47 ± 0.00	0.47 ± 0.02	0.23 ± 0.01	0.47 ± 0.03
	MFC	3.75 ± 0.10	0.47 ± 0.03	0.94 ± 0.05	0.94 ± 0.05	0.47 ± 0.03	0.94 ± 0.07
**5c**	MIC	1.88 ± 0.08	0.47 ± 0.03	0.47 ± 0.02	0.47 ± 0.03	0.23 ± 0.01	0.12 ± 0.00
	MFC	3.75 ± 0.06	0.94 ± 0.07	0.94 ± 0.07	0.94 ± 0.07	0.47 ± 0.02	0.23 ± 0.01
**5d**	MIC	0.23 ± 0.00	0.06 ± 0.00	0.23 ± 0.00	0.47 ± 0.03	0.06 ± 0.00	0.47 ± 0.02
	MFC	0.47 ± 0.01	0.12 ± 0.00	0.47 ± 0.03	0.94 ± 0.06	0.12 ± 0.00	0.94 ± 0.04
**5e**	MIC	0.47 ± 0.02	0.12 ± 0.00	0.23 ± 0.02	0.47 ± 0.02	0.23 ± 0.00	0.47 ± 0.02
	MFC	0.94 ± 0.05	0.23 ± 0.01	0.47 ± 0.02	0.94 ± 0.08	0.47 ± 0.02	0.94 ± 0.06
**5f**	MIC	0.94 ± 0.05	0.47 ± 0.03	0.47 ± 0.02	0.94 ± 0.06	0.23 ± 0.00	0.12 ± 0.00
	MFC	1.88 ± 0.08	0.94 ± 0.07	0.94 ± 0.06	1.88 ± 0.08	0.47 ± 0.03	0.23 ± 0.01
**5i**	MIC	0.47 ± 0.01	0.23 ± 0.01	0.23 ± 0.01	0.94 ± 0.03	0.06 ± 0.00	0.94 ± 0.02
	MFC	0.94 ± 0.02	0.47 ± 0.03	0.47 ± 0.03	1.88 ± 0.08	0.12 ± 0.00	1.88 ± 0.05
**5l**	MIC	0.23 ± 0.00	0.06 ± 0.00	0.06 ± 0.00	0.47 ± 0.03	0.12 ± 0.00	0.12 ± 0.00
	MFC	0.47 ± 0.03	0.12 ± 0.00	0.12 ± 0.00	0.94 ± 0.04	0.23 ± 0.00	0.23 ± 0.00
**5m**	MIC	0.47 ± 0.02	0.23 ± 0.00	0.23 ± 0.00	0.47 ± 0.02	0.23 ± 0.00	0.47 ± 0.03
	MFC	0.94 ± 0.03	0.47 ± 0.02	0.47 ± 0.03	0.94 ± 0.07	0.47 ± 0.01	0.94 ± 0.07
**5n**	MIC	0.47 ± 0.01	0.23 ± 0.01	0.47 ± 0.03	0.47 ± 0.02	0.12 ± 0.00	0.12 ± 0.00
	MFC	0.94 ± 0.04	0.47 ± 0.03	0.94 ± 0.07	0.94 ± 0.06	0.23 ± 0.01	0.23 ± 0.02
**5o**	MIC	0.23 ± 0.01	0.23 ± 0.01	0.23 ± 0.01	0.47 ± 0.03	0.23 ± 0.02	0.47 ± 0.02
	MFC	0.47 ± 0.03	0.47 ± 0.03	0.47 ± 0.03	0.94 ± 0.05	0.47 ± 0.03	0.94 ± 0.05
**5q**	MIC	0.23 ± 0.00	0.23 ± 0.00	0.47 ± 0.02	0.47 ± 0.03	0.23 ± 0.00	0.47 ± 0.01
	MFC	0.47 ± 0.03	0.47 ± 0.03	0.94 ± 0.05	0.94 ± 0.05	0.47 ± 0.03	0.94 ± 0.03
**5s**	MIC	0.47 ± 0.02	0.47 ± 0.01	0.47 ± 0.02	0.47 ± 0.03	0.23 ± 0.01	0.47 ± 0.03
	MFC	0.94 ± 0.05	0.94 ± 0.03	0.94 ± 0.03	0.94 ± 0.03	0.47 ± 0.02	0.94 ± 0.07
**5u**	MIC	0.94 ± 0.05	0.12 ± 0.00	0.23 ± 0.00	0.47 ± 0.03	0.23 ± 0.00	0.47 ± 0.03
	MFC	1.88 ± 0.06	0.23 ± 0.00	0.47 ± 0.01	0.94 ± 0.05	0.47 ± 0.02	0.94 ± 0.05
**5v**	MIC	0.23 ± 0.01	0.06 ± 0.00	0.06 ± 0.00	0.47 ± 0.02	0.06 ± 0.00	0.94 ± 0.04
	MFC	0.47 ± 0.03	0.12 ± 0.00	0.12 ± 0.01	0.94 ± 0.03	0.12 ± 0.00	1.88 ± 0.07
**5x**	MIC	0.12 ± 0.00	0.06 ± 0.00	0.06 ± 0.00	0.12 ± 0.00	0.06 ± 0.00	0.06 ± 0.00
	MFC	0.23 ± 0.01	0.12 ± 0.01	0.12 ± 0.00	0.23 ± 0.01	0.12 ± 0.01	0.12 ± 0.01
**Bifonazole**	MIC	0.15 ± 0.00	0.15 ± 0.00	0.10 ± 0.00	0.20 ± 0.01	0.15 ± 0.00	0.10 ± 0.00
	MFC	0.20 ± 0.00	0.20 ± 0.00	0.20 ± 0.01	0.25 ± 0.01	0.20 ± 0.01	0.20 ± 0.00
**Ketoconazole**	MIC	0.20 ± 0.01	0.20 ± 0.01	0.20 ± 0.00	0.20 ± 0.00	1.00 ± 0.01	0.20 ± 0.01
	MFC	0.50 ± 0.03	0.50 ± 0.03	0.50 ± 0.02	0.50 ± 0.03	1.50 ± 0.02	0.30 ± 0.01

**Table 5:** Antifungal activity of methylindole derivatives (MIC and MBC in mg/mL).

Compounds		*A.f.*	*A.n.*	*A.v.*	*P.f.*	*T.v.*	*P.v.c.*
**5g**	MIC	0.47 ± 0.03	0.06 ± 0.00	0.23 ± 0.01	0.47 ± 0.02	0.23 ± 0.01	0.94 ± 0.02
	MFC	0.94 ± 0.05	0.12 ± 0.01	0.47 ± 0.01	0.94 ± 0.03	0.47 ± 0.01	1.88 ± 0.05
**5h**	MIC	1.88 ± 0.08	0.47 ± 0.01	0.47 ± 0.01	0.23 ± 0.01	0.23 ± 0.01	0.47 ± 0.02
	MFC	3.75 ± 0.08	0.94 ± 0.03	0.94 ± 0.03	0.47 ± 0.02	0.47 ± 0.02	0.94 ± 0.03
**5j**	MIC	0.47 ± 0.02	0.23 ± 0.01	0.12 ± 0.01	0.47 ± 0.01	0.23 ± 0.01	0.94 ± 0.05
	MFC	0.94 ± 0.04	0.47 ± 0.03	0.23 ± 0.01	0.94 ± 0.03	0.47 ± 0.02	1.88 ± 0.08
**5k**	MIC	0.94 ± 0.03	0.47 ± 0.01	0.47 ± 0.02	0.47 ± 0.02	0.23 ± 0.01	0.47 ± 0.02
	MFC	1.88 ± 0.08	0.94 ± 0.03	0.94 ± 0.03	0.94 ± 0.05	0.47 ± 0.03	0.94 ± 0.02
**5p**	MIC	0.94 ± 0.03	0.47 ± 0.03	0.47 ± 0.02	0.23 ± 0.01	0.23 ± 0.01	0.94 ± 0.03
	MFC	1.88 ± 0.04	0.94 ± 0.03	0.94 ± 0.03	0.47 ± 0.02	0.47 ± 0.02	1.88 ± 0.04
**5r**	MIC	0.23 ± 0.01	0.12 ± 0.00	0.23 ± 0.01	0.23 ± 0.01	0.23 ± 0.00	0.47 ± 0.03
	MFC	0.47 ± 0.02	0.23 ± 0.01	0.47 ± 0.02	0.47 ± 0.02	0.47 ± 0.02	0.94 ± 0.04
**5t**	MIC	0.23 ± 0.01	0.23 ± 0.01	0.47 ± 0.02	0.47 ± 0.02	0.23 ± 0.01	0.47 ± 0.02
	MFC	0.47 ± 0.02	0.47 ± 0.02	0.94 ± 0.02	0.94 ± 0.03	0.47 ± 0.02	0.94 ± 0.02
**5w**	MIC	0.23 ± 0.01	0.12 ± 0.00	0.23 ± 0.01	1.88 ± 0.03	0.23 ± 0.01	1.88 ± 0.05
	MFC	0.47 ± 0.02	0.23 ± 0.01	0.47 ± 0.03	3.75 ± 0.09	0.47 ± 0.02	3.75 ± 0.06
**6a**	MIC	0.47 ± 0.02	0.23 ± 0.01	0.23 ± 0.01	0.94 ± 0.05	0.47 ± 0.02	0.94 ± 0.05
	MFC	0.94 ± 0.03	0.47 ± 0.02	0.47 ± 0.03	1.88 ± 0.06	0.94 ± 0.02	1.88 ± 0.08
**6b**	MIC	0.23 ± 0.00	0.23 ± 0.01	0.47 ± 0.02	0.47 ± 0.02	0.23 ± 0.01	0.47 ± 0.01
	MFC	0.47 ± 0.02	0.47 ± 0.02	0.94 ± 0.03	0.94 ± 0.05	0.47 ± 0.03	0.94 ± 0.05
**6c**	MIC	0.47 ± 0.02	0.23 ± 0.01	0.23 ± 0.01	0.47 ± 0.02	0.23 ± 0.01	0.94 ± 0.01
	MFC	0.94 ± 0.04	0.47 ± 0.03	0.47 ± 0.02	0.94 ± 0.03	0.47 ± 0.02	1.88 ± 0.06
**6d**	MIC	0.23 ± 0.02	0.23 ± 0.01	0.47 ± 0.02	0.23 ± 0.01	0.23 ± 0.01	1.88 ± 0.05
	MFC	0.47 ± 0.03	0.47 ± 0.03	0.94 ± 0.03	0.47 ± 0.02	0.47 ± 0.02	3.75 ± 0.08
**6e**	MIC	3.75 ± 0.00	3.75 ± 0.00	3.75 ± 0.00	3.75 ± 0.00	3.75 ± 0.00	3.75 ± 0.00
	MFC	>3.75	>3.75	>3.75	>3.75	>3.75	>3.75
**6f**	MIC	0.47 ± 0.01	0.23 ± 0.01	0.47 ± 0.02	0.47 ± 0.01	0.23 ± 0.01	0.23 ± 0.02
	MFC	0.94 ± 0.03	0.47 ± 0.02	0.94 ± 0.02	0.94 ± 0.03	0.47 ± 0.02	0.47 ± 0.02
**Bifonazole**	MIC	0.15 ± 0.00	0.15 ± 0.00	0.10 ± 0.00	0.20 ± 0.01	0.15 ± 0.00	0.10 ± 0.00
	MFC	0.20 ± 0.00	0.20 ± 0.00	0.20 ± 0.01	0.25 ± 0.01	0.20 ± 0.01	0.20 ± 0.00
**Ketoconazole**	MIC	0.20 ± 0.01	0.20 ± 0.01	0.20 ± 0.00	0.20 ± 0.00	1.00 ± 0.01	0.20 ± 0.01
	MFC	0.50 ± 0.03	0.50 ± 0.03	0.50 ± 0.02	0.50 ± 0.03	1.50 ± 0.02	0.30 ± 0.01

**Table 6:** Molecular docking free binding energies (kcal/mol) with antibacterial targets of indole-based thiazole derivatives.

No	Est. Binding Energy (kcal/mol)					I-H	Residues Involved in Hydrogen Bond Formation
	DNA topo IV PDB ID: 1S16	*E. coli* Primase PDB ID: 1DDE	Gyrase PDB ID: 1KZN	Thymidylate Kinase PDB ID: 4QGG	*E. coli* MurB PDB ID: 2Q85		
5a	−2.56	−3.46	−5.88	−	−9.88	2	Arg158, Ser228
5c	-	−1.52	−4.12	−1.57	−8.15	1	Ser228
5d	−4.33	−2.92	−5.73	−	−10.13	2	Ser228
5e	−3.72	−1.55	−6.77	−1.28	−9.73	2	Tyr189, Ser228
5f	-	-	−5.24	-	−8.85	2	Asn50, Arg158
5i	-	−1.23	−5.37	-	−6.94	1	Arg158
5l	−2.46	−	−4.31	-	−8.91	2	Asn50, Ser228
5m	−3.51	−2.75	−5.37	−1.67	−9.89	2	Arg158, Ser228
5n	-	-	−4.88	−	−8.06	1	Ser228
5o	-	−1.74	−5.39	−	−8.17	1	Ser228
5q	−4.90	-	−6.15	−1.37	−9.31	2	Arg158, Ser228
5s	−3.69	−2.48	−5.82	−2.63	−8.95	2	Ser228, Lys261
5u	-	-	−5.78	-	−7.02	1	Asn50
5v	−3.11	−1.67	−4.95	-	−8.73	2	Arg158, Tyr189
5x	−4.85	−3.97	−6.28	-	−10.74	3	Asn50, Ser228, Glu324

## Data Availability

Data sharing contains in this article.

## Supplementary Materials

The following are available online at https://www.mdpi.com/article/10.3390/ph14111096/s1. Copies of ^1^H and ^13^C NMR spectra for all new synthesized compounds have been submitted along with the manuscript, Table S1, and Figure S1.
