# Supplementary material for: 4-(Indol-3-yl)thiazole-2-amines and 4-ιndol-3-yl)thiazole Acylamines as Νovel Antimicrobial Agents: Synthesis, In Silico and In Vitro Evaluation
Source: Pharmaceuticals (Basel). 2021 Oct 28;14(11):1096. doi: 10.3390/ph14111096 (PMC8624152; doi:10.3390/ph14111096)
Supplement: Supplementary file 1 [file pharmaceuticals-14-01096-s001.zip › Figure S1.pdf]

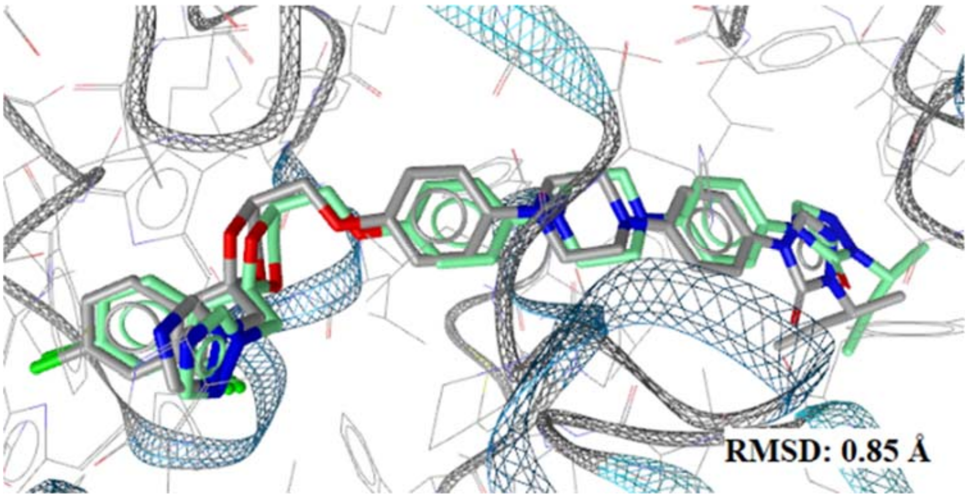

Figure S1. Docking of the initial inhibitor 2-[(2R)-butan-2-yl]-4-{4-[4-(4- {[(2R,4S)-2-(2,4-dichlorophenyl)-2-(1H-1,2,4-triazol-1-ylmethyl)-1,3-dioxolan-4-yl]methoxy}phenyl)piperazin-1-yl]phenyl}-2,4-dihydro-3H-1,2,4-triazol-3-one to the 5V5Z structure.
