# Supplementary material for: 4-(Indol-3-yl)thiazole-2-amines and 4-ιndol-3-yl)thiazole Acylamines as Νovel Antimicrobial Agents: Synthesis, In Silico and In Vitro Evaluation
Source: Pharmaceuticals (Basel). 2021 Oct 28;14(11):1096. doi: 10.3390/ph14111096 (PMC8624152; doi:10.3390/ph14111096)
Supplement: Supplementary file 1 [file pharmaceuticals-14-01096-s001.zip › SI files/supplementary.pdf]

# 4-(Indol-3-yl) thiazole-2-amines and 4-ındol-3-yl)thiazole acylamines as Novel antimicrobial agents. Synthesis, in silico and in vitro evaluation.

Simakov S.[a], Kartsev V.[b], Petrou A.[c], Nicolaou I.[c] Geronikaki A.[c,]*, Ivanov M.[d], Kostić M.[d], Glamočlija J.[d], Soković M.[d], Talea T.D.[e] and Vizirianakis I.[e, f]

[a] *Belgorod State University, Russia.*
[b] *InterBioScreen, Chernogolovka, Moscow Region, Russia.*
[c] *School of Pharmacy Dep/t Pharm. Chemistry, Aristotle University of Thessaloniki, 54124, Thessaloniki, Greece.*
[d]*Mycological Laboratory, Department of Plant Physiology, Institute for Biological Research "Siniša Stanković", National Institute of Republic of Serbia, University of Belgrade, Beograd 11060, Serbia.*
[e] *Laboratory of Pharmacology, School of Pharmacy, Aristotle University of Thessaloniki, GR-54124 Thessaloniki, Greece.*
[f] *Department of Life and Health Sciences, University of Nicosia, CY-1700 Nicosia, Cyprus.*

**Table S1**. Antibacterial activity of methylindole derivatives. (MIC and MBC in mg/ml)

| Compounds | | *S.a.* | *B.c.* | *L.m.* | *E.c.* | *S.t.* | *En.cl.* |
|---|---|---|---|---|---|---|---|
| **5g** | **MIC** | >3.75 | 0.47 | 0.94 | 0.47 | 0.94 | 0.94 |
| | **MBC** | >3.75 | 0.94 | 1.88 | 0.94 | 1.88 | 1.88 |
| **5h** | **MIC** | >3.75 | 0.94 | 0.94 | 1.88 | 0.94 | 0.94 |
| | **MBC** | >3.75 | 1.88 | 1.88 | 3.75 | 1.88 | 1.88 |
| **5i** | **MIC** | >3.75 | 0.94 | 0.94 | 0.94 | 0.47 | 0.94 |
| | **MBC** | >3.75 | 1.88 | 1.88 | 1.88 | 0.94 | 1.88 |
| **5j** | **MIC** | >3.75 | 0.94 | 0.94 | 1.88 | 0.94 | 1.88 |
| | **MBC** | >3.75 | 1.88 | 1.88 | 3.75 | 1.88 | 3.75 |
| **5k** | **MIC** | >3.75 | 1.88 | 1.88 | 1.88 | 1.88 | >3.75 |
| | **MBC** | >3.75 | 3.75 | 3.75 | 3.75 | 3.75 | >3.75 |
| **5p** | **MIC** | >3.75 | 0.94 | 0.47 | 3.75 | 3.75 | 3.75 |
| | **MBC** | >3.75 | 1.88 | 0.94 | >3.75 | >3.75 | >3.75 |
| **5t** | **MIC** | >3.75 | >3.75 | 0.47 | >3.75 | >3.75 | >3.75 |
| | **MBC** | >3.75 | >3.75 | 0.94 | >3.75 | >3.75 | >3.75 |
| **5w** | **MIC** | >3.75 | 0.94 | 1.88 | 1.88 | >3.75 | >3.75 |
| | **MBC** | >3.75 | 1.88 | 3.75 | 3.75 | >3.75 | >3.75 |
| **6a** | **MIC** | >3.75 | >3.75 | 0.94 | >3.75 | >3.75 | >3.75 |
| | **MBC** | >3.75 | >3.75 | 1.88 | >3.75 | >3.75 | >3.75 |
| **6b** | **MIC** | >3.75 | >3.75 | 1.88 | >3.75 | >3.75 | >3.75 |
| | **MBC** | >3.75 | >3.75 | 3.75 | >3.75 | >3.75 | >3.75 |
| **6c** | **MIC** | >3.75 | >3.75 | >3.75 | >3.75 | >3.75 | >3.75 |
| | **MBC** | >3.75 | >3.75 | >3.75 | >3.75 | >3.75 | >3.75 |
| **6d** | **MIC** | >3.75 | 1.88 | 0.94 | >3.75 | >3.75 | >3.75 |
| | **MBC** | >3.75 | 3.75 | 1.88 | >3.75 | >3.75 | >3.75 |
| **6e** | **MIC** | >3.75 | >3.75 | 1.88 | >3.75 | >3.75 | >3.75 |
| | **MBC** | >3.75 | >3.75 | 3.75 | >3.75 | >3.75 | >3.75 |
| **6f** | **MIC** | >3.75 | >3.75 | 1.88 | >3.75 | >3.75 | >3.75 |
| | **MBC** | >3.75 | >3.75 | 3.75 | >3.75 | >3.75 | >3.75 |
| **Streptomycin** | **MIC** | 0.10±0.00 | 0.02±0.00 | 0.15±0.00 | 0.10±0.00 | 0.10±0.00 | 0.02±0.00 |
| | **MBC** | 0.20±0.01 | 0.05±0.00 | 0.30±0.01 | 0.20±0.00 | 0.20±0.01 | 0.05±0.00 |
| **Ampicillin** | **MIC** | 0.10±0.00 | 0.10±0.00 | 0.15±0.00 | 0.15±0.00 | 0.10±0.00 | 0.10±0.00 |
| | **MBC** | 0.15±0.00 | 0.15±0.00 | 0.30±0.02 | 0.20±0.01 | 0.20±0.00 | 0.15±0.01 |

**Table S2** Molecular docking free binding energies (kcal/mol) to antifungal targets of indole-based thiazole derivatives.

| No | Est. binding energy(kcal/mol) | | I-H | Residues involved in Hydrogen Bonds | Aromatic-positive ionizable | Hydrophobic interactions | Interactions with HEM601 |
|---|---|---|---|---|---|---|---|
| | Dihydrofolate reductase | $CYP51_{Ca}$ | | | | | |

| | (PDB: 4HOF) | (PDB: 5V5Z) | | | | interactions | of CYP51$_{Ca}$ |
|---|---|---|---|---|---|---|---|
| **5a** | -1.25 | -8.35 | 1 | Tyr64 | - | Hem601, Ile131, Leu376, Met508 | Hydrophobic |
| **5c** | -2.15 | -7.96 | 1 | Tyr118 | - | Hem601, Tyr64, Tyr118, Ile131, Leu376, Met508 | Hydrophobic |
| **5d** | -3.69 | -11.62 | 1 | Tyr132 | Hem601 | Hem601, Ile131, Tyr132, Phe288, Thr311, Leu376, Met508, Val509 | **Aromatic**<br>**Fe binding**<br>Hydrophobic |
| **5e** | -3.45 | -8.83 | 2 | Tyr64, Tyr118 | - | Hem601, Ile131, Leu300, Leu376, Met508 | Hydrophobic |
| **5f** | -2.67 | -8.05 | 1 | Tyr132 | - | Hem601, Tyr64, Ile131, Tyr132, His377, Met508 | Hydrophobic |
| **5i** | -1.14 | -8.13 | 1 | Tyr118 | - | Hem601, Tyr118, Ile131, Leu376, Met508 | Hydrophobic |
| **5l** | -4.17 | -11.13 | 2 | Tyr132, Met508 | Tyr188, Hem601 | Hem601, Tyr118, Tyr122, Ile131, Tyr132, Phe233, Tyr305, Leu376, Met508, Val509 | Aromatic<br>Hydrophobic |
| **5m** | -3.67 | -10.41 | 2 | Tyr64, Tyr132 | - | Hem601, Tyr118, Tyr132, Leu376, Met508, Val509 | Hydrophobic |
| **5n** | -1.11 | -9.46 | 2 | Tyr118, Met508 | Tyr132 | Hem601, Ile131, Ile304, Leu376, Met508, Val509 | Hydrophobic |
| **5o** | -2.03 | -9.54 | 2 | Tyr64, Tyr118 | Tyr132 | Hem601, Tyr118, Ile131, Leu376, Met508, Val509 | Hydrophobic |
| **5q** | -1.18 | -8.21 | 1 | Tyr118 | Tyr188 | Hem601, Ile131, Tyr132, Leu376 | Hydrophobic |
| **5s** | -3.97 | -7.38 | 1 | Tyr64 | - | Hem601, Ile131, Tyr132, Met508 | Hydrophobic |
| **5u** | -1.29 | -8.21 | 1 | Met508 | Hem601 | Hem601, Ile131, Tyr132, Leu376, Met508, Val509 | **Aromatic**<br>Hydrophobic |
| **5v** | -3.12 | -9.75 | 1 | Tyr64 | Hem601 | Hem601, Tyr118, Ile131, Tyr132, Leu376, Met508, Val509 | **Aromatic**<br>Hydrophobic |
| **5x** | -3.74 | -12.55 | 1 | Hem601 | - | Hem601, Tyr118, Thr122, Phe126, Ile131, Tyr132, Phe288, Phe233, Thr311, Leu376, Phe380, Met508, Val509 | **Hydrogen bond**<br>Hydrophobic |
| **Ketoconazole** | - | -8.23 | 1 | Tyr64 | Hem601 | Tyr118, Ile131, Tyr132, Hem601, Ile304, Leu300, Leu376, Met508 | **Aromatic**, Hydrophobic |

*Number of hydrogen bonds.

**Table S3 .** Molecular docking free binding energies (kcal/mol) to antifungal targets of methylindole-based thiazole derivatives.

| No | Est. binding energy(kcal/mol) | I-H* | Residues involved in | Aromatic-positive | Hydrophobic interactions | Interactions with |
|---|---|---|---|---|---|---|

| | Dihydrofolate reductase (PDB: 4HOF) | $CYP51_{Ca}$ (PDB: 5V5Z) | | Hydrogen Bonds | ionizable interactions | | HEM601 of $CYP51_{Ca}$ |
|---|---|---|---|---|---|---|---|
| **5b** | -3.26 | -10.84 | 2 | Tyr64, Tyr132 | Tyr188 | Hem601, Tyr118, Ile131, Tyr132, Leu376, Met508 | Hydrophobic |
| **5g** | -3.14 | -10.54 | 1 | Hem601 | - | Hem601, Phe126, Ile131, Tyr132, Phe233, Thr311, Leu376, Met508 | **Hydrogen bond,** Hydrophobic |
| **5h** | -1.88 | -8.15 | - | - | - | Hem601, Phe126, Ile131, Leu376, Met508 | Hydrophobic |
| **5j** | -3.16 | -9.70 | 1 | Tyr132 | Hem601, Tyr188 | Hem601, Tyr118, Ile131, Tyr132, Leu376, Met508 | **Aromatic**, Hydrophobic |
| **5k** | -2.11 | -9.41 | 1 | Tyr64 | - | Hem601, Tyr118, Ile131, Leu376, Met508 | Hydrophobic |
| **5p** | -3.65 | -8.76 | 1 | Tyr132 | Hem601 | Hem601, Ile131, Tyr132, Thr311, Leu376, Met508 | **Aromatic,** Hydrophobic |
| **5r** | -3.38 | -11.15 | - | - | Hem601 | Hem601, Tyr118, Thr122, Ile131, Tyr132, Thr311, Leu376, Met508 | **Aromatic,** **Fe binding,** Hydrophobic |
| **5t** | -3.47 | -11.28 | 1 | Hem601 | Hem601 | Hem601, Tyr118, Phe126, Ile131, Tyr132, Thr311, Leu376 | **Hydrogen bond,** **Aromatic,** **Fe binding,** Hydrophobic |
| **5w** | -2.74 | -7.82 | - | - | - | Hem601, Tyr122, Phe126, Leu376, Met508 | Hydrophobic |
| **6b** | -1.27 | -8.73 | 1 | Tyr64 | - | Hem601, Tyr118, Ile131, Leu376, Met508 | Hydrophobic |
| **6c** | -2.55 | -11.02 | 1 | Hem601 | Tyr188, Hem601 | Hem601, Tyr118, Tyr122, Ile131, Tyr132, Phe233, Leu376, Met508 | **Hydrogen bond,** **Aromatic,** Hydrophobic |
| **6d** | -2.97 | -9.72 | 2 | Tyr64, Tyr132 | Hem601 | Hem601, Tyr118, Tyr132, Leu376, Met508 | **Aromatic**, Hydrophobic |
| **6g** | -1.27 | -10.88 | 1 | Tyr118 | Tyr132, Hem601 | Hem601, Ile131, Tyr131, Phe126, Ile304, Leu376, Met508 | **Aromatic**, Hydrophobic |
| **Ketoconazole** | - | -8.23 | 1 | Tyr64 | Hem601 | Tyr118, Ile131, Tyr132, Hem601, Ile304, Leu300, Leu376, Met508 | **Aromatic**, Hydrophobic |

*Number of hydrogen bonds.

**Table S4.** Drug likeness predictions of tested compounds.

| No | MW | Number of HBA[a] | Number of HBD[b] | Log $P_{o/w}$ (iLOGP)[c] | Log S[d] | TPSA[e] | BBB permeant[f] | Lipinski, Ghose, Veber, Egan, and Muegge violations | Bioavailability Score | Drug-likeness model score |
|---|---|---|---|---|---|---|---|---|---|---|
| **5a** | 215.27 | 1 | 2 | 1.55 | Moderately soluble | 82.94 | No | 0 | 0.55 | -1.71 |
| **5b** | 245.30 | 2 | 2 | 1.87 | Moderately soluble | 92.17 | No | 0 | 0.55 | -1.16 |
| **5c** | 245.30 | 2 | 2 | 1.87 | Moderately soluble | 92.17 | No | 0 | 0.55 | -1.16 |
| **5d** | 229.30 | 1 | 2 | 1.75 | Moderately soluble | 82.94 | No | 0 | 0.55 | -1.57 |
| **5e** | 233.26 | 2 | 2 | 1.66 | Moderately soluble | 60.17 | No | 0 | 0.55 | -1.38 |
| **5f** | 259.33 | 2 | 2 | 2.08 | Moderately soluble | 92.17 | No | 0 | 0.55 | -0.57 |
| **5g** | 273.35 | 2 | 1 | 2.54 | Moderately soluble | 81.31 | No | 0 | 0.55 | -0.90 |
| **5h** | 363.48 | 2 | 1 | 3.62 | Poorly soluble | 67.32 | Yes | 0 | 0.55 | -0.29 |
| **5i** | 273.35 | 2 | 2 | 2.45 | Moderately soluble | 78.18 | Yes | 0 | 0.55 | -0.51 |
| **5j** | 287.38 | 2 | 1 | 2.89 | Moderately soluble | 67.32 | Yes | 0 | 0.55 | -0.83 |
| **5k** | 257.35 | 1 | 1 | 2.61 | Moderately soluble | 58.09 | Yes | 0 | 0.55 | -1.42 |
| **5l** | 257.35 | 1 | 1 | 2.47 | Moderately soluble | 68.95 | Yes | 0 | 0.55 | -1.22 |
| **5m** | 263.75 | 1 | 2 | 2.10 | Moderately soluble | 82.94 | No | 0 | 0.55 | -0.74 |
| **5n** | 259.33 | 2 | 2 | 2.16 | Moderately soluble | 92.17 | No | 0 | 0.55 | -0.96 |
| **5o** | 243.33 | 1 | 2 | 1.96 | Moderately soluble | 82.94 | No | 0 | 0.55 | -1.01 |
| **5p** | 243.33 | 1 | 1 | 2.17 | Moderately soluble | 72.08 | Yes | 0 | 0.55 | -1.48 |
| **5q** | 243.33 | 1 | 1 | 2.17 | Moderately soluble | 72.08 | Yes | 0 | 0.55 | -1.47 |
| **5r** | 257.35 | 1 | 1 | 2.34 | Moderately soluble | 72.08 | Yes | 0 | 0.55 | -1.42 |
| **5s** | 229.30 | 1 | 2 | 1.78 | Moderately soluble | 82.94 | No | 0 | 0.55 | -0.88 |
| **5t** | 271.38 | 1 | 1 | 2.82 | Moderately soluble | 58.09 | Yes | 0 | 0.55 | -1.34 |
| **5u** | 257.35 | 1 | 2 | 2.31 | Moderately soluble | 68.95 | Yes | 0 | 0.55 | -0.96 |

| | | | | | | | | | | |
|---|---|---|---|---|---|---|---|---|---|---|
| **5v** | 259.33 | 2 | 2 | 2.15 | Moderately soluble | 78.18 | Yes | 0 | 0.55 | -1.09 |
| **5w** | 243.33 | 1 | 1 | 2.17 | Moderately soluble | 72.08 | Yes | 0 | 0.55 | -1.48 |
| **5x** | 270.35 | 2 | 2 | 2.15 | Moderately soluble | 81.31 | No | 0 | 0.55 | -1.44 |

a) number of hydrogen bond acceptors; b) number of hydrogen bond donors; c) lipophilicity; d) Water solubility (SILICOS-IT [S=Soluble]); e) topological polar surface area (Å$^2$); f) Blood Brain Barrier permeant;

## $^{1}$H-NMR AND $^{13}$C-NMR OF COMPOUNDS

### Compound 5a

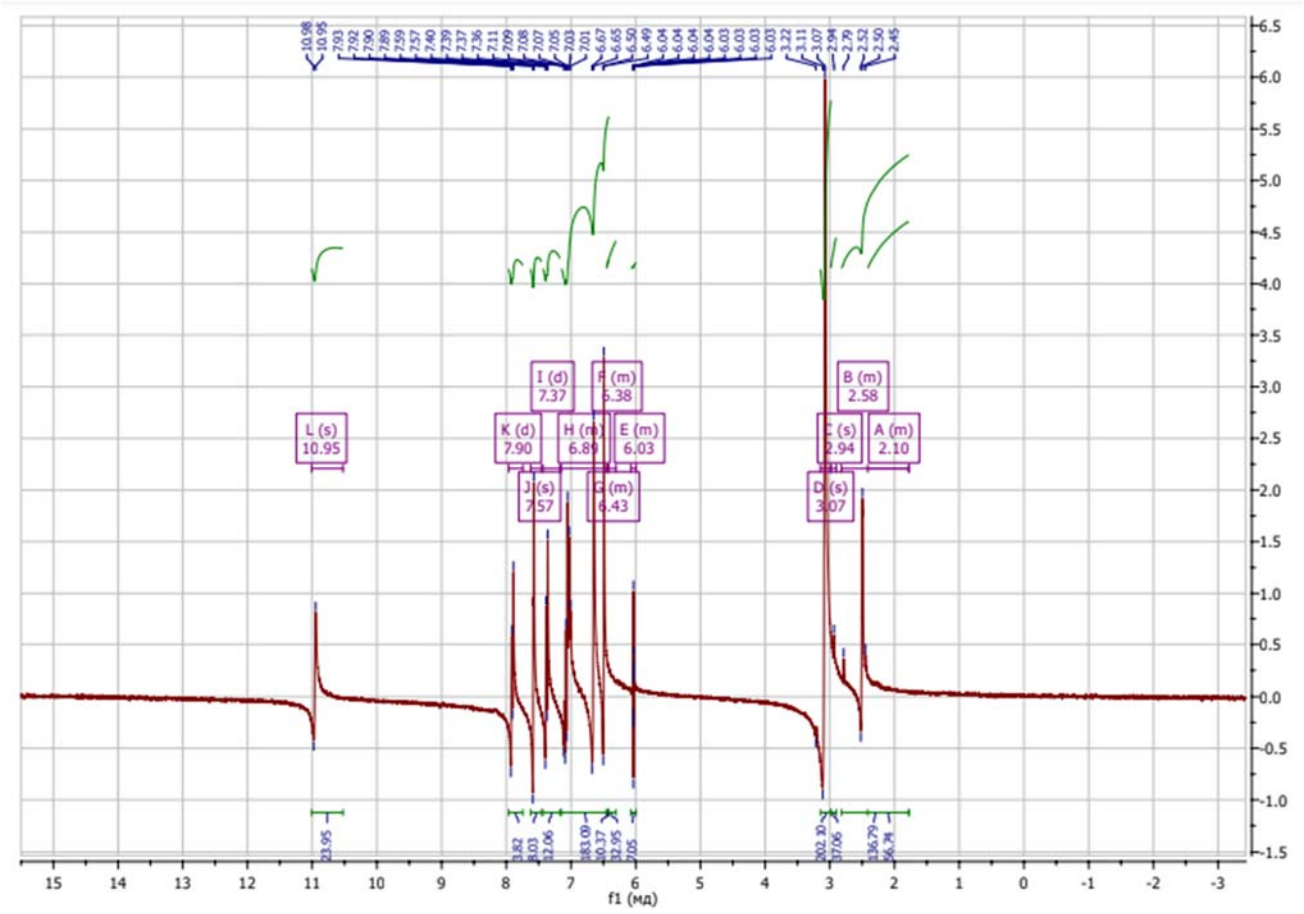

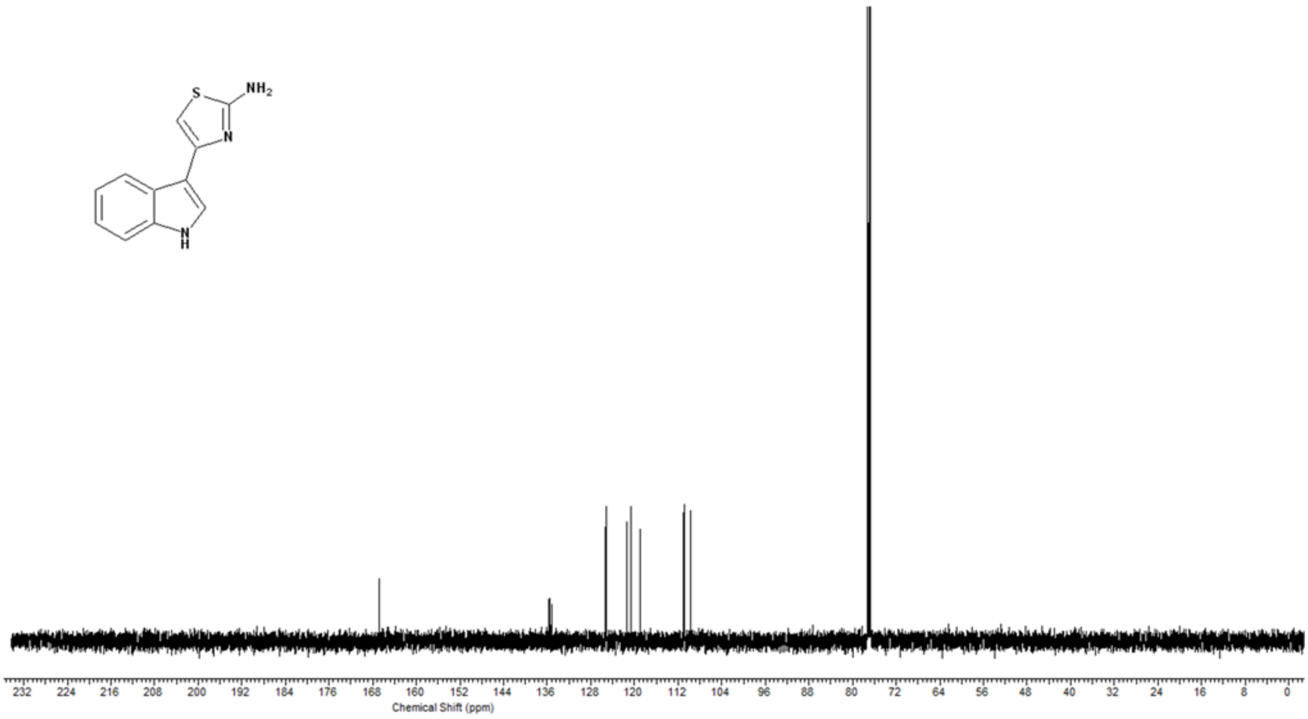
S
NH2
N
H
232 224 216 208 200 192 184 176 168 160 152 144 136 128 120 112 104 96 88 80 72 64 56 48 40 32 24 16 8 0
Chemical Shift (ppm)

**Compound 5b**

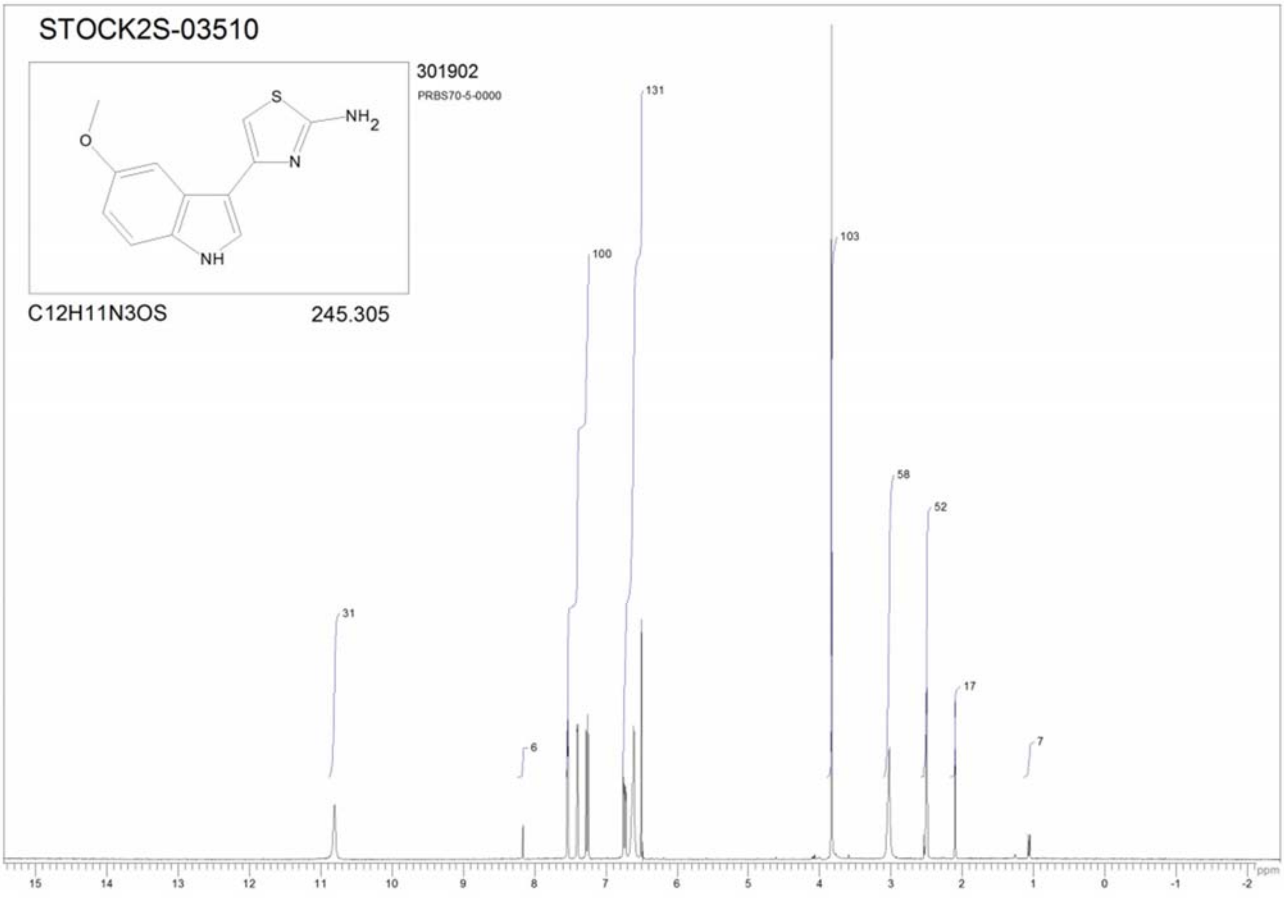
STOCK2S-03510
301902
PRBS70-5-0000
O
S
NH2
N
NH
C12H11N3OS
245.305
131
100
103
58
52
31
17
6
7
15 14 13 12 11 10 9 8 7 6 5 4 3 2 1 0 -1 -2
ppm

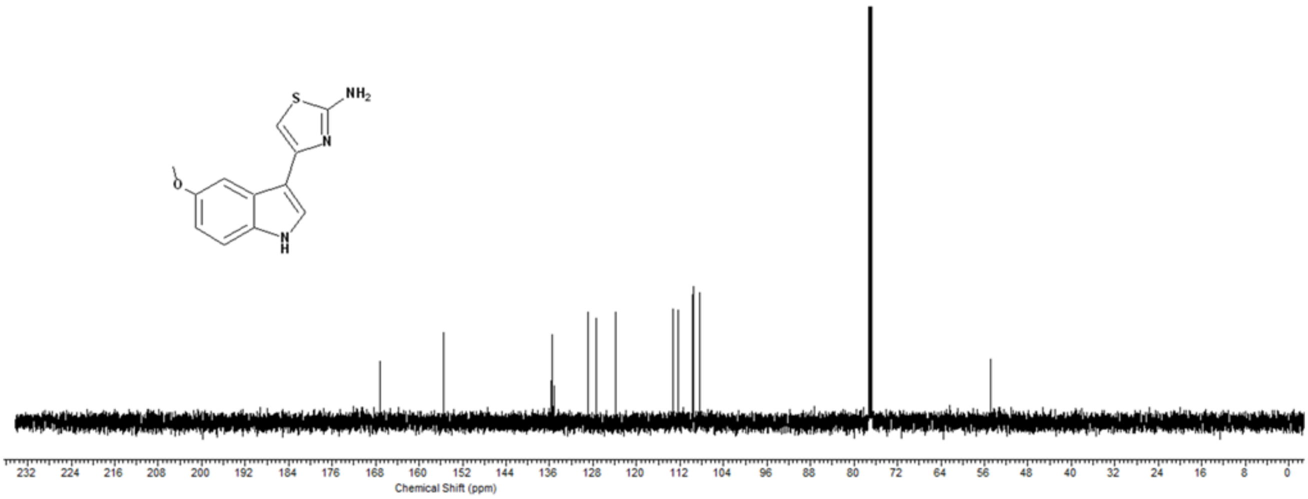
NH2
S
N
O
H
232 224 216 208 200 192 184 176 168 160 152 144 136 128 120 112 104 96 88 80 72 64 56 48 40 32 24 16 8 0
Chemical Shift (ppm)

**Compound 5c**

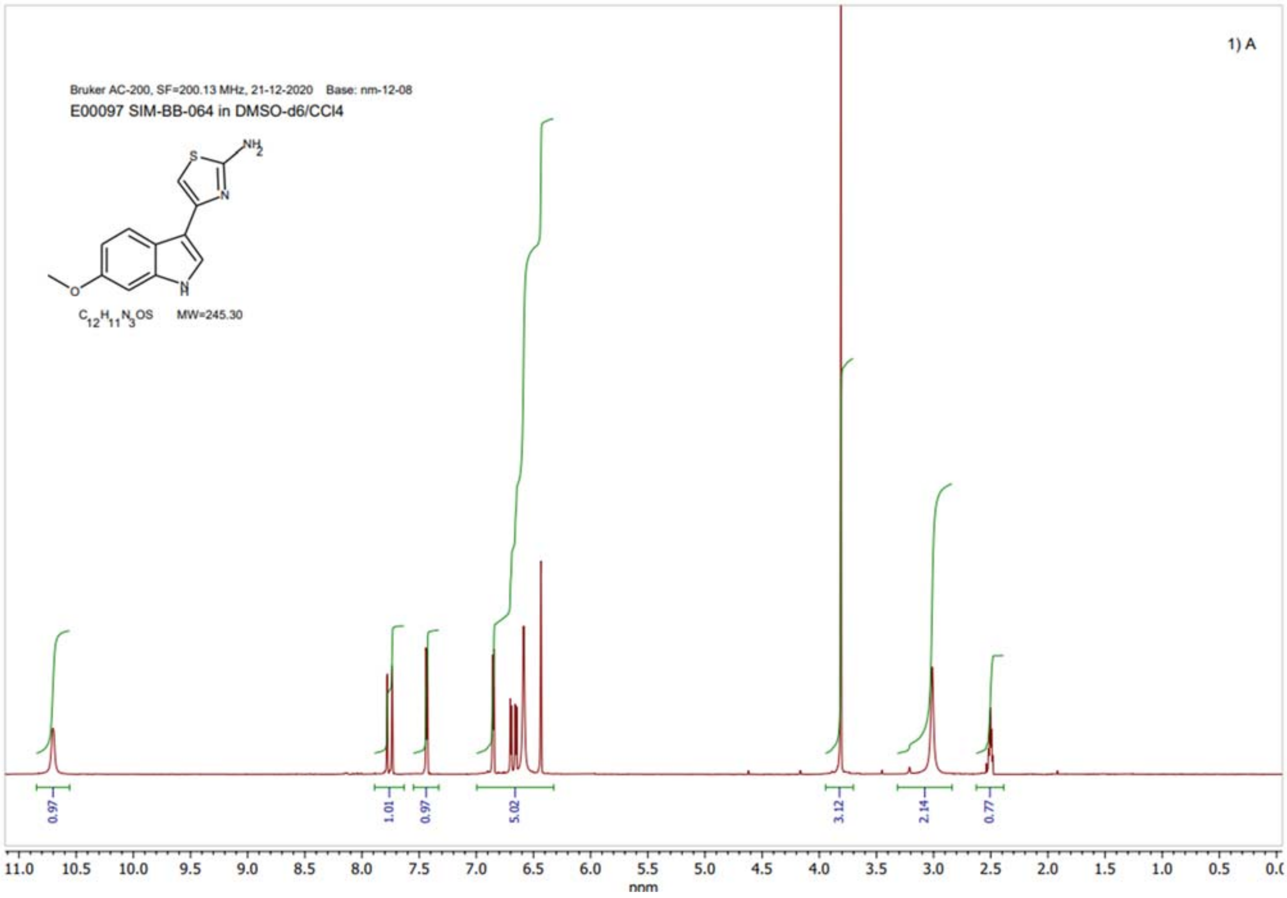
1) A
Bruker AC-200, SF=200.13 MHz, 21-12-2020 Base: nm-12-08
E00097 SIM-BB-064 in DMSO-d6/CCl4
NH2
S
N
O
H
C12H11N3OS MW=245.30
0.97
1.01
0.97
5.02
3.12
2.14
0.77
11.0 10.5 10.0 9.5 9.0 8.5 8.0 7.5 7.0 6.5 6.0 5.5 5.0 4.5 4.0 3.5 3.0 2.5 2.0 1.5 1.0 0.5
ppm

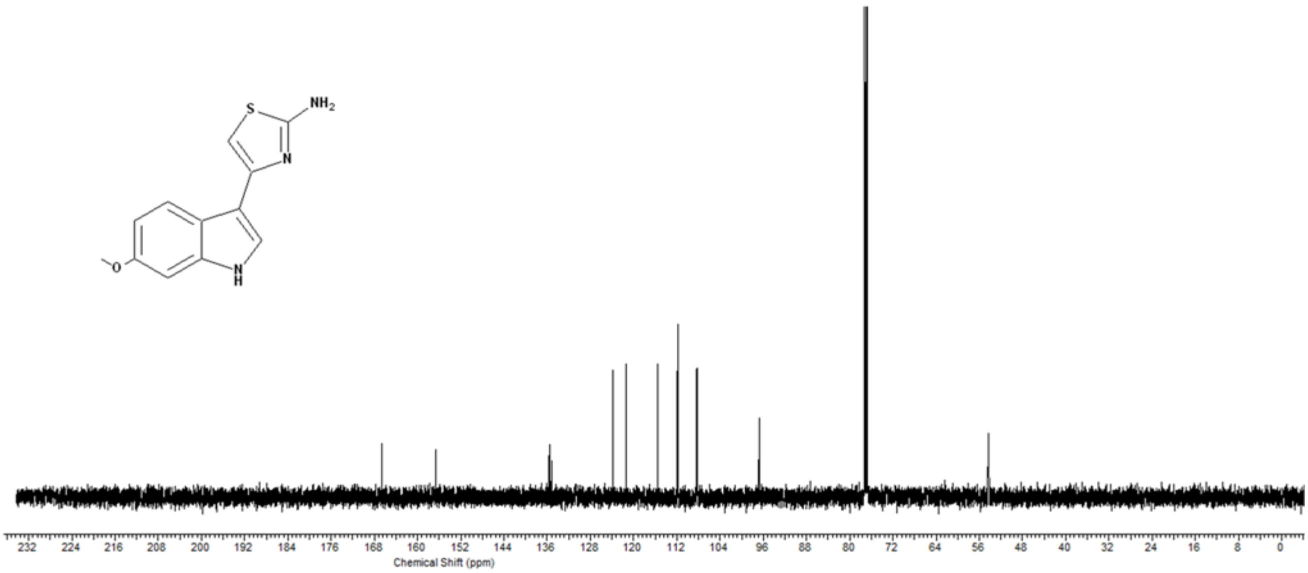
NH2
S
N
O
H
232 224 216 208 200 192 184 176 168 160 152 144 136 128 120 112 104 96 88 80 72 64 56 48 40 32 24 16 8 0
Chemical Shift (ppm)

**Compound 5d**

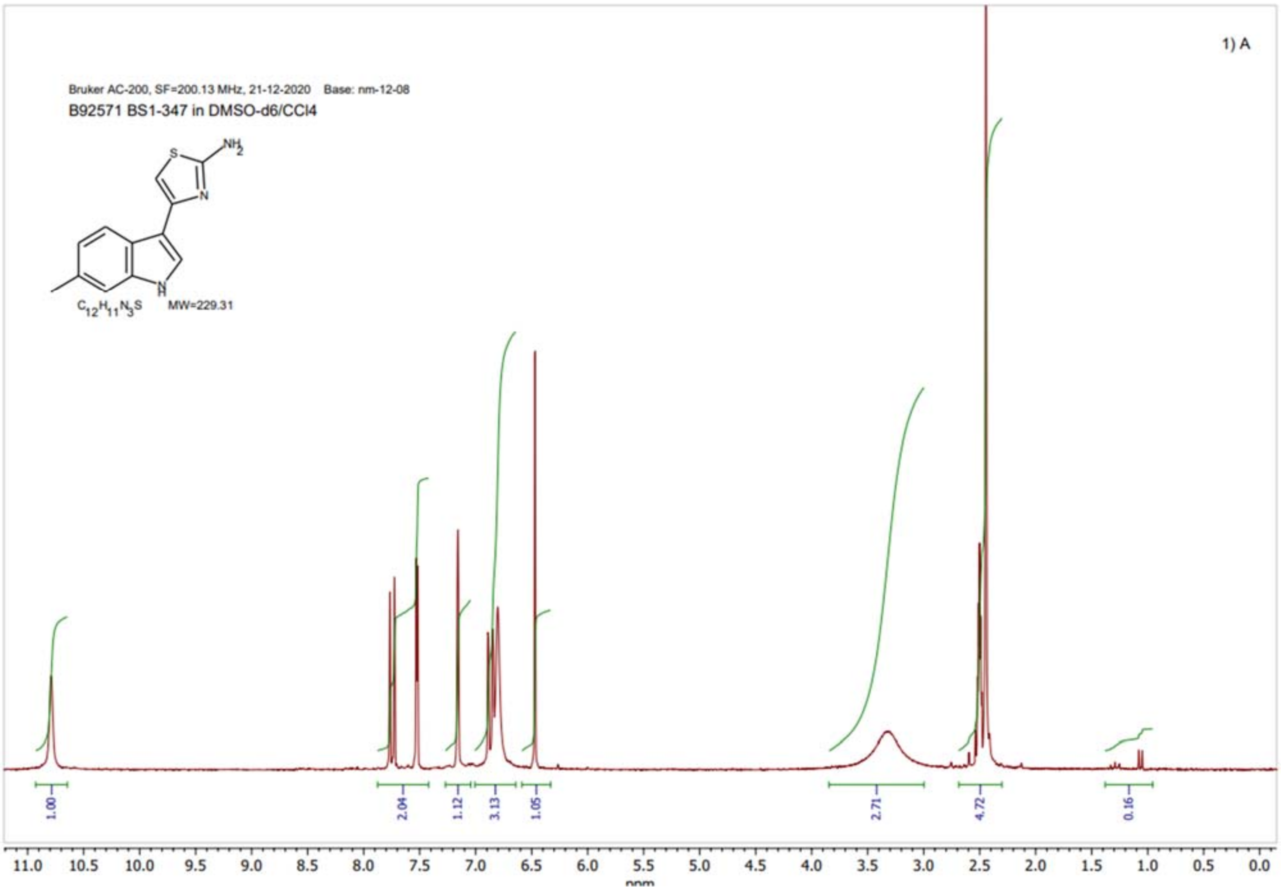
1) A
Bruker AC-200, SF=200.13 MHz, 21-12-2020 Base: nm-12-08
B92571 BS1-347 in DMSO-d6/CCl4
S
NH2
N
H
C12H11N3S
MW=229.31
1.00
2.04
1.12
3.13
1.05
2.71
4.72
0.16
11.0 10.5 10.0 9.5 9.0 8.5 8.0 7.5 7.0 6.5 6.0 5.5 5.0 4.5 4.0 3.5 3.0 2.5 2.0 1.5 1.0 0.5 0.0

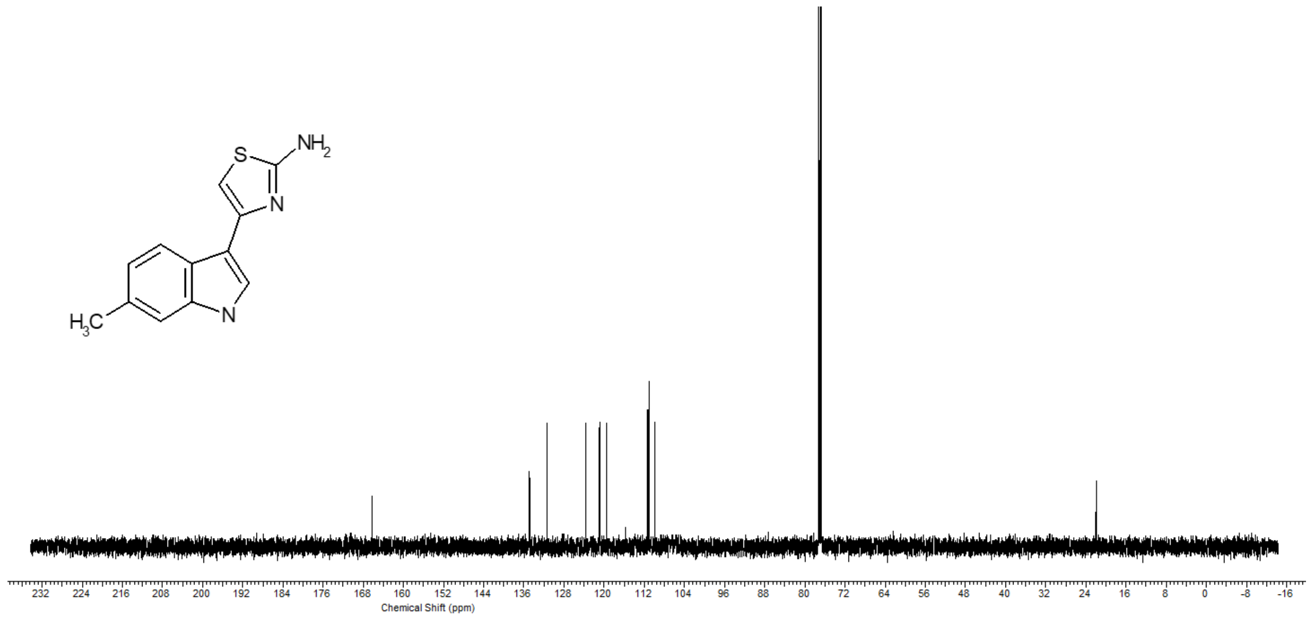
NH2
S
N
N
H3C
Chemical Shift (ppm)

**Compound 5e**

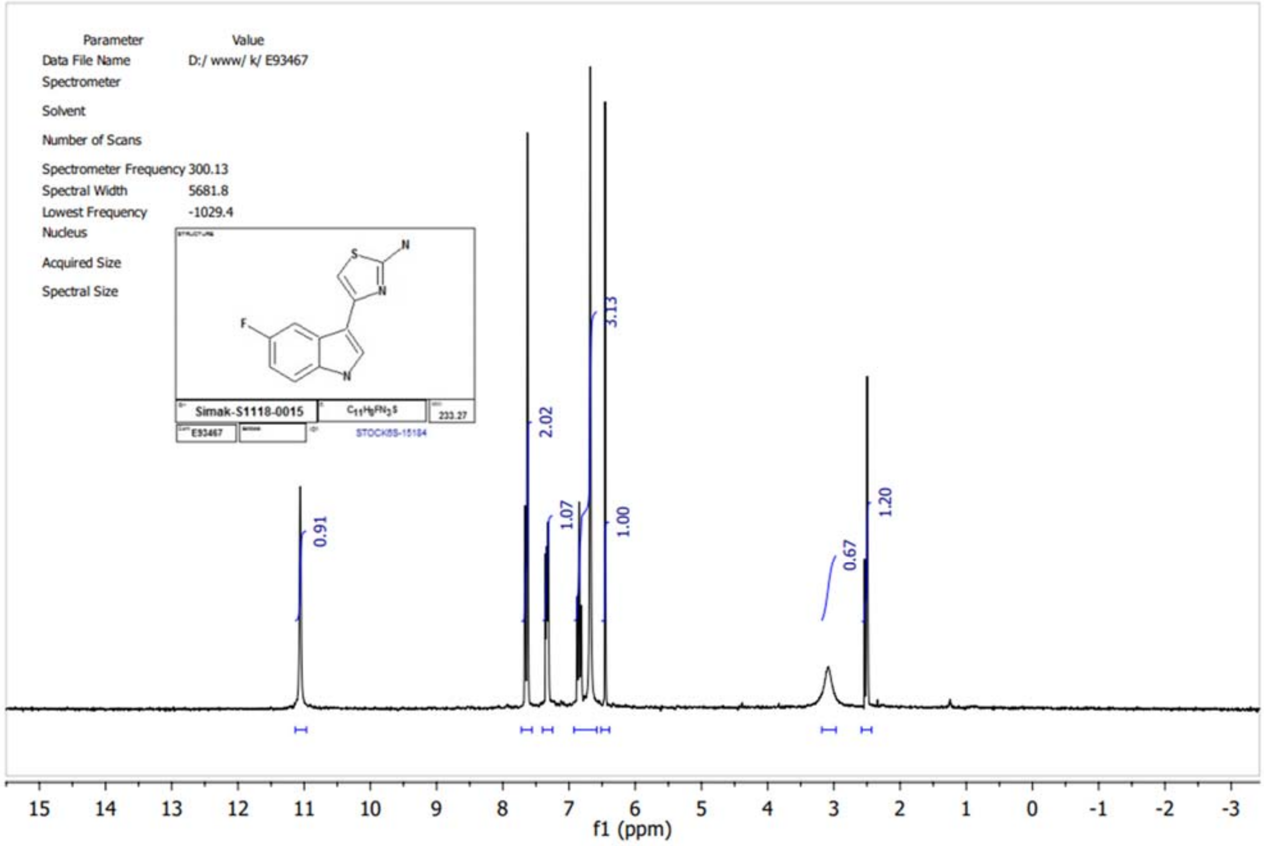
Parameter
Value
Data File Name
D:/ www/ k/ E93467
Spectrometer
Solvent
Number of Scans
Spectrometer Frequency 300.13
Spectral Width
5681.8
Lowest Frequency
-1029.4
Nucleus
Acquired Size
Spectral Size
Simak-S1118-0015
233.27
E93467
STOCKBS-15184
0.91
2.02
1.07
3.13
1.00
0.67
1.20
f1 (ppm)

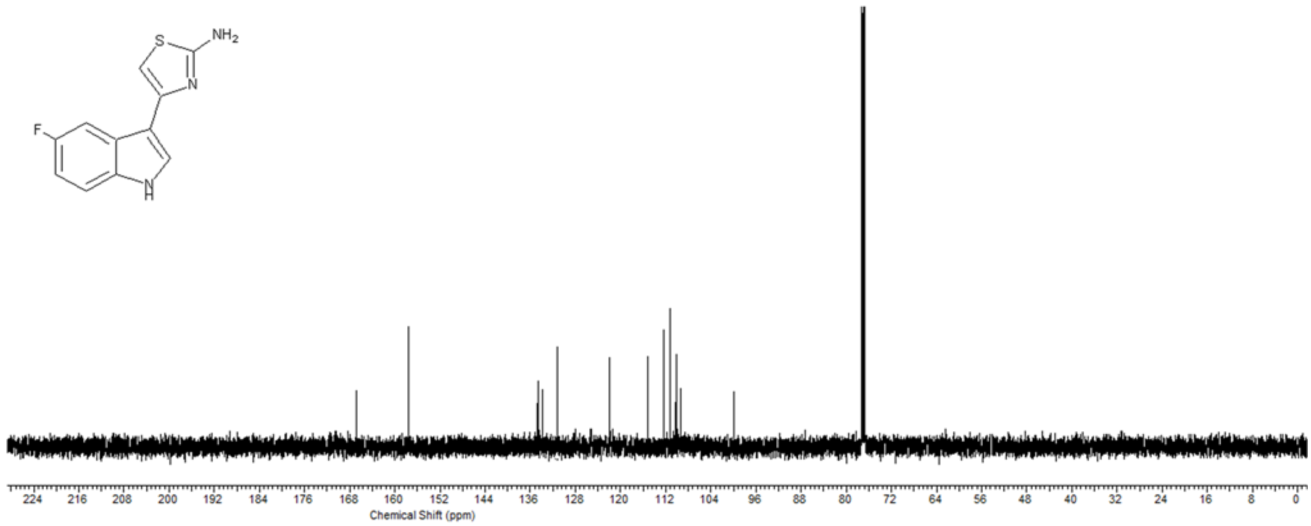

NH2
S
N
F
H
224 216 208 200 192 184 176 168 160 152 144 136 128 120 112 104 96 88 80 72 64 56 48 40 32 24 16 8 0
Chemical Shift (ppm)


**Compound 5f**

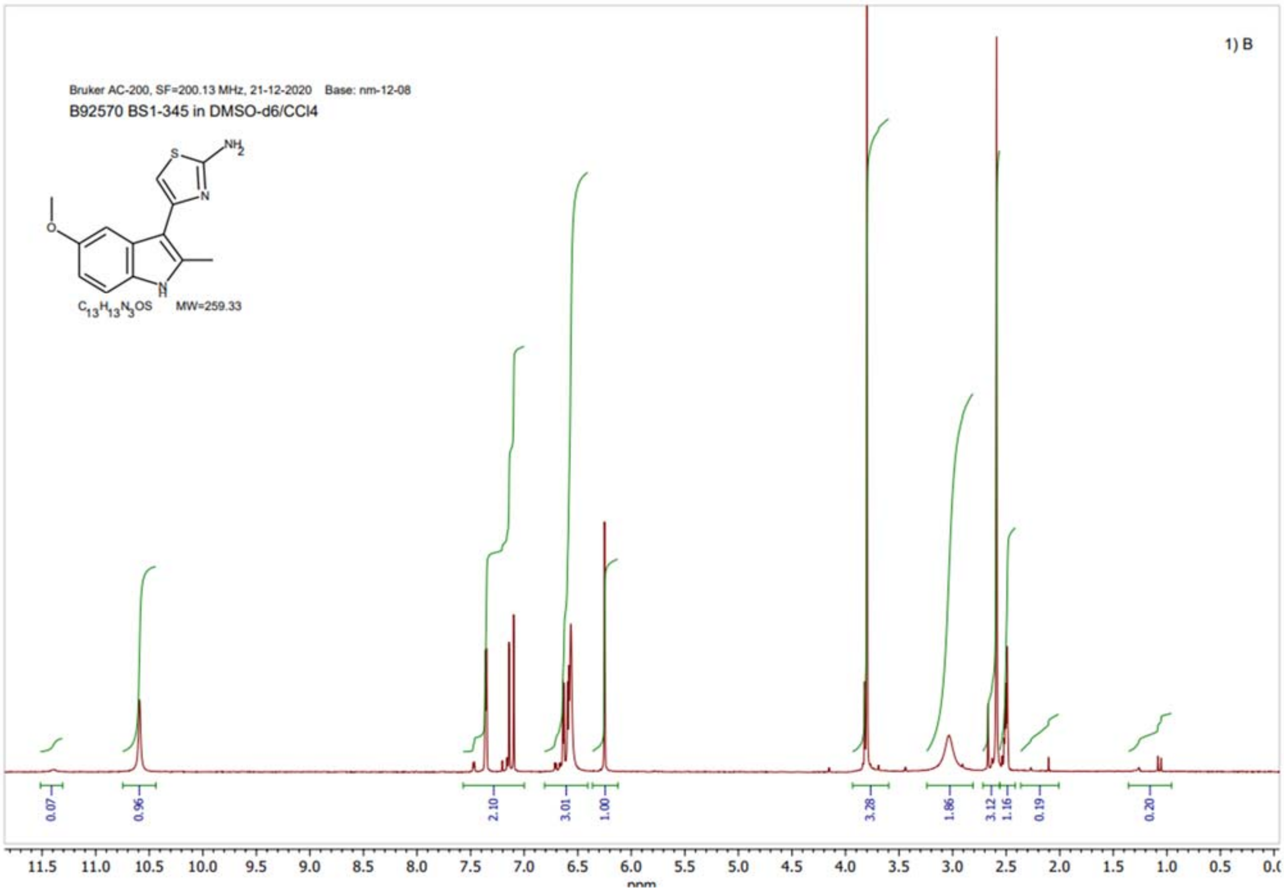

1) B
Bruker AC-200, SF=200.13 MHz, 21-12-2020 Base: nm-12-08
B92570 BS1-345 in DMSO-d6/CCl4
NH2
S
N
O
H
C13H13N3OS MW=259.33
0.07
0.96
2.10
3.01
1.00
3.28
1.86
3.12
1.16
0.19
0.20
11.5 11.0 10.5 10.0 9.5 9.0 8.5 8.0 7.5 7.0 6.5 6.0 5.5 5.0 4.5 4.0 3.5 3.0 2.5 2.0 1.5 1.0 0.5 0.0

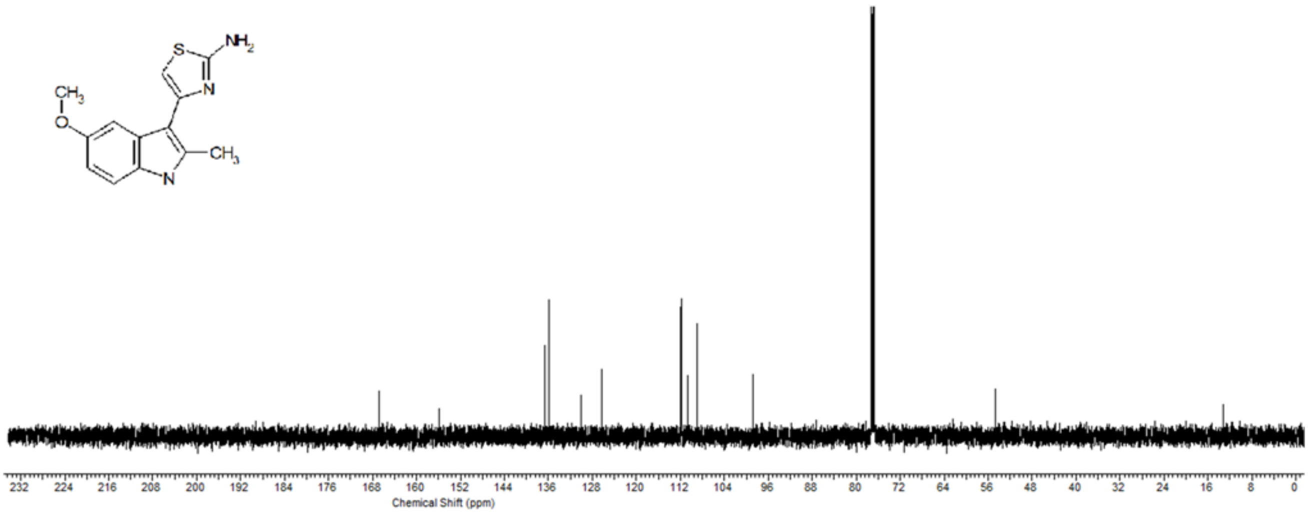


**Compound 5g**

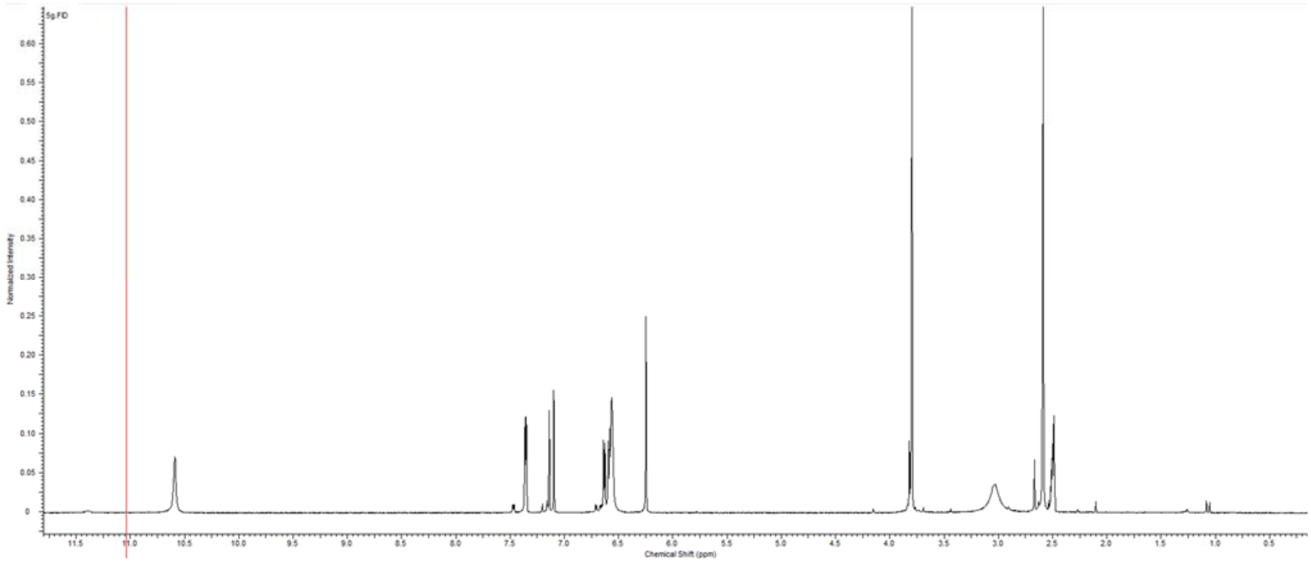

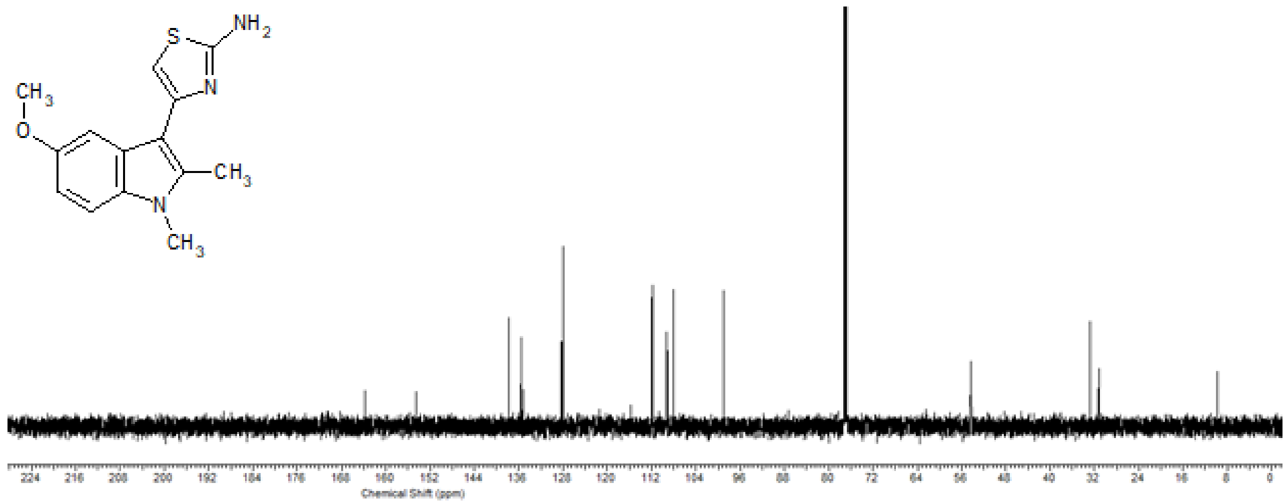


**Compound 5h**

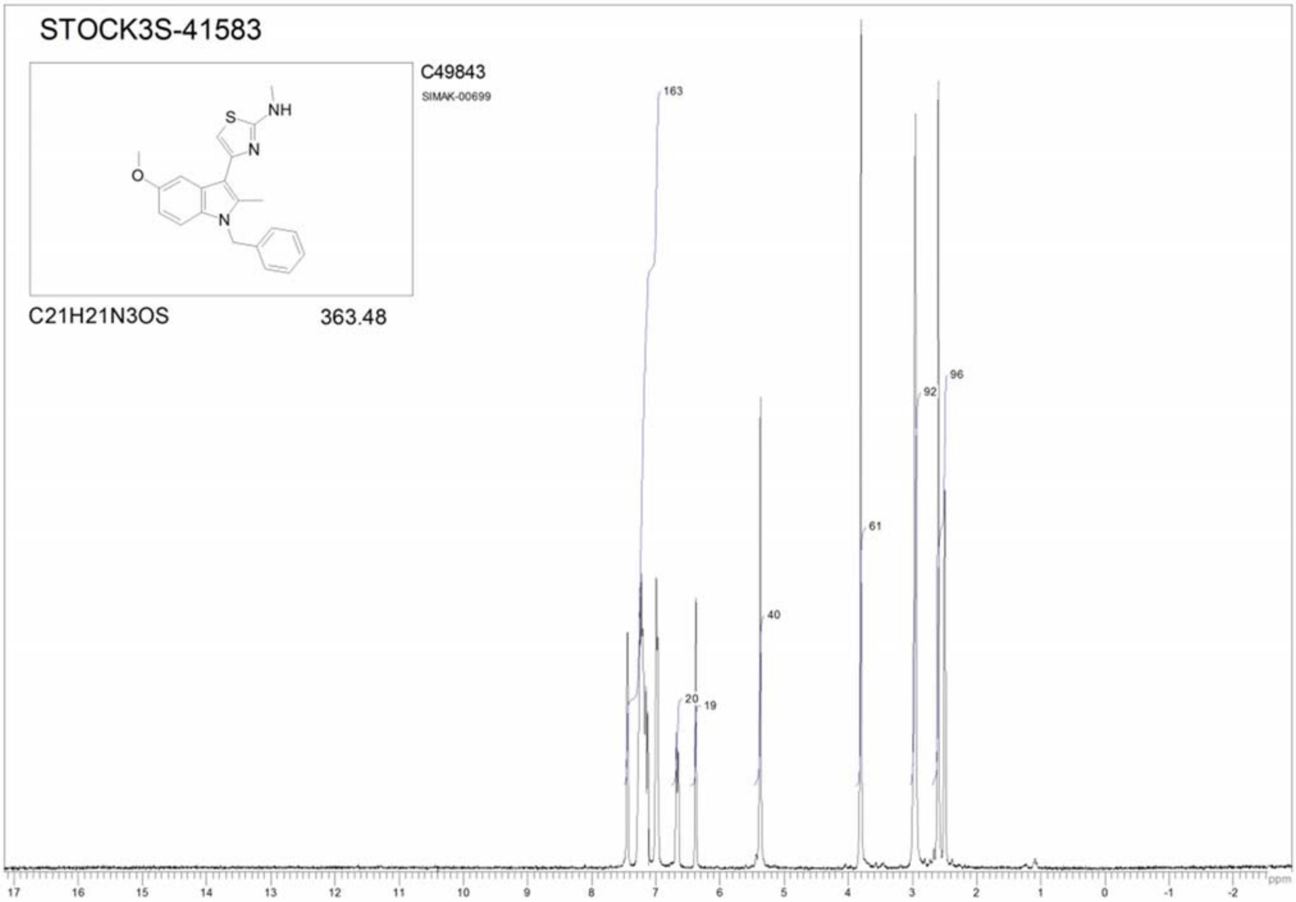
STOCK3S-41583
C49843
SIMAK-00699
NH
S
N
O
N
C21H21N3OS
363.48
163
20
19
40
61
92
96
ppm

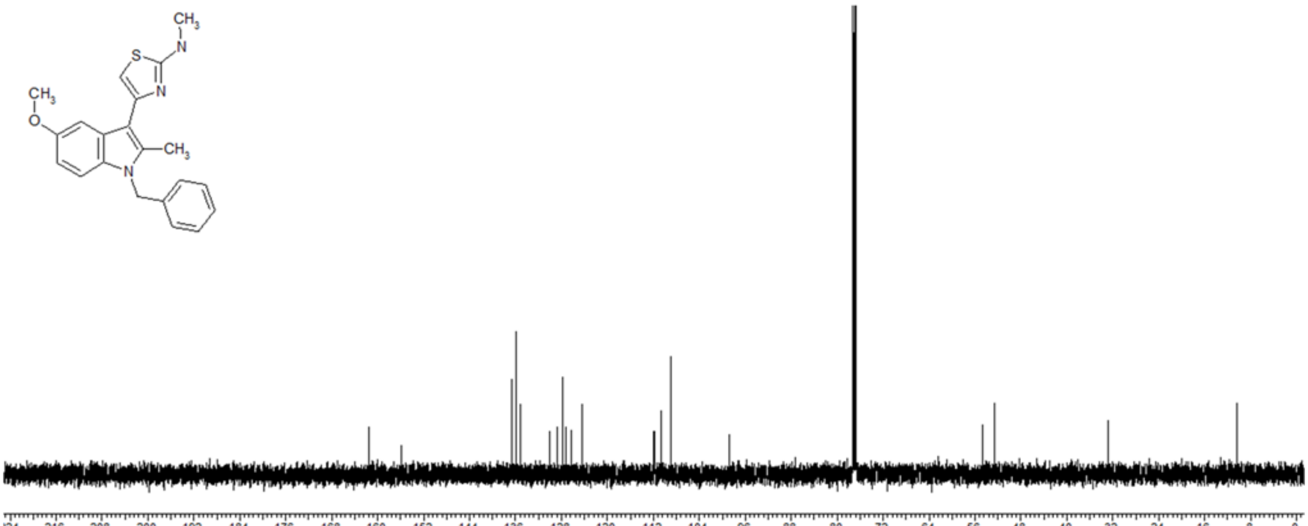
CH3
N
S
N
CH3
O
CH3
N
Chemical Shift (ppm)

**Compound 5i**

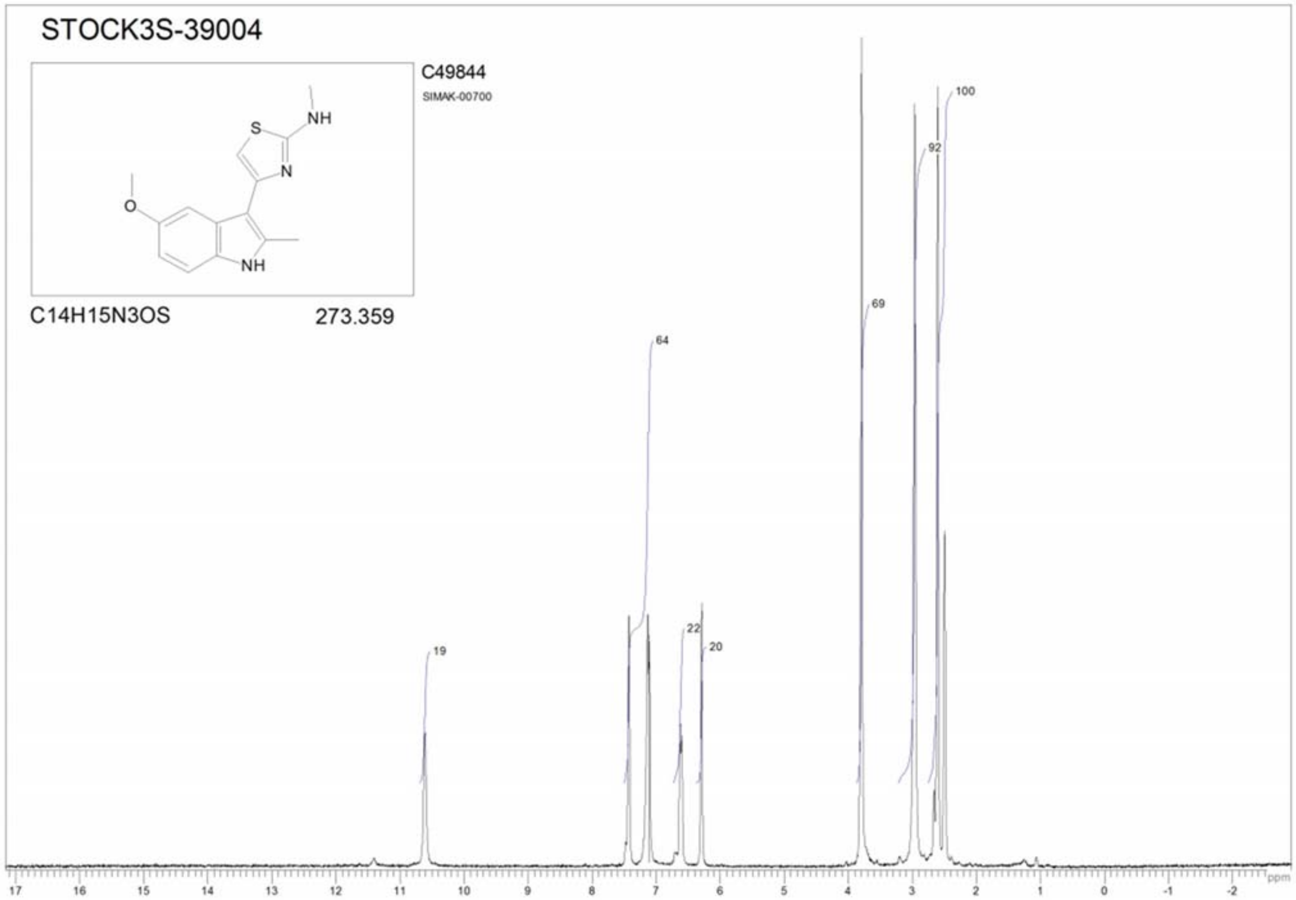
STOCK3S-39004
C49844
SIMAK-00700
C14H15N3OS
273.359
ppm

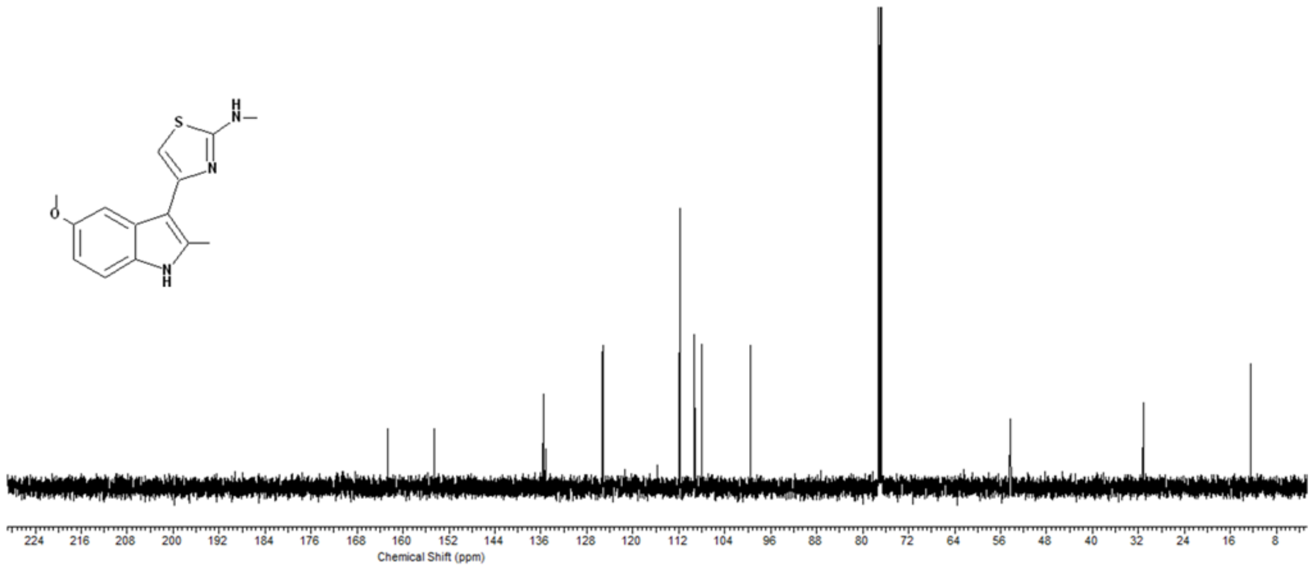
Chemical Shift (ppm)

**Compound 5j**

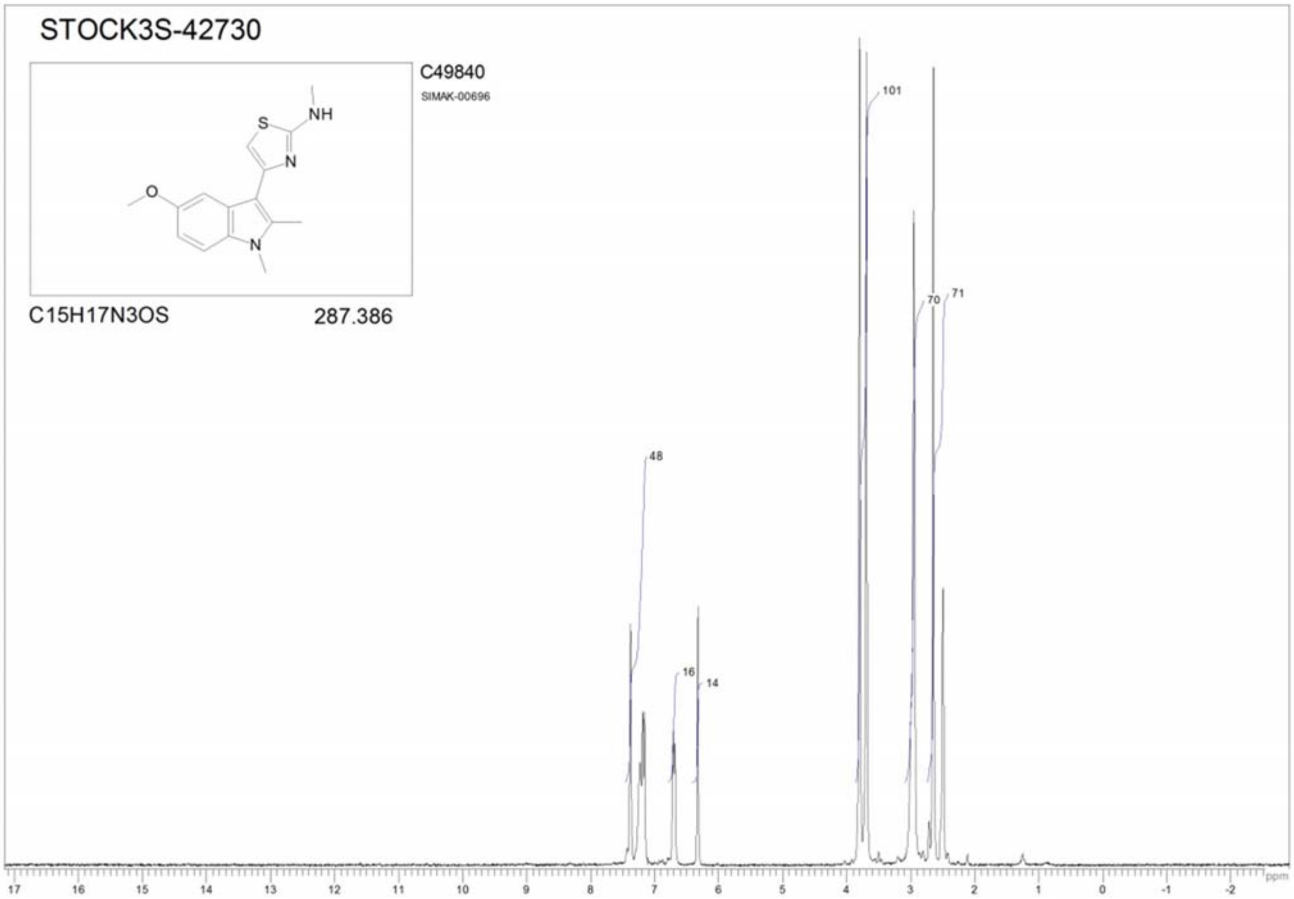
STOCK3S-42730
C49840
SIMAK-00696
C15H17N3OS
287.386
NH
S
N
O
N
101
70
71
48
16
14
ppm

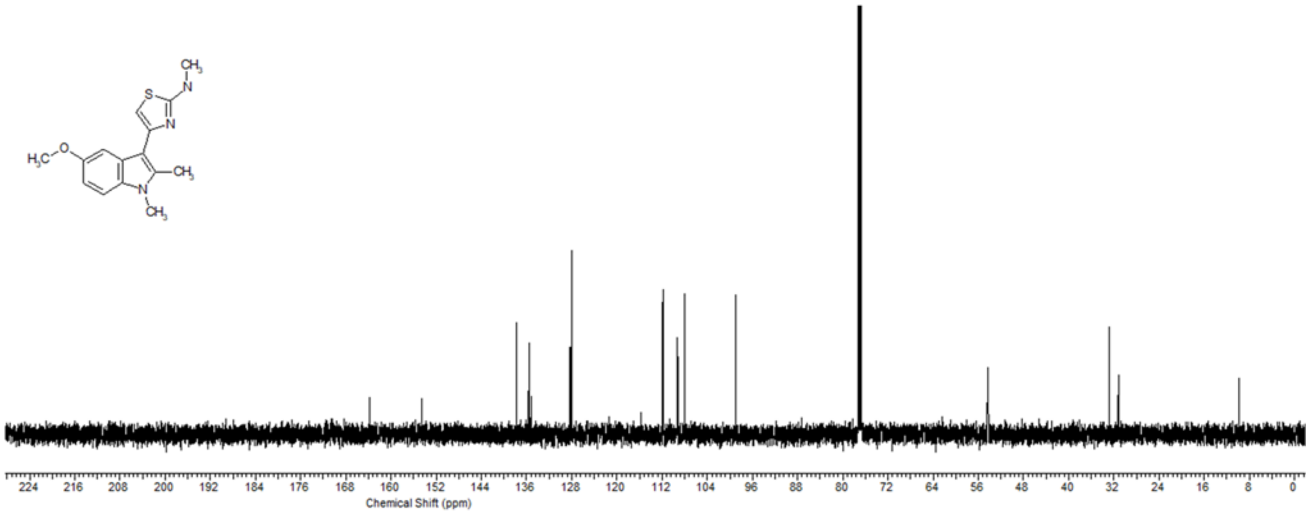
CH3
N
S
N
H3C
O
CH3
N
CH3
Chemical Shift (ppm)

**Compound 5k**

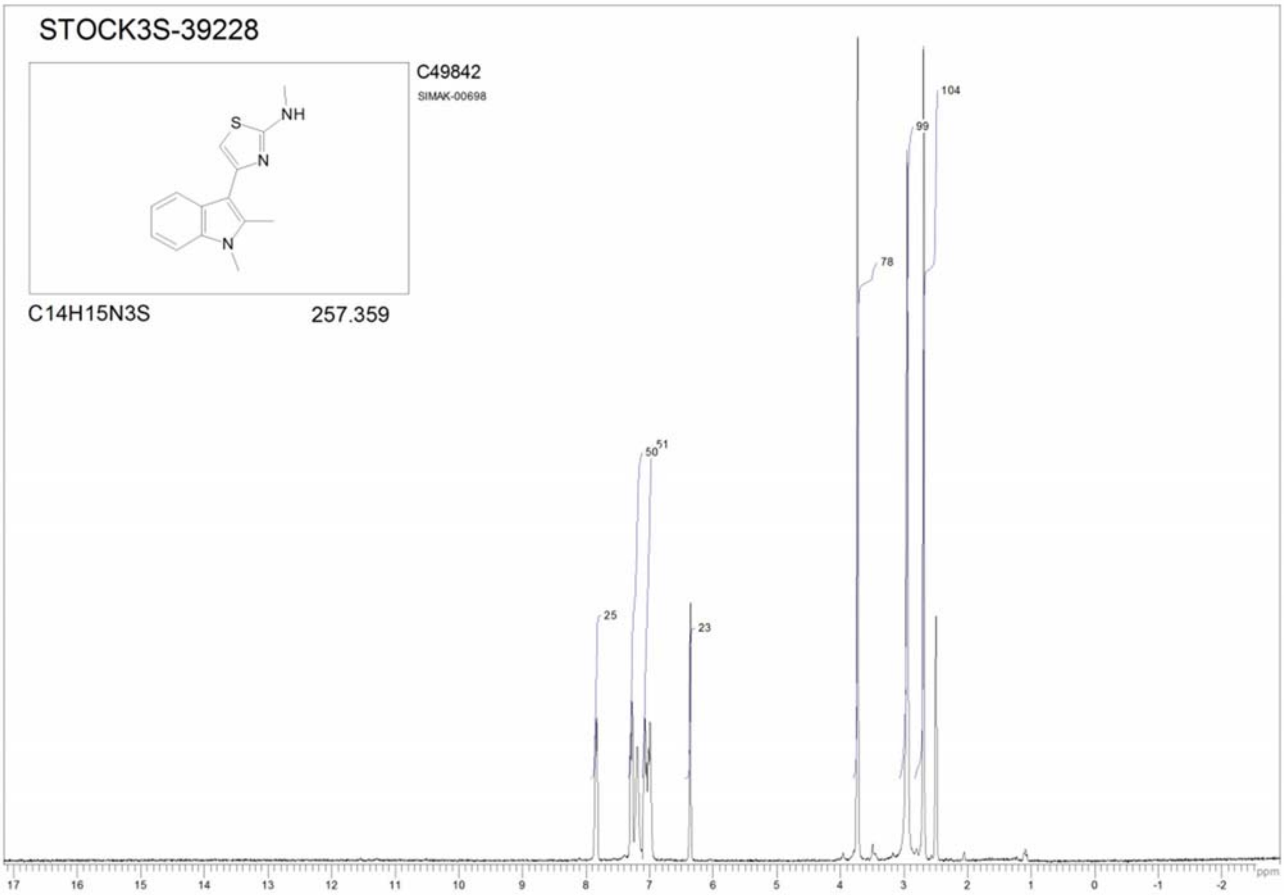
STOCK3S-39228
C49842
SIMAK-00698
C14H15N3S
257.359
ppm

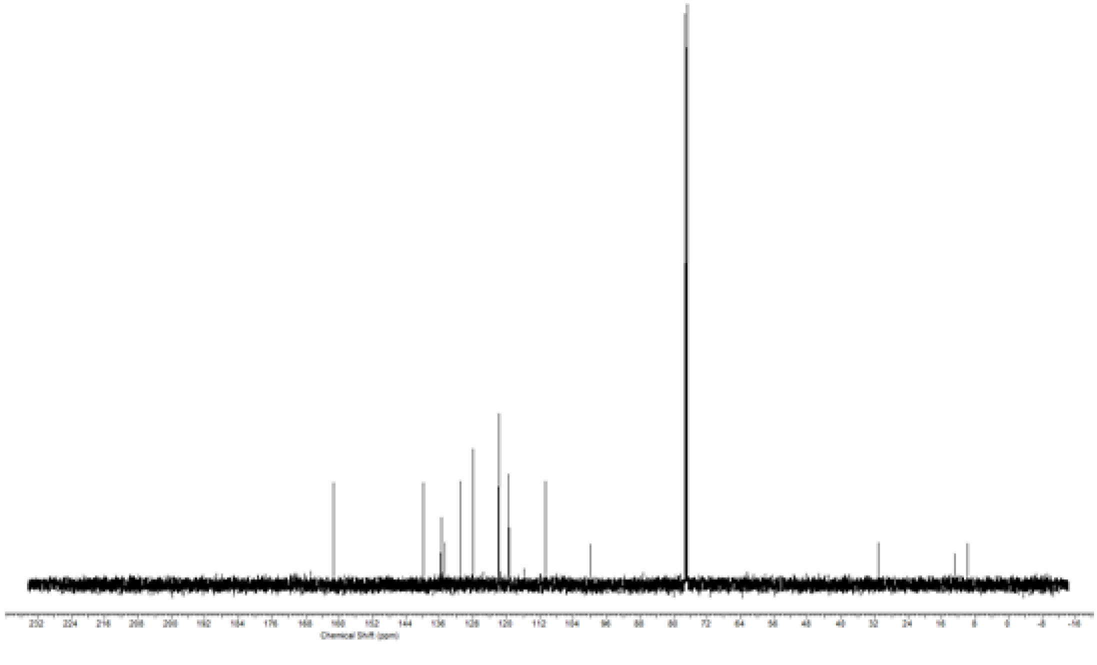
Chemical Shift (ppm)

**Compound 5l**

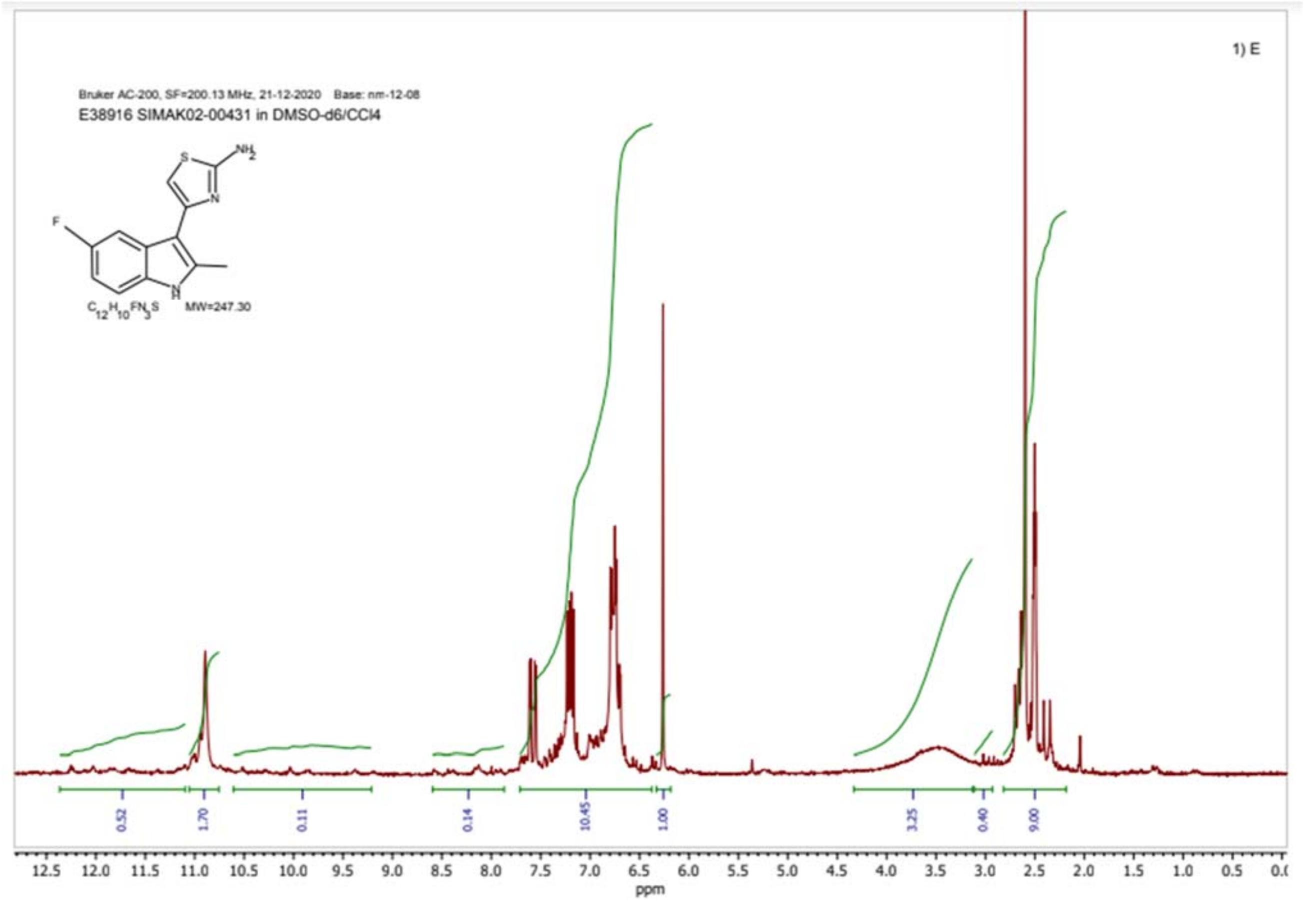
1) E
Bruker AC-200, SF=200.13 MHz, 21-12-2020 Base: nm-12-08
E38916 SIMAK02-00431 in DMSO-d6/CCl4
MW=247.30
0.52
1.70
0.11
0.14
10.45
1.00
3.25
0.40
9.00
ppm

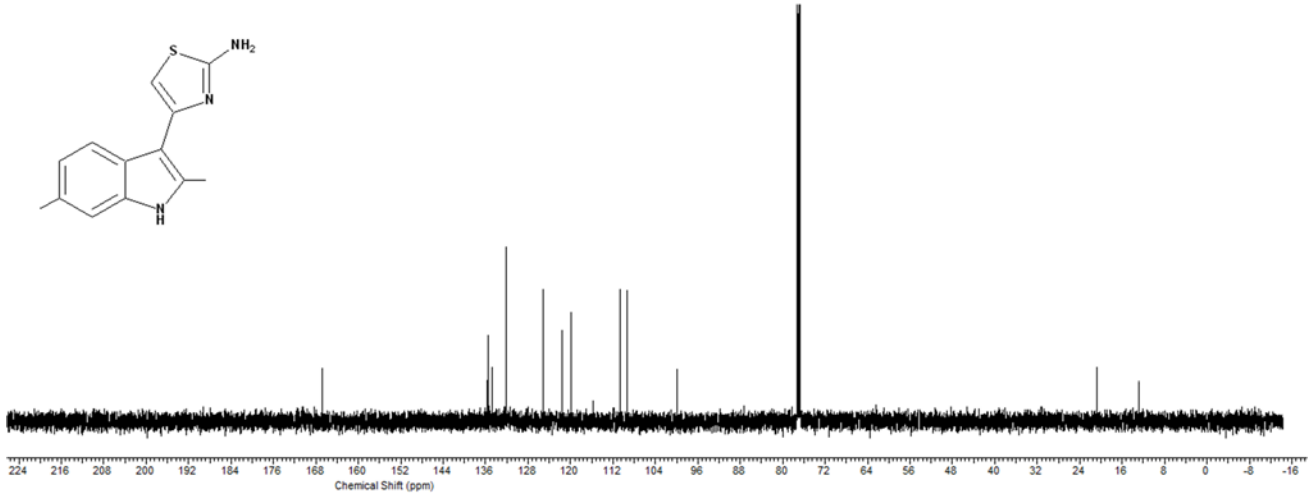
S
NH2
N
H
Chemical Shift (ppm)

**Compound 5m**

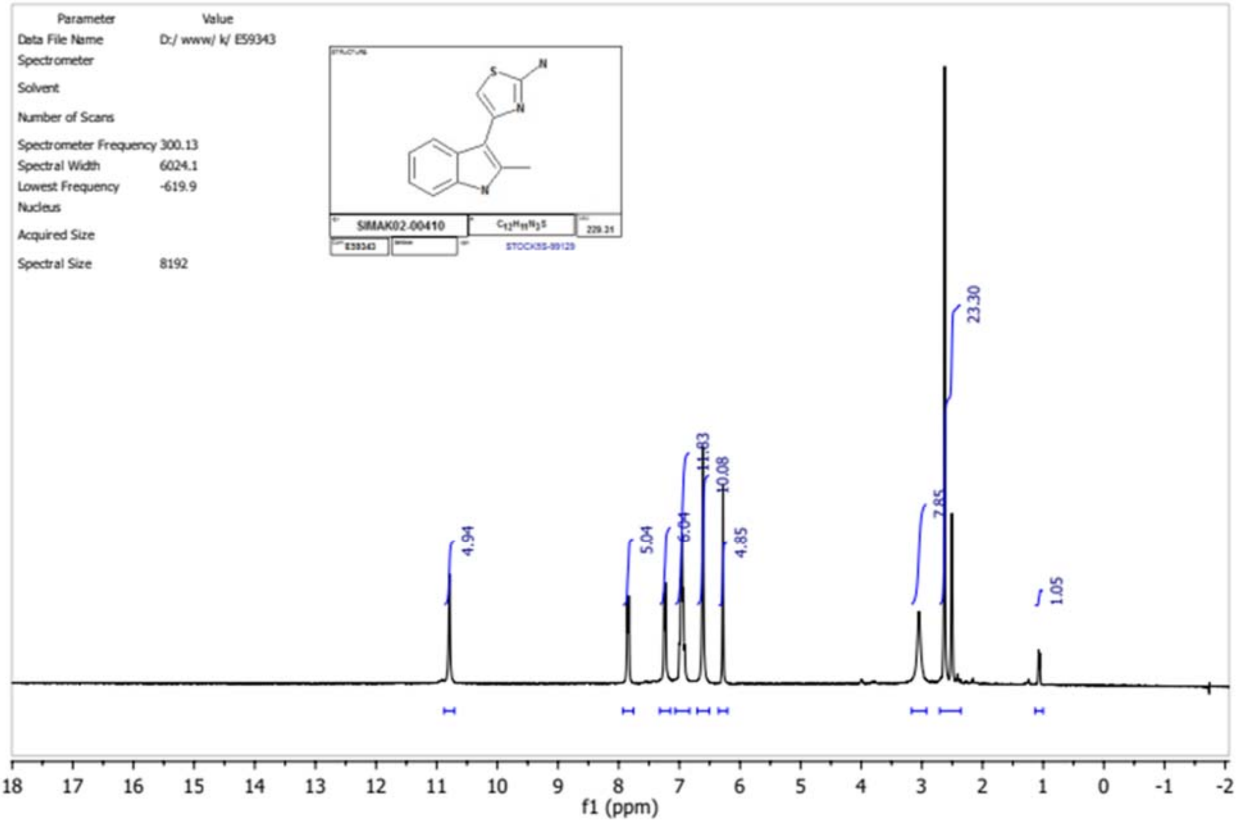
Parameter
Value
Data File Name
D:/ www/ k/ E59343
Spectrometer
Solvent
Number of Scans
Spectrometer Frequency 300.13
Spectral Width
6024.1
Lowest Frequency
-619.9
Nucleus
Acquired Size
Spectral Size
8192
SIMAK02-00410
229.31
STOCKSS-99129
4.94
5.04
6.04
11.83
10.08
4.85
7.85
23.30
1.05
f1 (ppm)

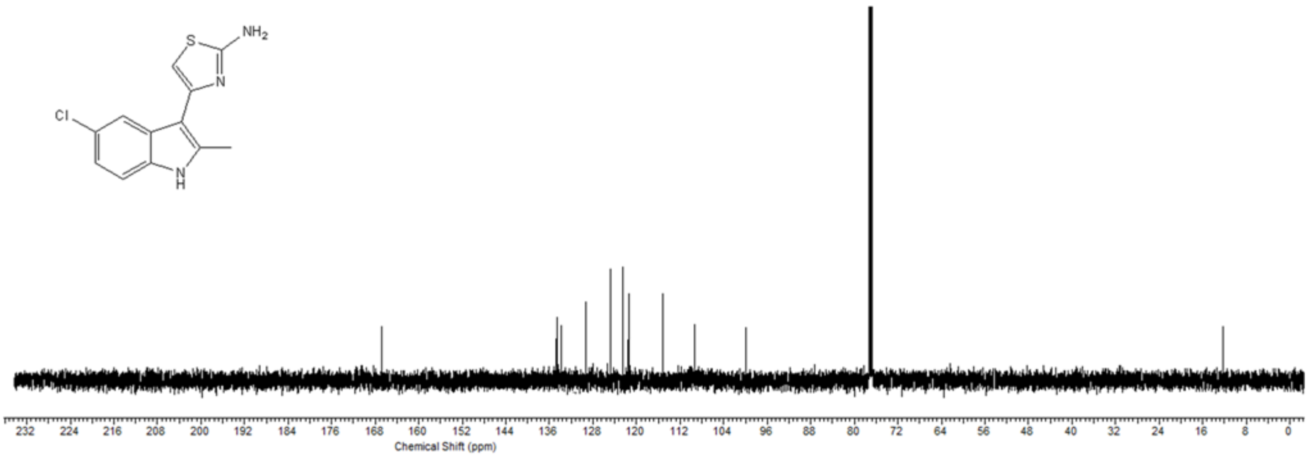
NH2
S
N
Cl
H
Chemical Shift (ppm)

**Compound 5n**

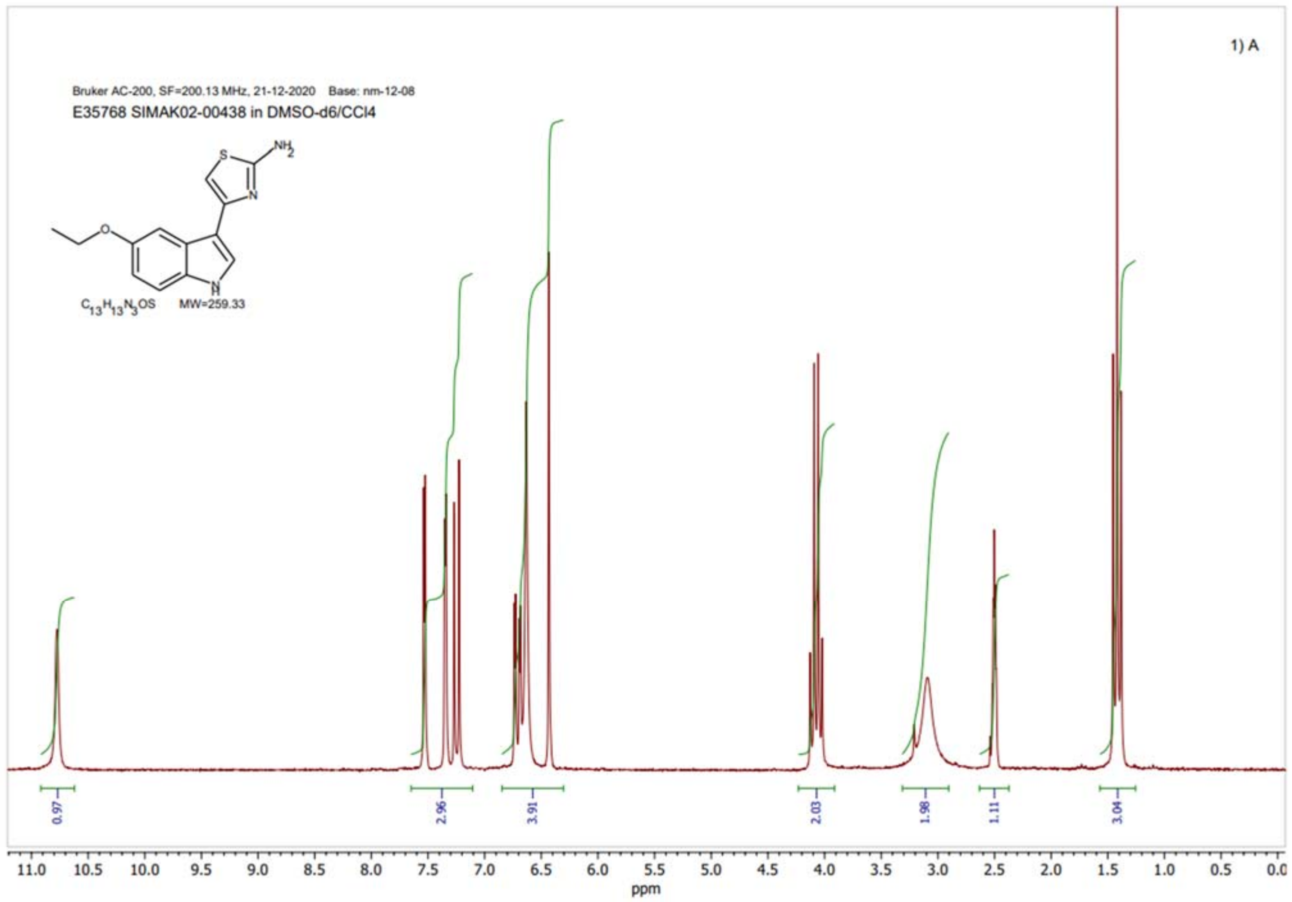
1) A
Bruker AC-200, SF=200.13 MHz, 21-12-2020 Base: nm-12-08
E35768 SIMAK02-00438 in DMSO-d6/CCl4
C13H13N3OS MW=259.33
0.97
2.96
3.91
2.03
1.98
1.11
3.04
ppm

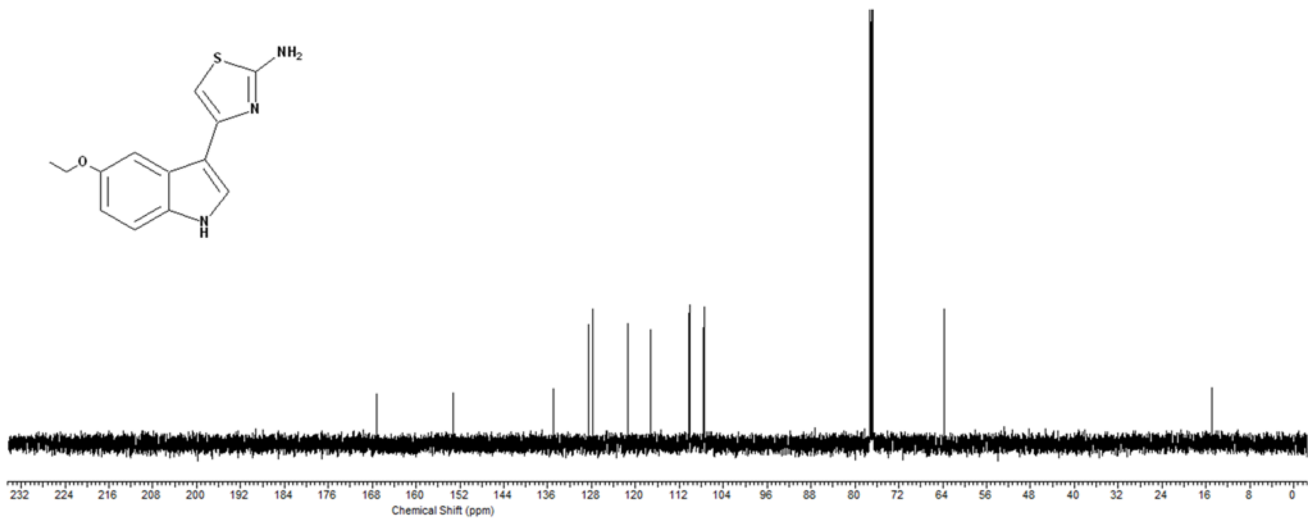
Chemical Shift (ppm)

**Compound 5o**

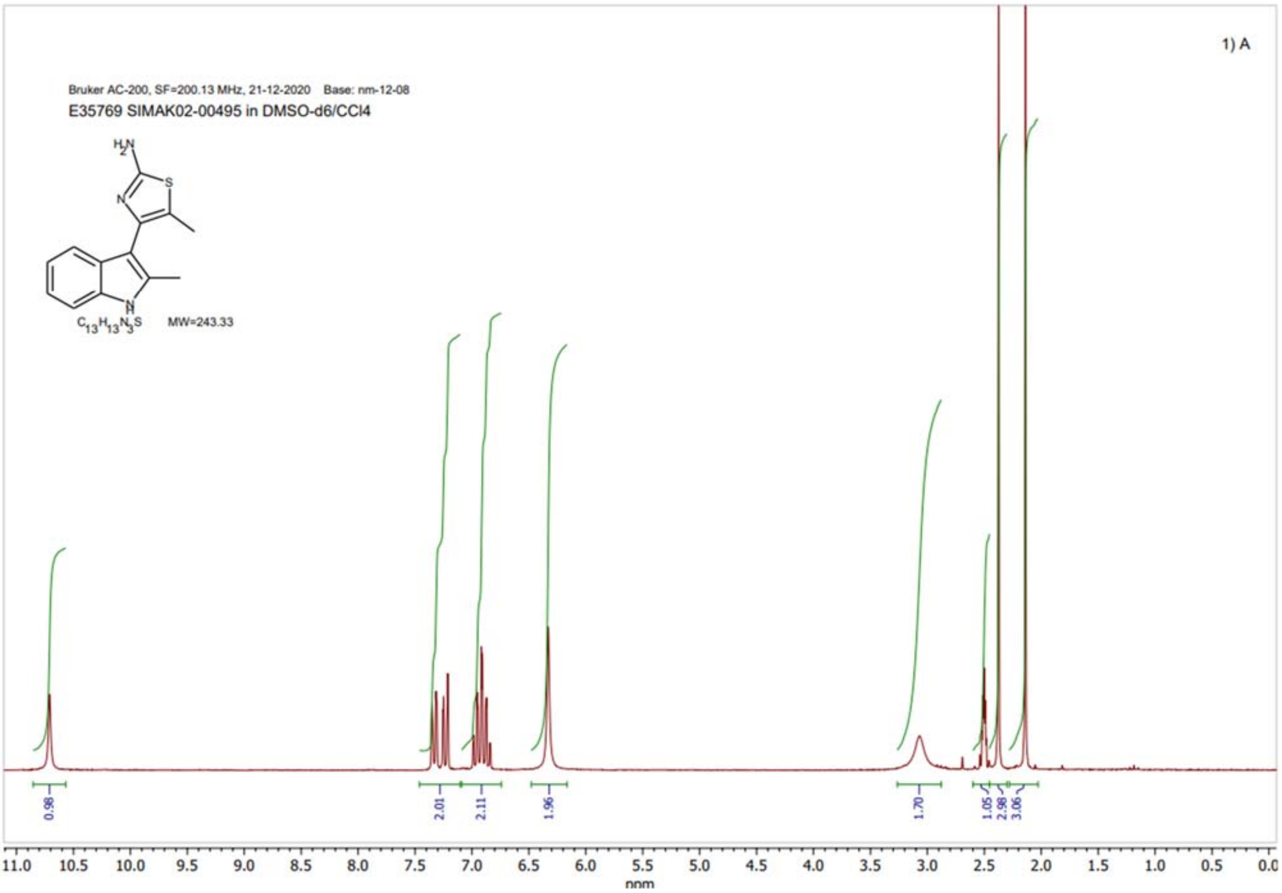
1) A
Bruker AC-200, SF=200.13 MHz, 21-12-2020 Base: nm-12-08
E35769 SIMAK02-00495 in DMSO-d6/CCl4
H2N
S
N
N
C13H13N3S MW=243.33
0.98
2.01
2.11
1.96
1.70
1.05
2.98
3.06
11.0 10.5 10.0 9.5 9.0 8.5 8.0 7.5 7.0 6.5 6.0 5.5 5.0 4.5 4.0 3.5 3.0 2.5 2.0 1.5 1.0 0.5 0.0
ppm

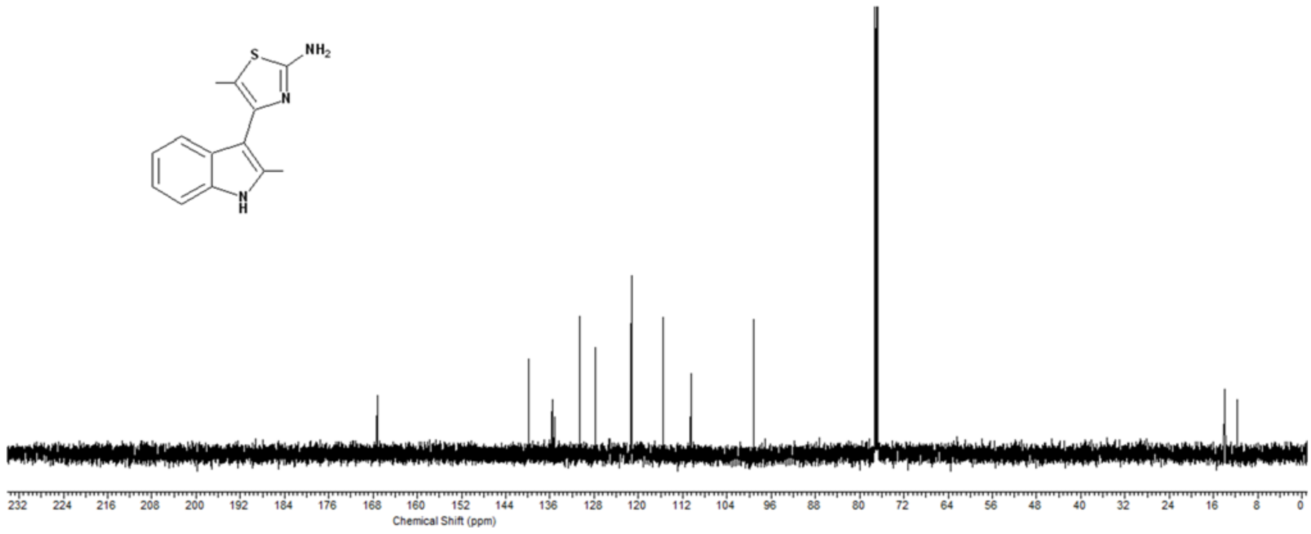
S
NH2
N
H
232 224 216 208 200 192 184 176 168 160 152 144 136 128 120 112 104 96 88 80 72 64 56 48 40 32 24 16 8 0
Chemical Shift (ppm)

**Compound 5p**

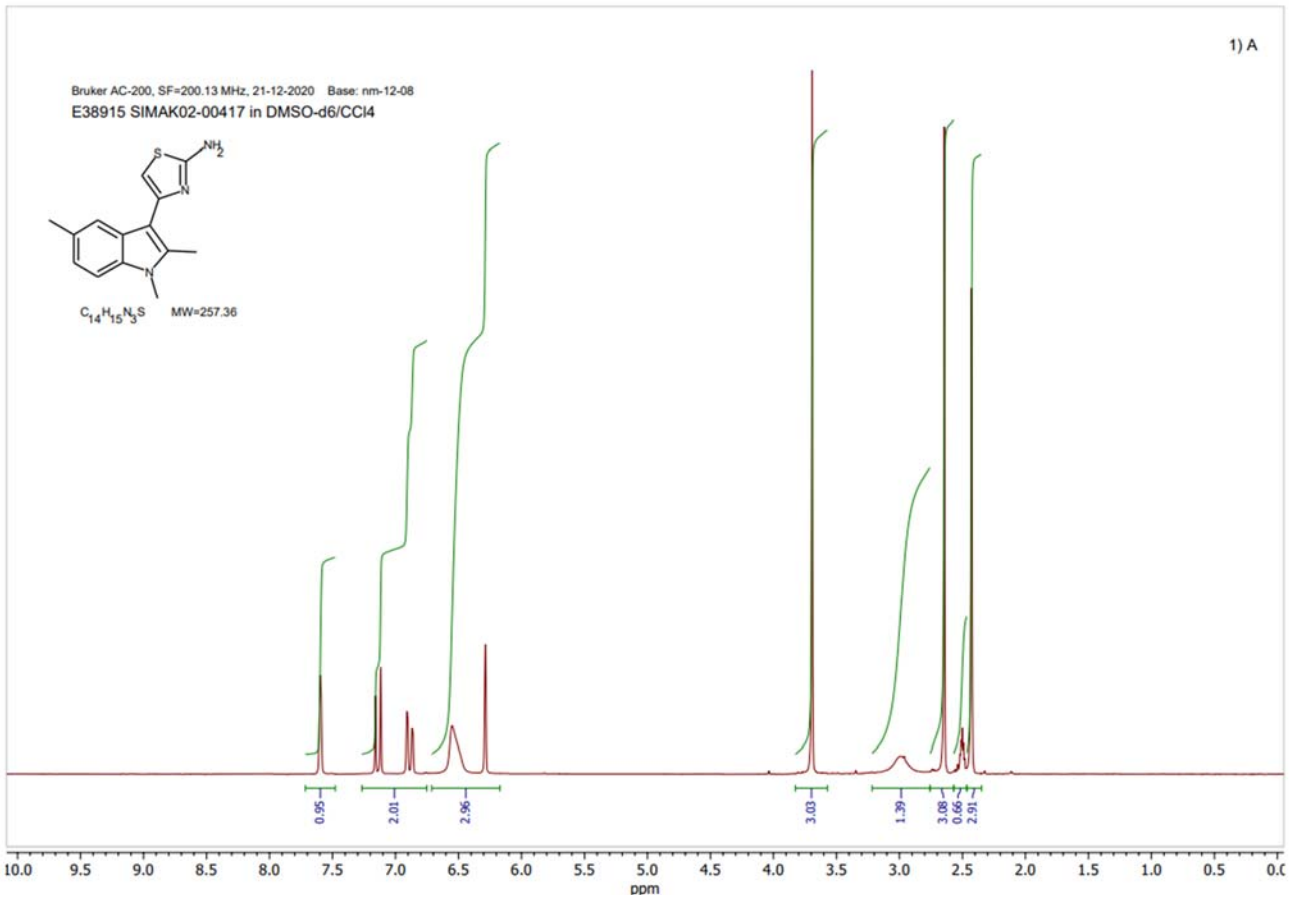
1) A
Bruker AC-200, SF=200.13 MHz, 21-12-2020 Base: nm-12-08
E38915 SIMAK02-00417 in DMSO-d6/CCl4
NH2
S
N
N
C14H15N3S MW=257.36
0.95
2.01
2.96
3.03
1.39
3.08
0.66
2.91
10.0 9.5 9.0 8.5 8.0 7.5 7.0 6.5 6.0 5.5 5.0 4.5 4.0 3.5 3.0 2.5 2.0 1.5 1.0 0.5
ppm

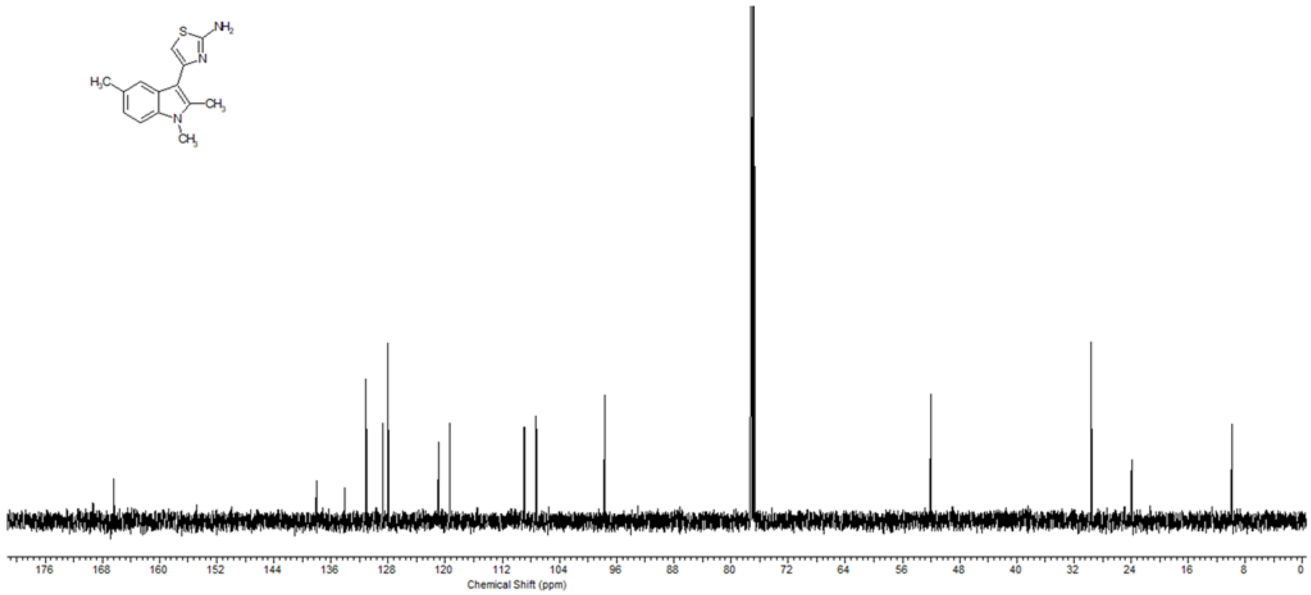
NH2
S
N
H3C
CH3
N
CH3
176 168 160 152 144 136 128 120 112 104 96 88 80 72 64 56 48 40 32 24 16 8 0
Chemical Shift (ppm)

**Compound 5q**

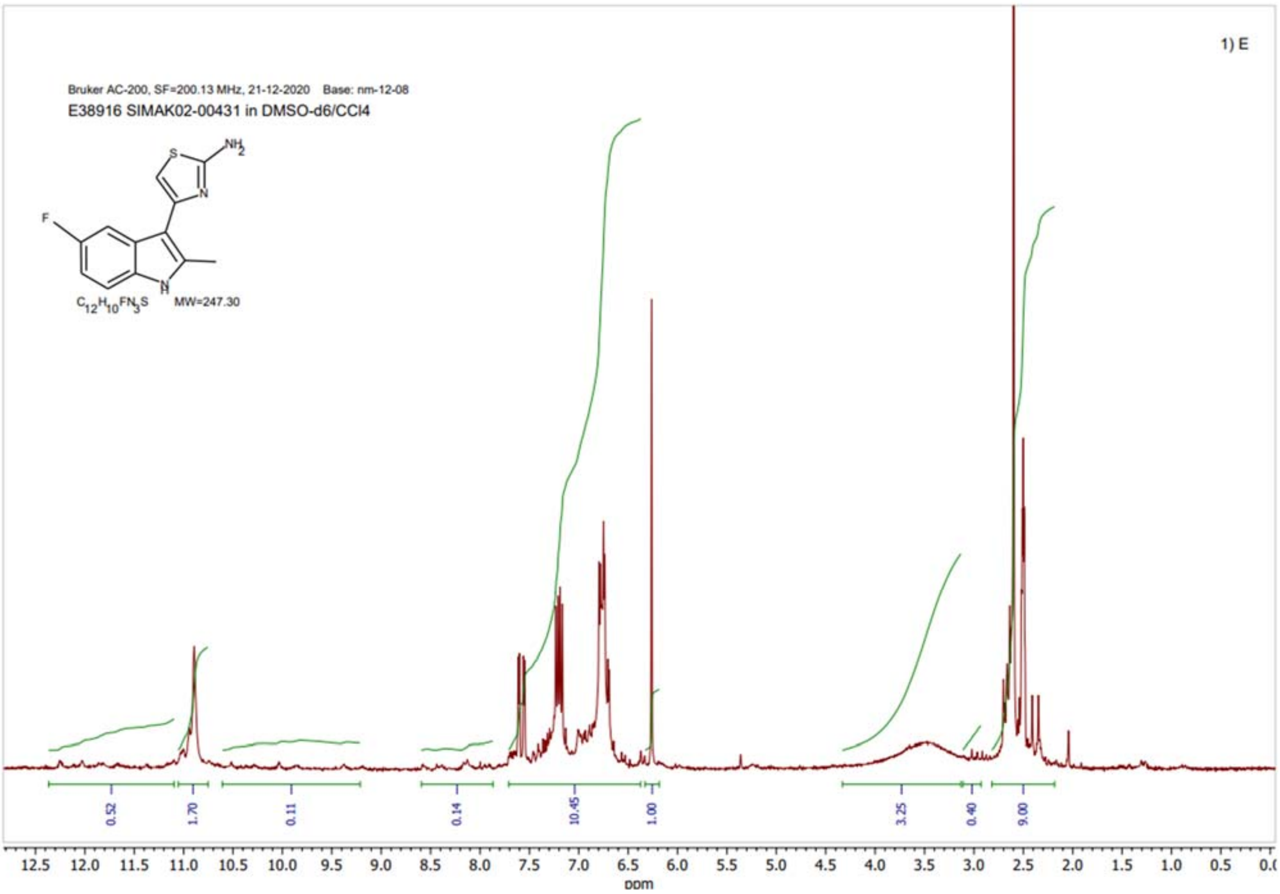

1) E
Bruker AC-200, SF=200.13 MHz, 21-12-2020 Base: nm-12-08
E38916 SIMAK02-00431 in DMSO-d6/CCl4
MW=247.30
0.52
1.70
0.11
0.14
10.45
1.00
3.25
0.40
9.00
12.5 12.0 11.5 11.0 10.5 10.0 9.5 9.0 8.5 8.0 7.5 7.0 6.5 6.0 5.5 5.0 4.5 4.0 3.5 3.0 2.5 2.0 1.5 1.0 0.5
ppm


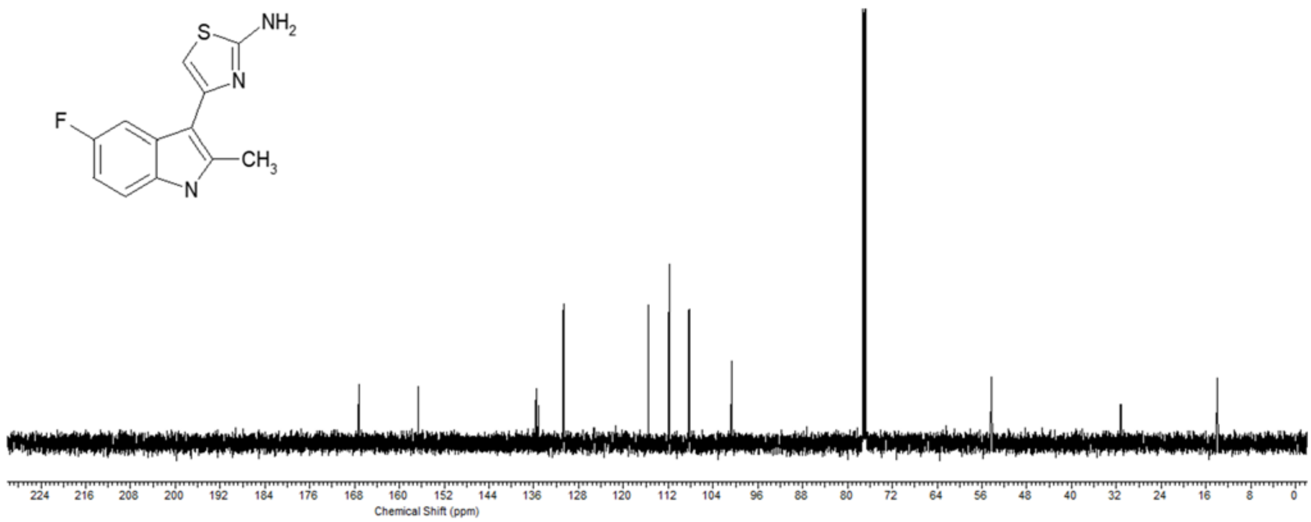

224 216 208 200 192 184 176 168 160 152 144 136 128 120 112 104 96 88 80 72 64 56 48 40 32 24 16 8 0
Chemical Shift (ppm)


**Compound 5r**

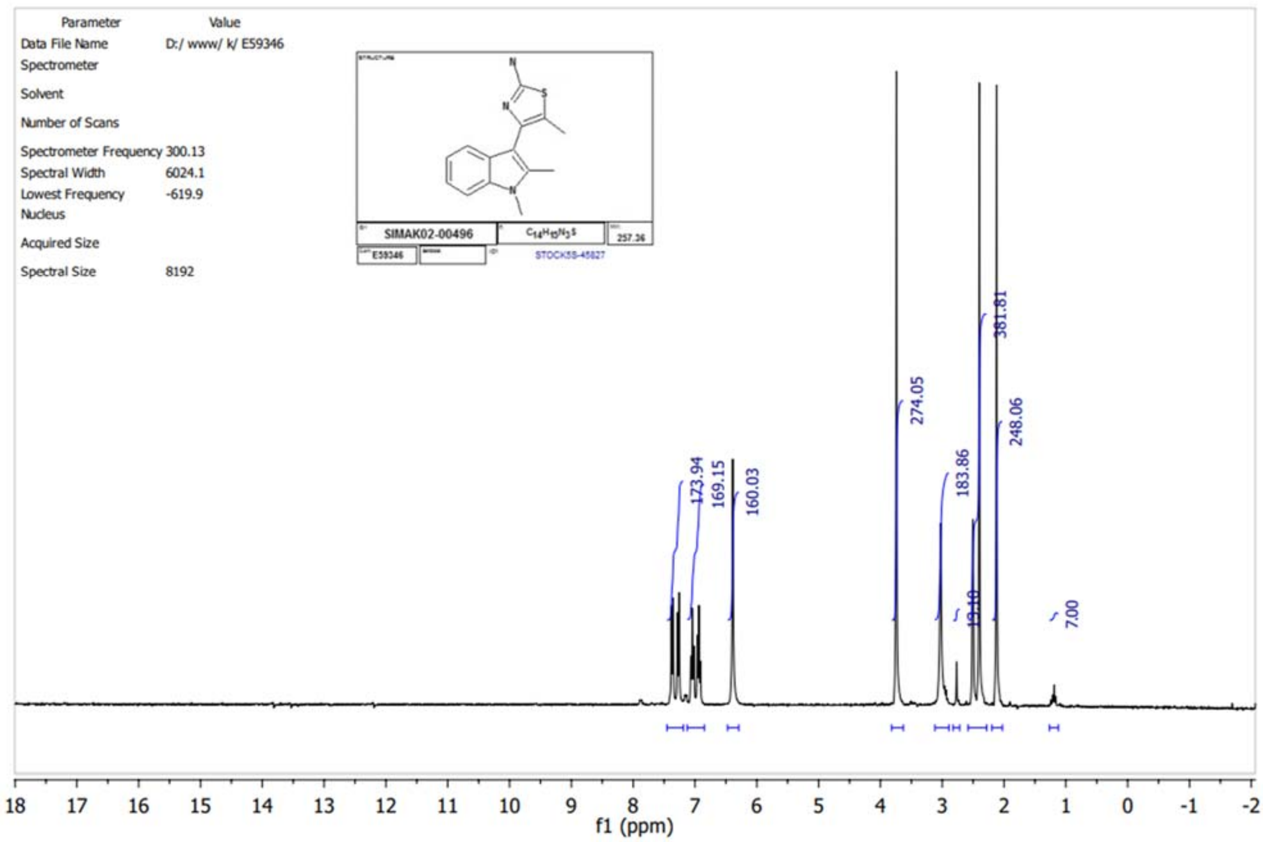
Parameter
Value
Data File Name
D:/ www/ k/ E59346
Spectrometer
Solvent
Number of Scans
Spectrometer Frequency 300.13
Spectral Width
6024.1
Lowest Frequency
-619.9
Nucleus
Acquired Size
Spectral Size
8192
SIMAK02-00496
257.36
E59346
173.94
169.15
160.03
274.05
183.86
381.81
248.06
7.00
f1 (ppm)

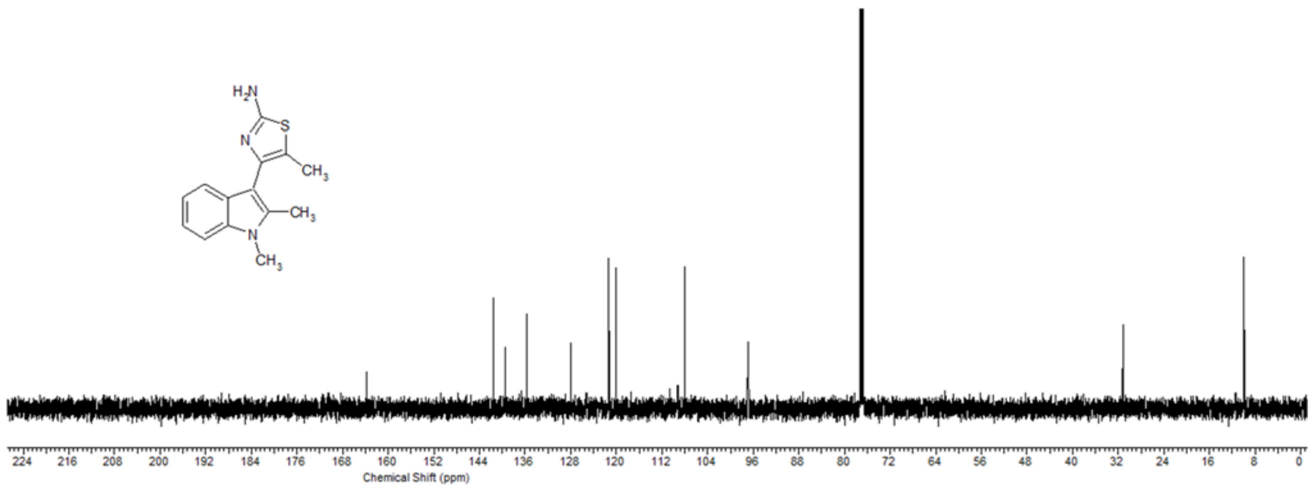
H2N
N
S
CH3
CH3
N
CH3
Chemical Shift (ppm)

**Compound 5s**

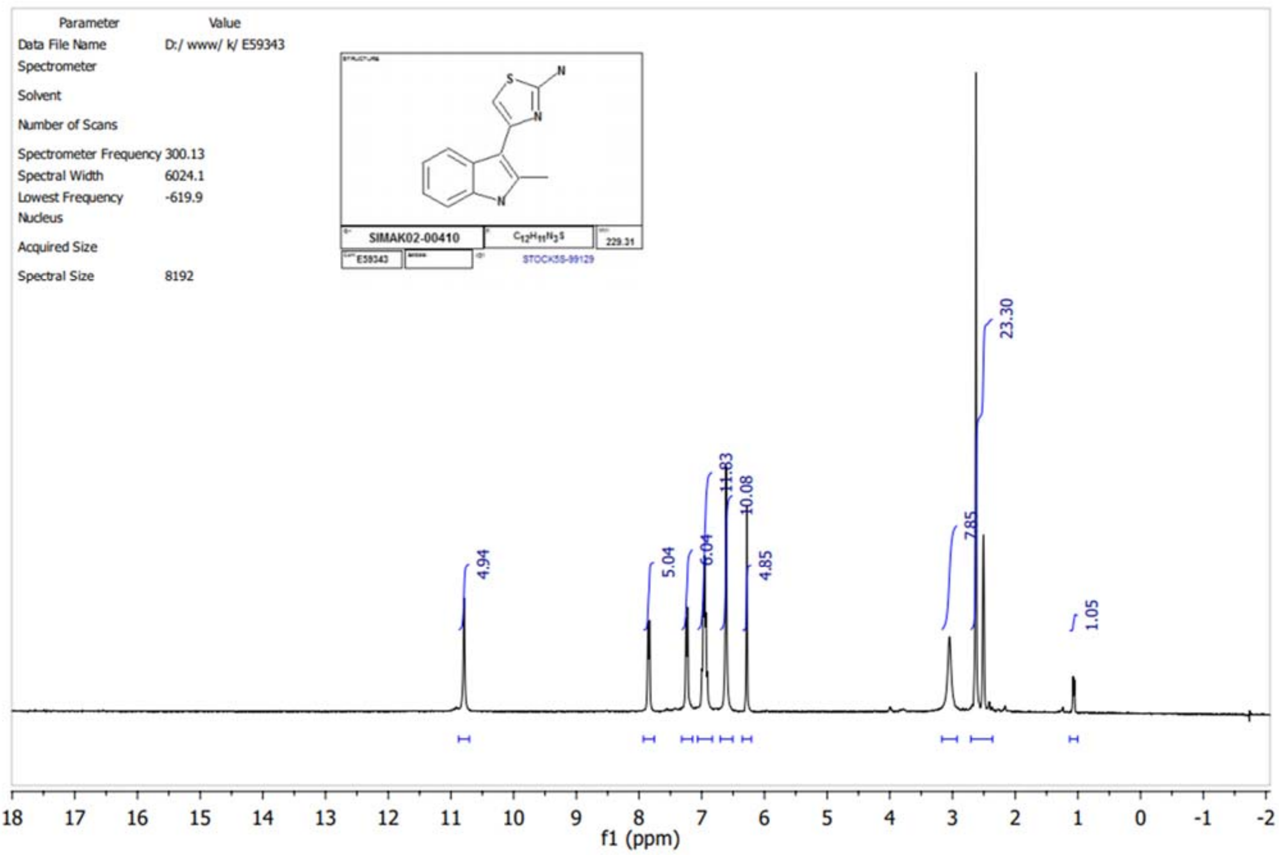
Parameter
Value
Data File Name
D:/ www/ k/ E59343
Spectrometer
Solvent
Number of Scans
Spectrometer Frequency 300.13
Spectral Width
6024.1
Lowest Frequency
-619.9
Nucleus
Acquired Size
Spectral Size
8192
SIMAK02-00410
229.31
E59343
4.94
5.04
6.04
11.83
10.08
4.85
7.85
23.30
1.05
f1 (ppm)

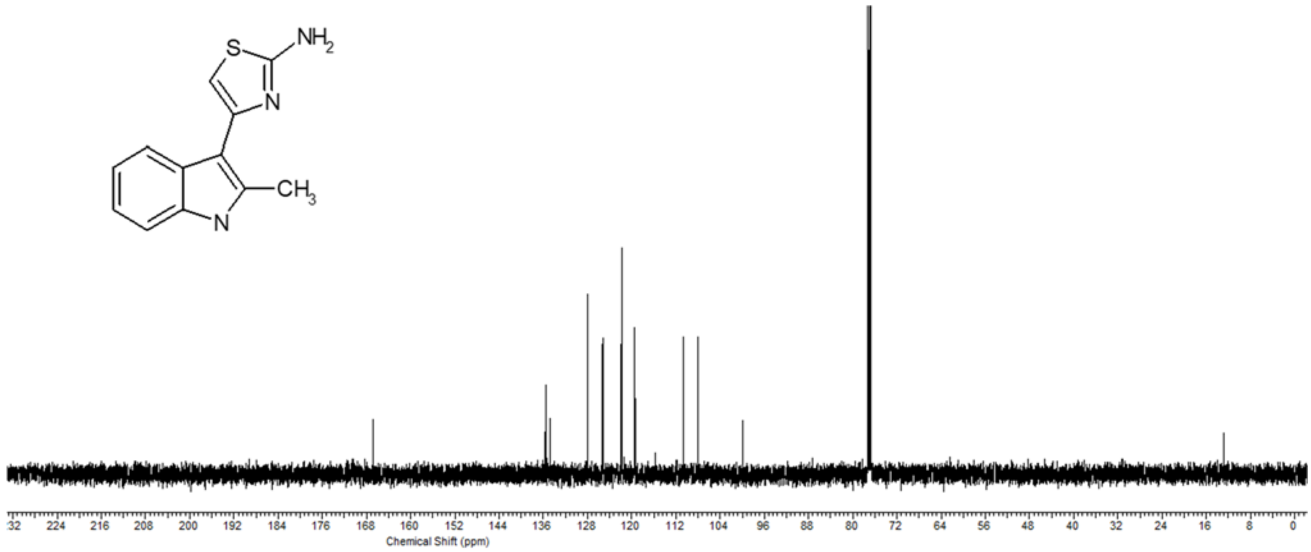
NH2
S
N
N
CH3
Chemical Shift (ppm)

**Compound 5t**

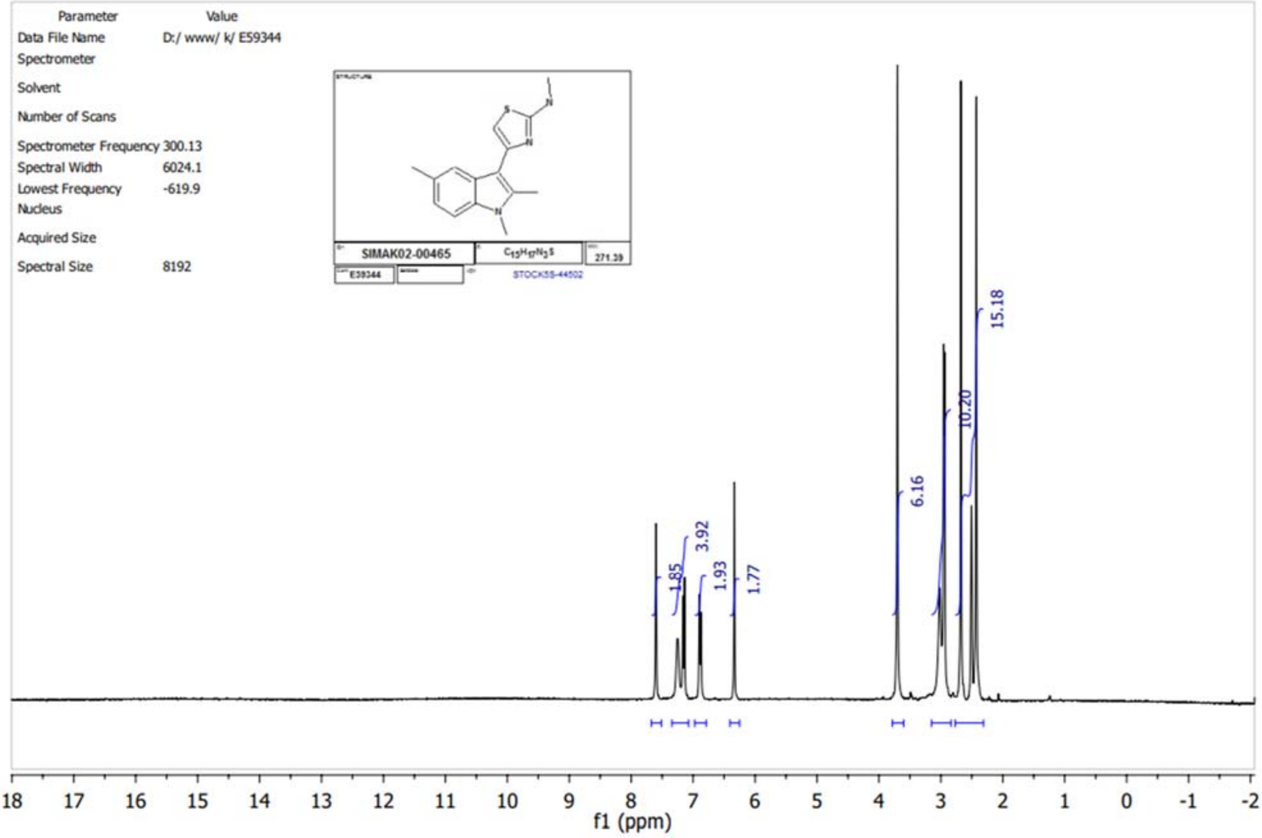

Parameter Value
Data File Name D:/ www/ k/ E59344
Spectrometer
Solvent
Number of Scans
Spectrometer Frequency 300.13
Spectral Width 6024.1
Lowest Frequency -619.9
Nucleus
Acquired Size
Spectral Size 8192
SIMAK02-00465
271.39
E59344
STOCK5S-44502
6.16
10.20
15.18
1.85
3.92
1.93
1.77
18 17 16 15 14 13 12 11 10 9 8 7 6 5 4 3 2 1 0 -1 -2
f1 (ppm)


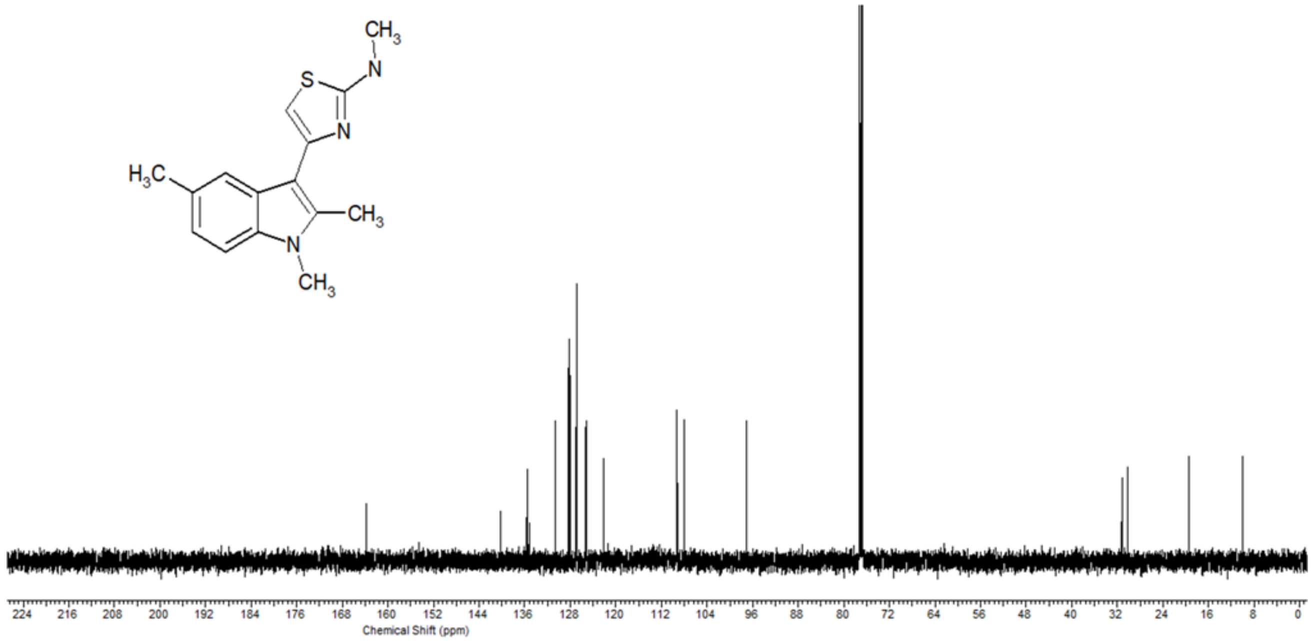

CH3
N
S
N
H3C
CH3
N
CH3
224 216 208 200 192 184 176 168 160 152 144 136 128 120 112 104 96 88 80 72 64 56 48 40 32 24 16 8 0
Chemical Shift (ppm)


**Compound 5u**

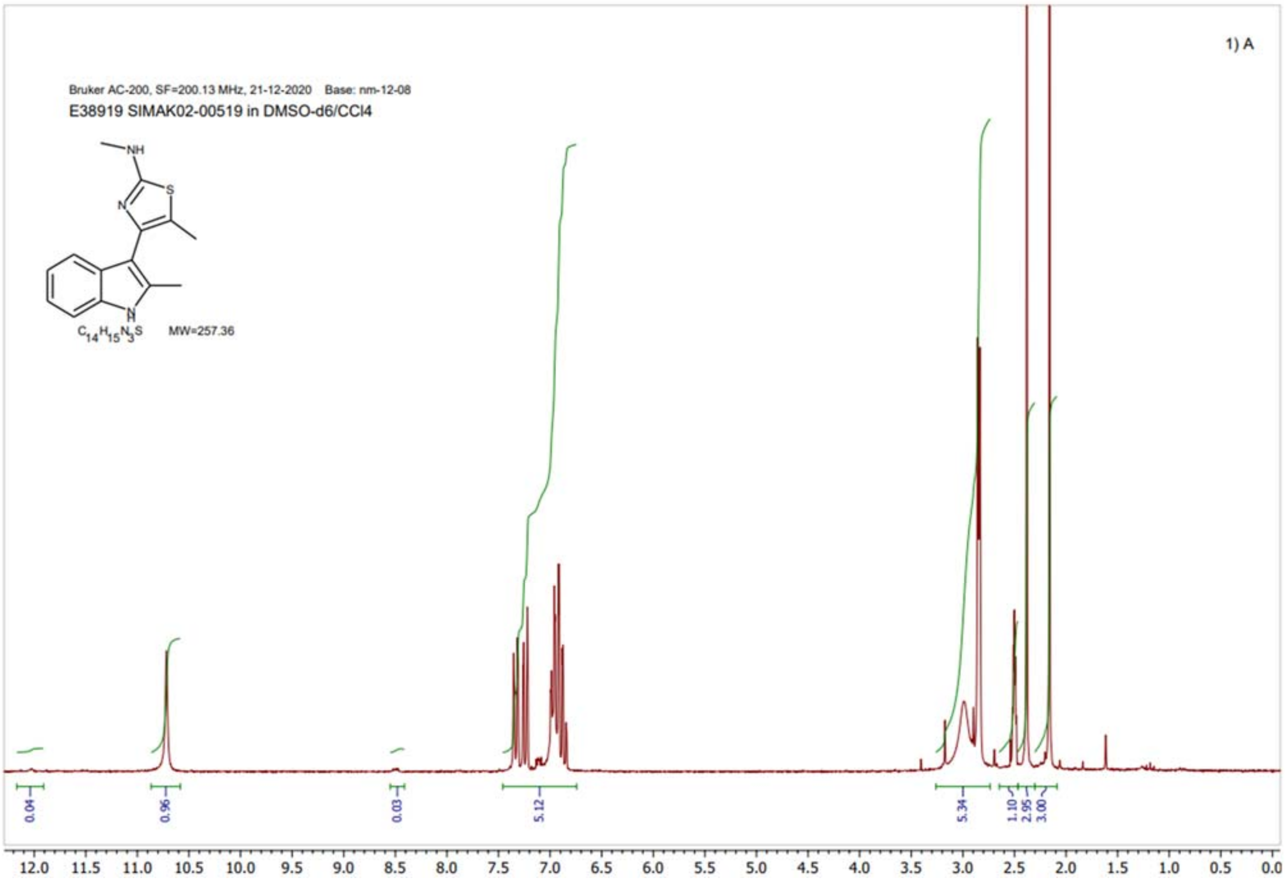
1) A
Bruker AC-200, SF=200.13 MHz, 21-12-2020 Base: nm-12-08
E38919 SIMAK02-00519 in DMSO-d6/CCl4
MW=257.36

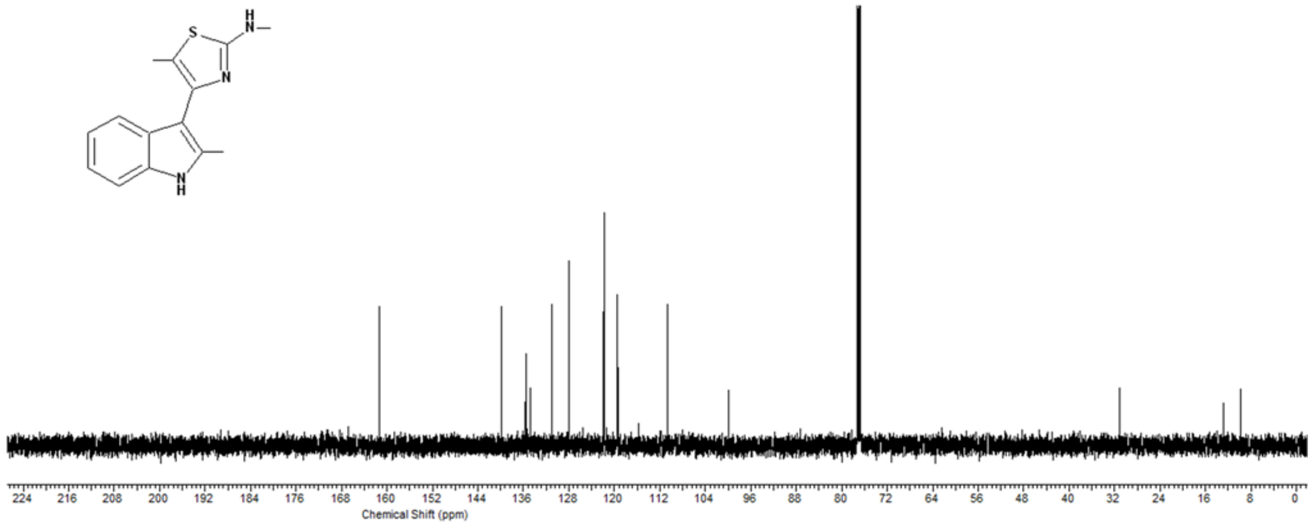
Chemical Shift (ppm)

**Compound 5V**

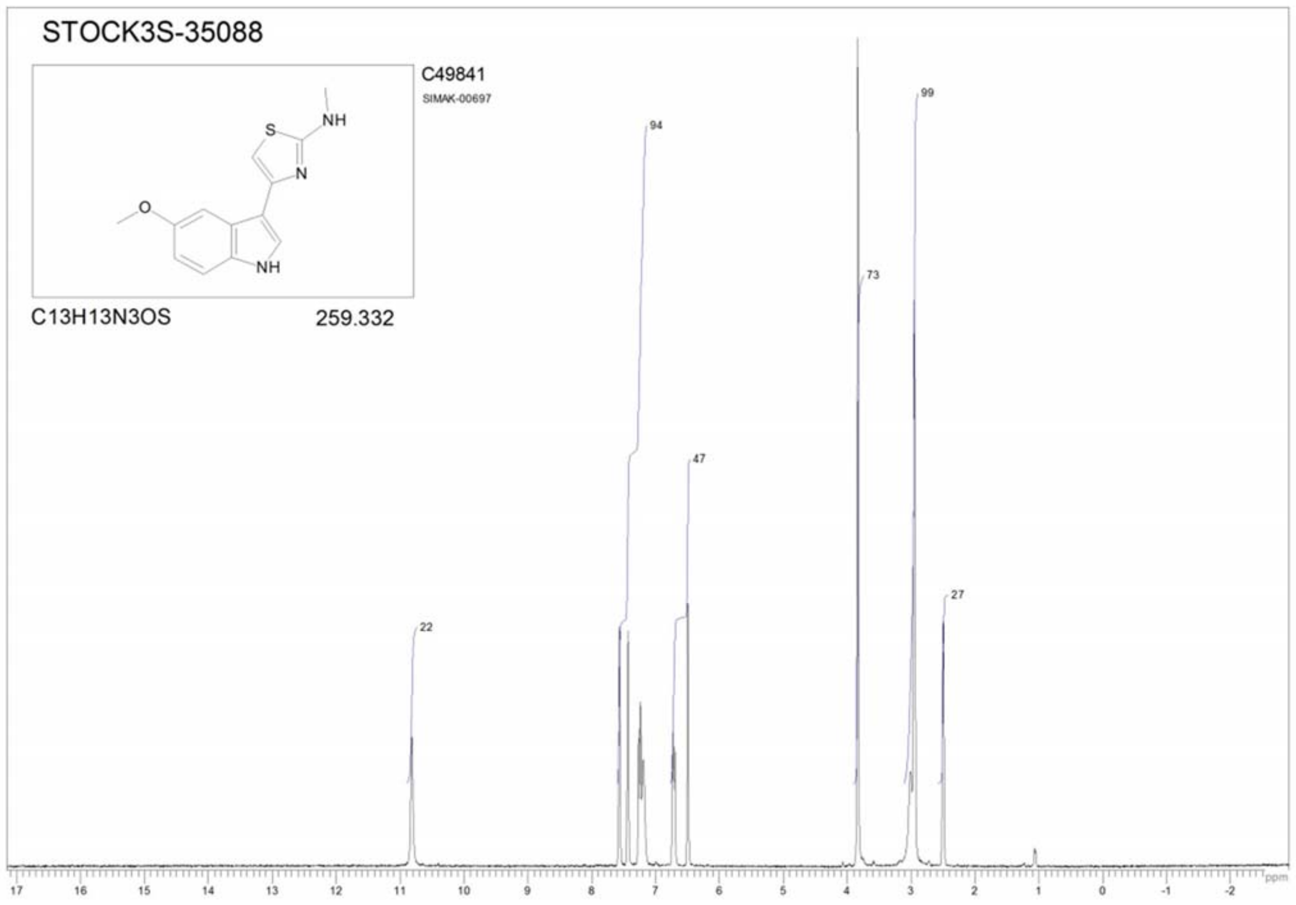
STOCK3S-35088
C49841
SIMAK-00697
C13H13N3OS
259.332
ppm

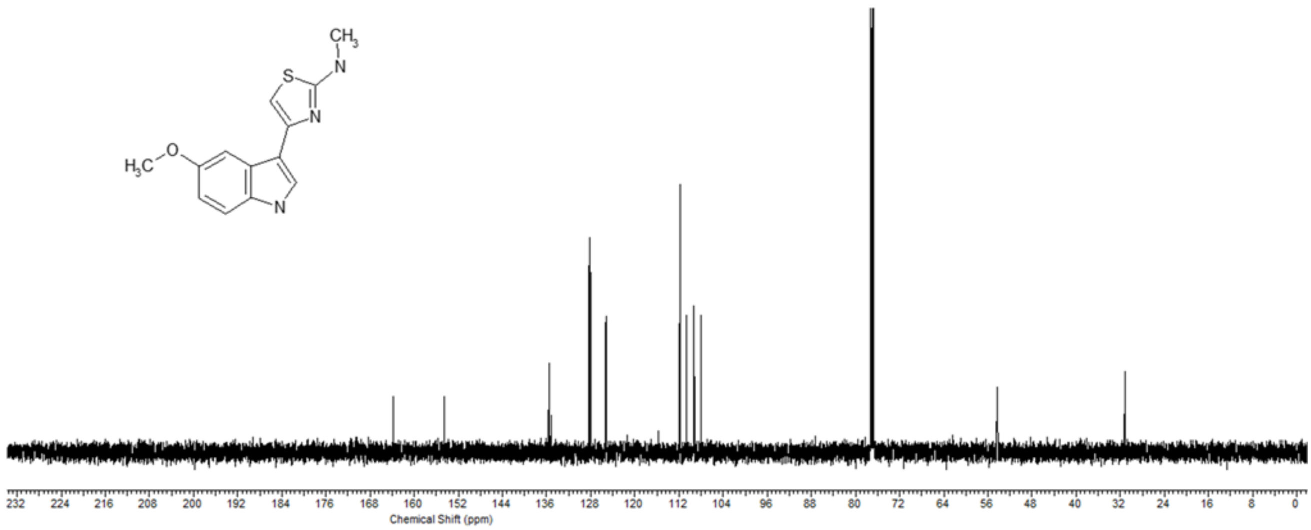
Chemical Shift (ppm)

**Compound 5W**

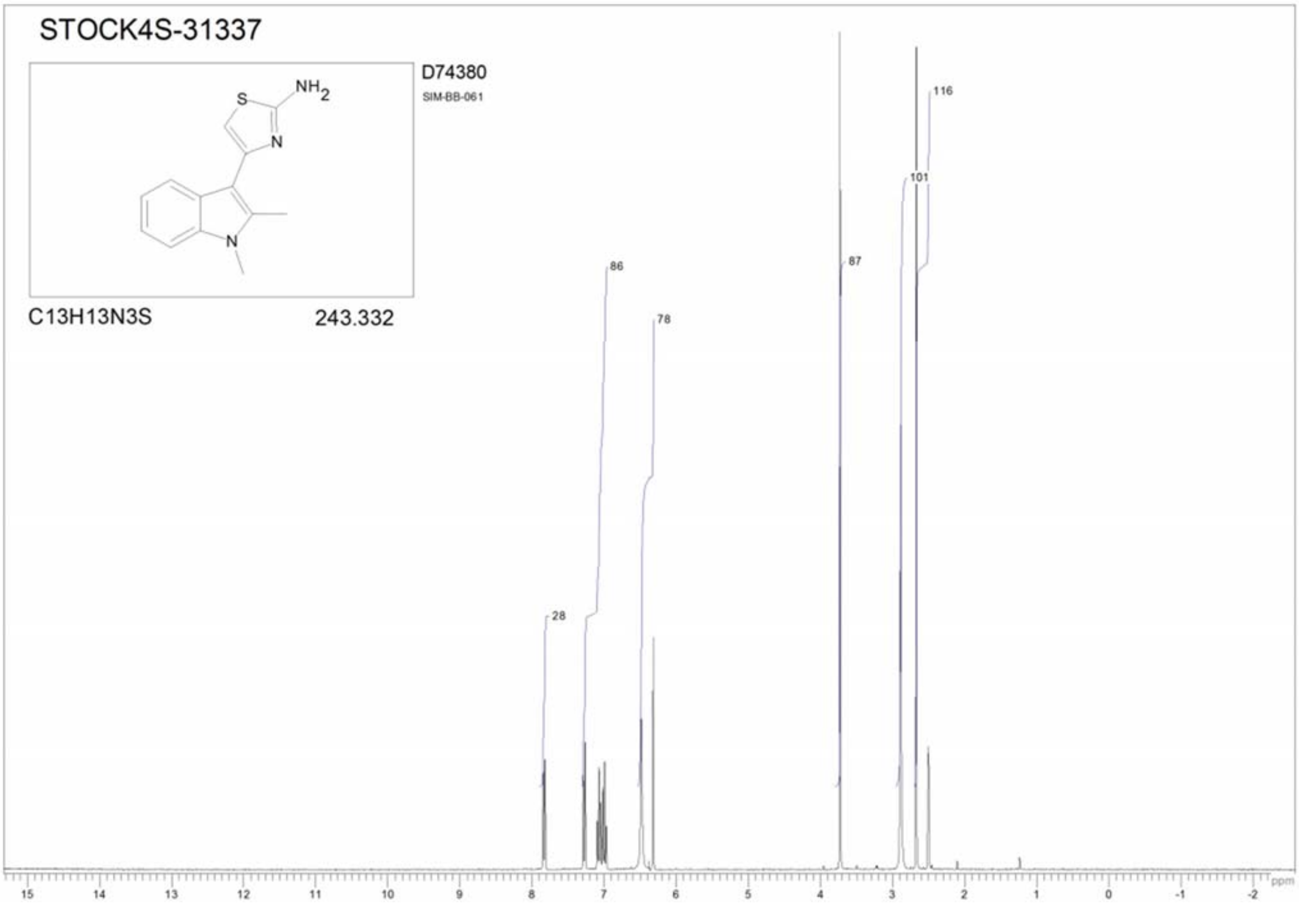
STOCK4S-31337
D74380
SIM-BB-061
C13H13N3S
243.332
ppm

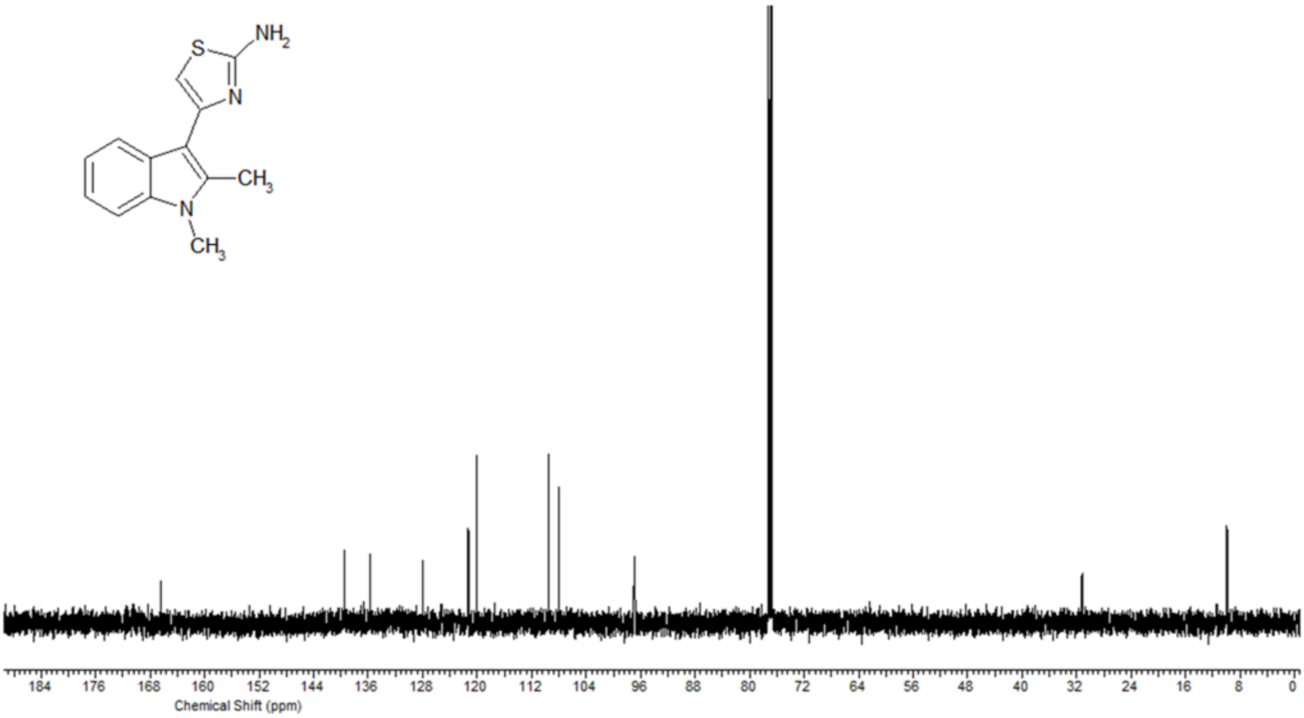
Chemical Shift (ppm)

**Compound 5X**

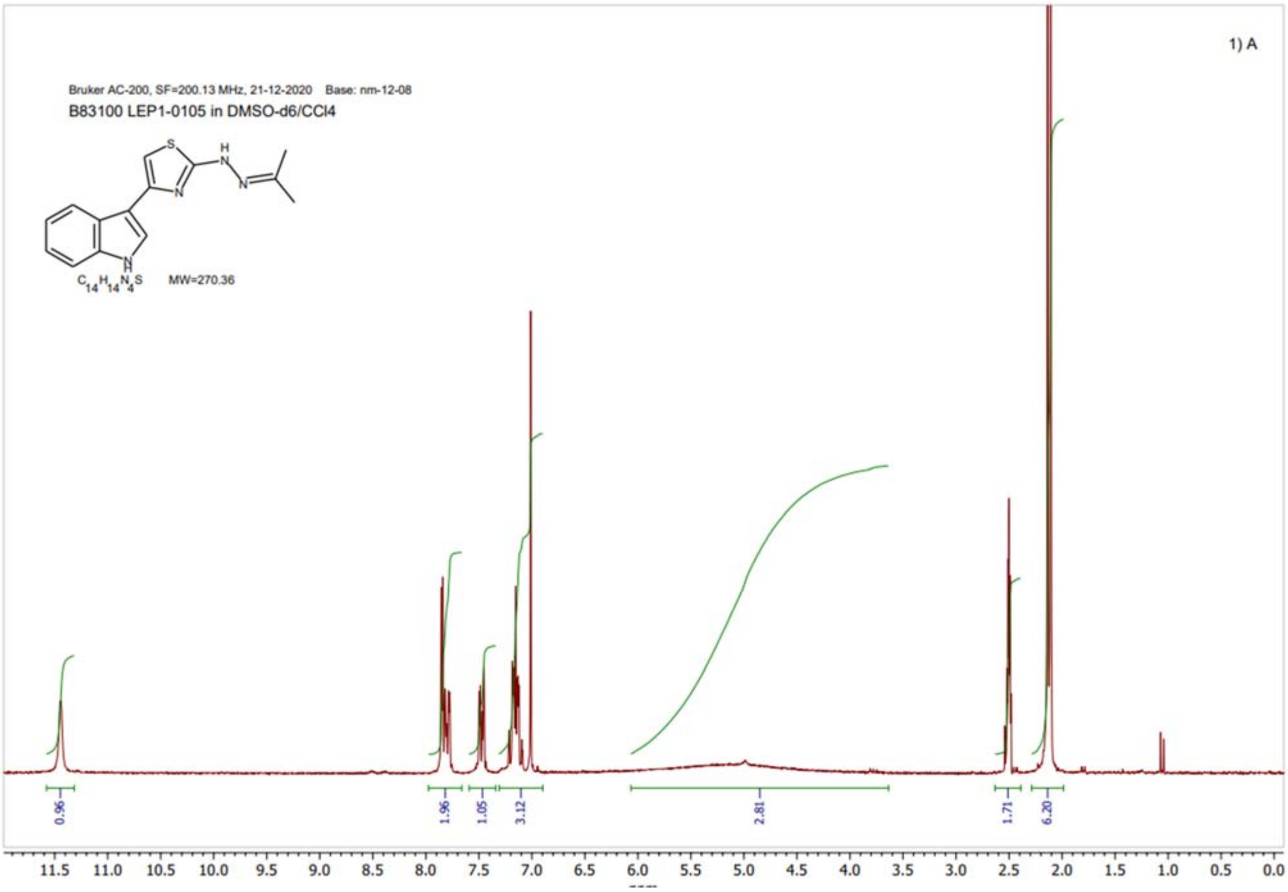
1) A
Bruker AC-200, SF=200.13 MHz, 21-12-2020 Base: nm-12-08
B83100 LEP1-0105 in DMSO-d6/CCl4
$C_{14}H_{14}N_4S$ MW=270.36
0.96
1.96
1.05
3.12
2.81
1.71
6.20
11.5 11.0 10.5 10.0 9.5 9.0 8.5 8.0 7.5 7.0 6.5 6.0 5.5 5.0 4.5 4.0 3.5 3.0 2.5 2.0 1.5 1.0 0.5 0.0

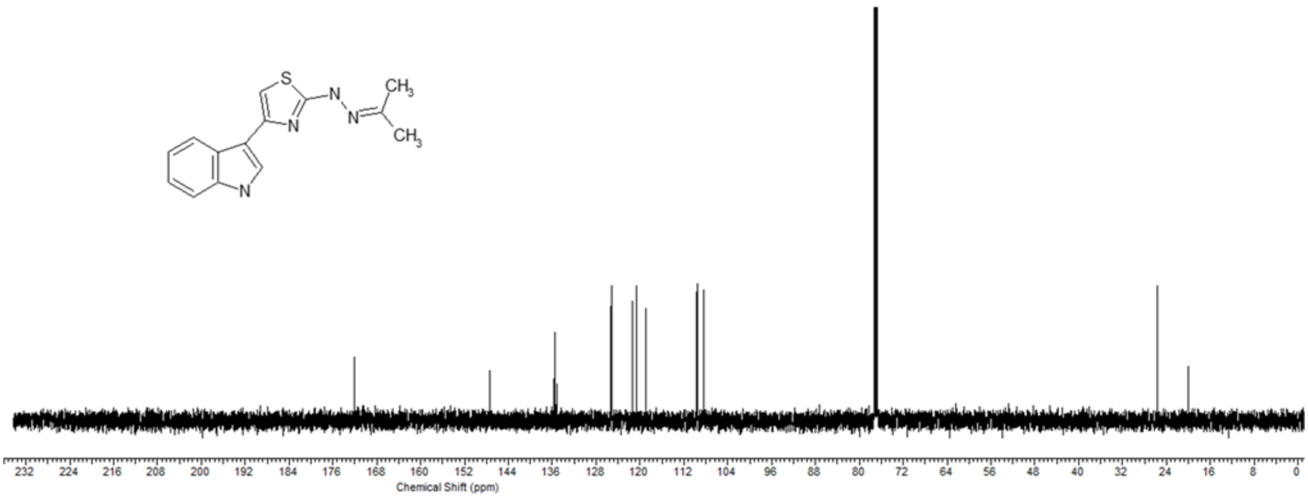
232 224 216 208 200 192 184 176 168 160 152 144 136 128 120 112 104 96 88 80 72 64 56 48 40 32 24 16 8 0
Chemical Shift (ppm)

**Compound 6a**

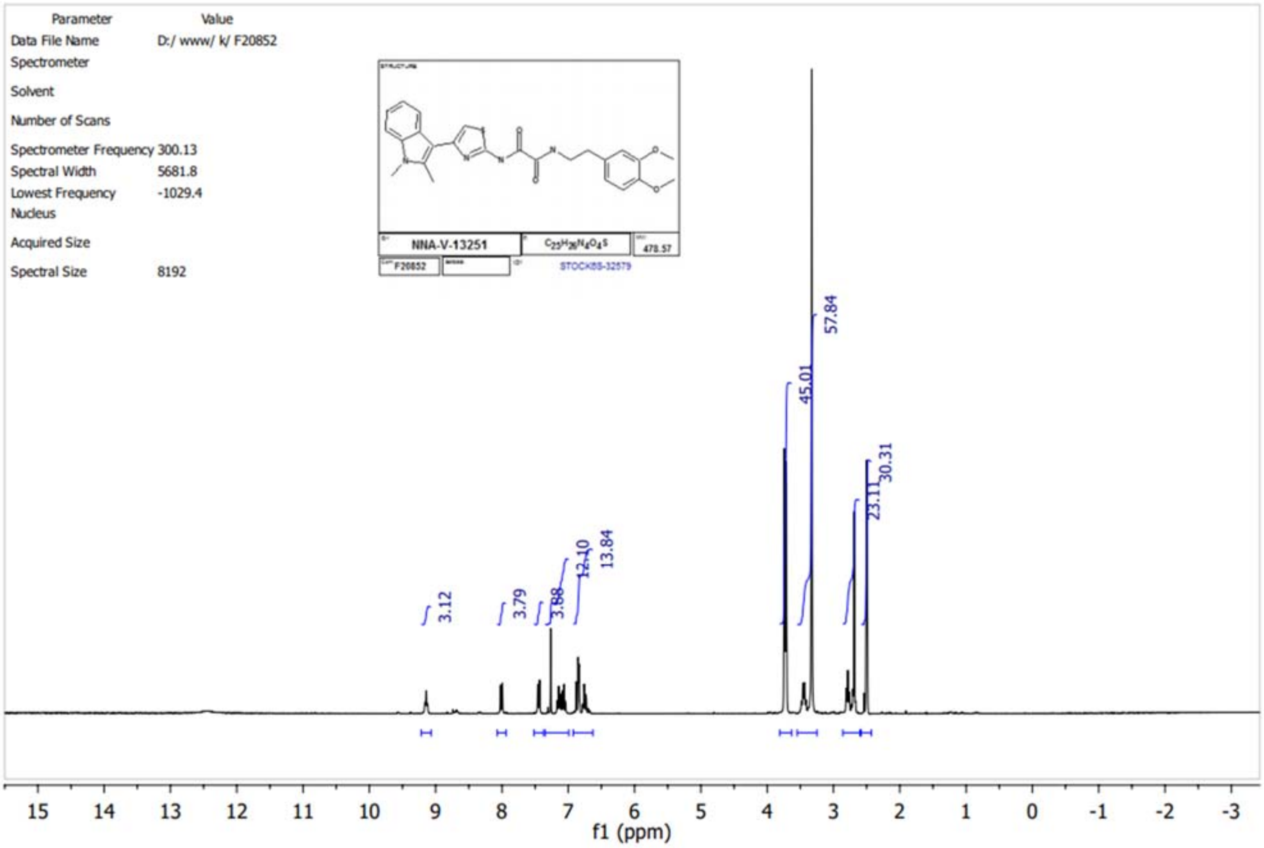
Parameter
Value
Data File Name
D:/ www/ k/ F20852
Spectrometer
Solvent
Number of Scans
Spectrometer Frequency 300.13
Spectral Width
5681.8
Lowest Frequency
-1029.4
Nucleus
Acquired Size
Spectral Size
8192
NNA-V-13251
F20852
STOCKBB-32579
478.57
3.12
3.79
3.88
12.10
13.84
45.01
57.84
23.11
30.31
f1 (ppm)

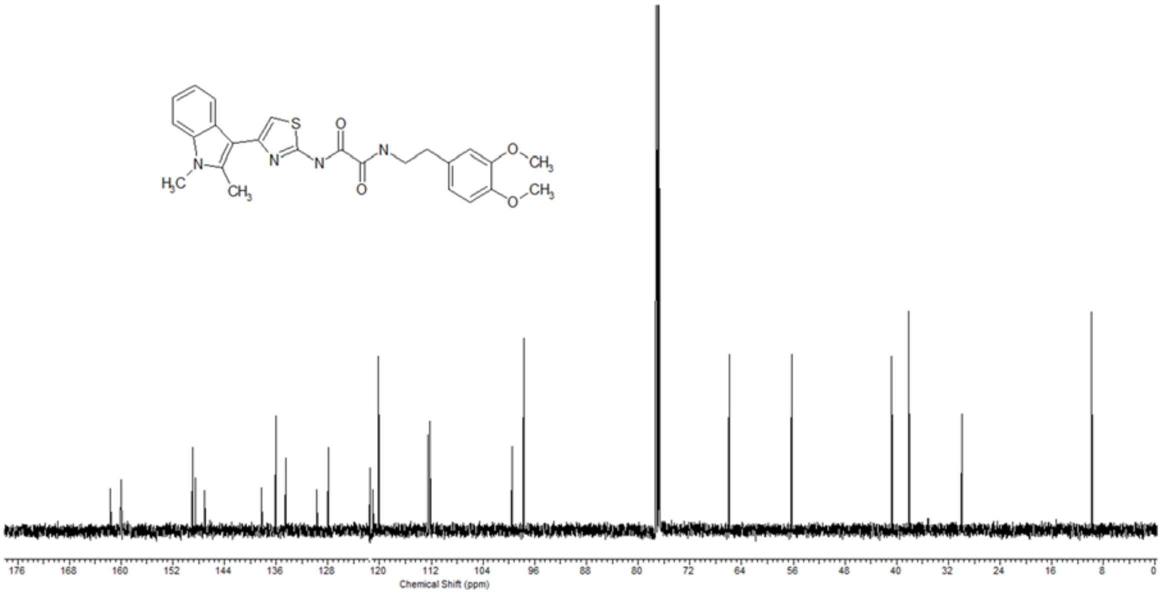
S
N
N
O
O
N
O
CH3
O
CH3
H3C
CH3
Chemical Shift (ppm)

**Compound 6b**

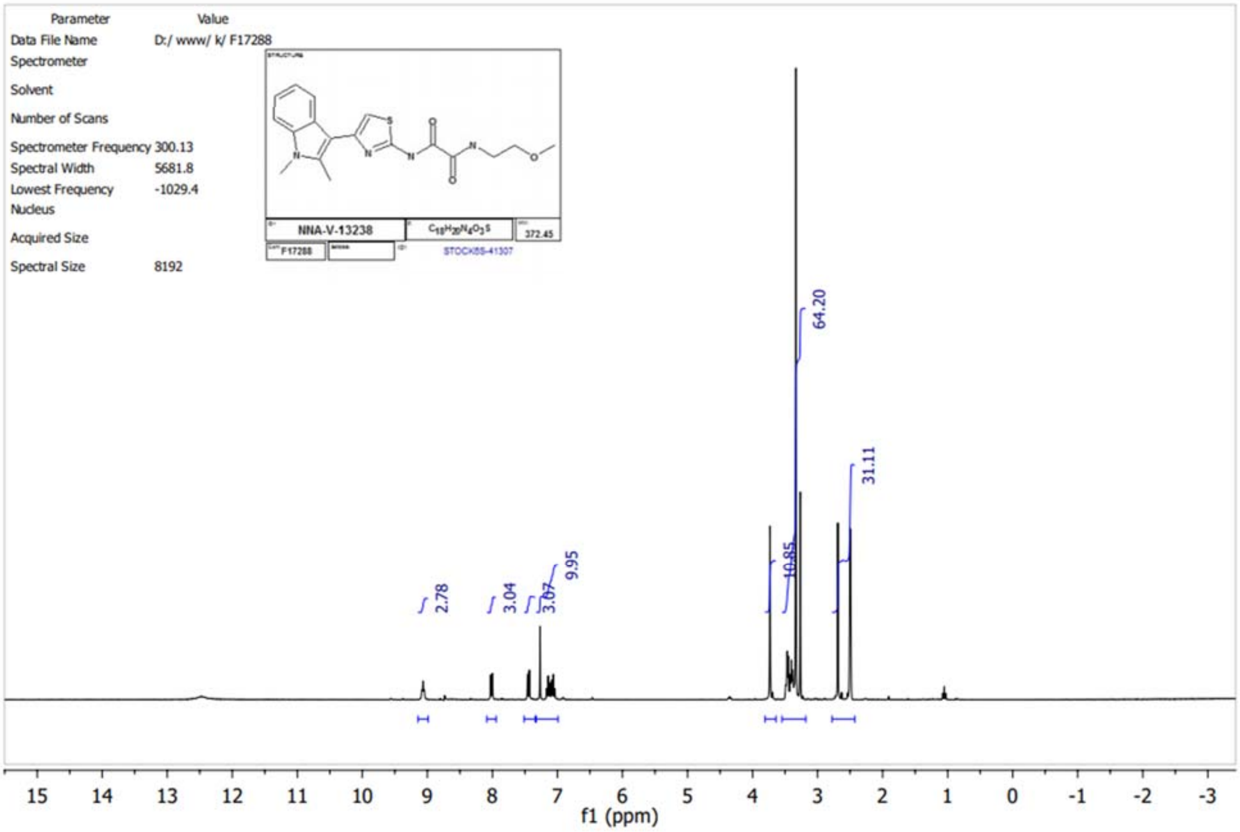
Parameter
Value
Data File Name
D:/ www/ k/ F17288
Spectrometer
Solvent
Number of Scans
Spectrometer Frequency 300.13
Spectral Width 5681.8
Lowest Frequency -1029.4
Nucleus
Acquired Size
Spectral Size 8192
NNA-V-13238
C18H20N4O3S
372.45
F17288
STOCKBS-41307
2.78
3.04
3.07
9.95
10.85
64.20
31.11
15 14 13 12 11 10 9 8 7 6 5 4 3 2 1 0 -1 -2 -3
f1 (ppm)

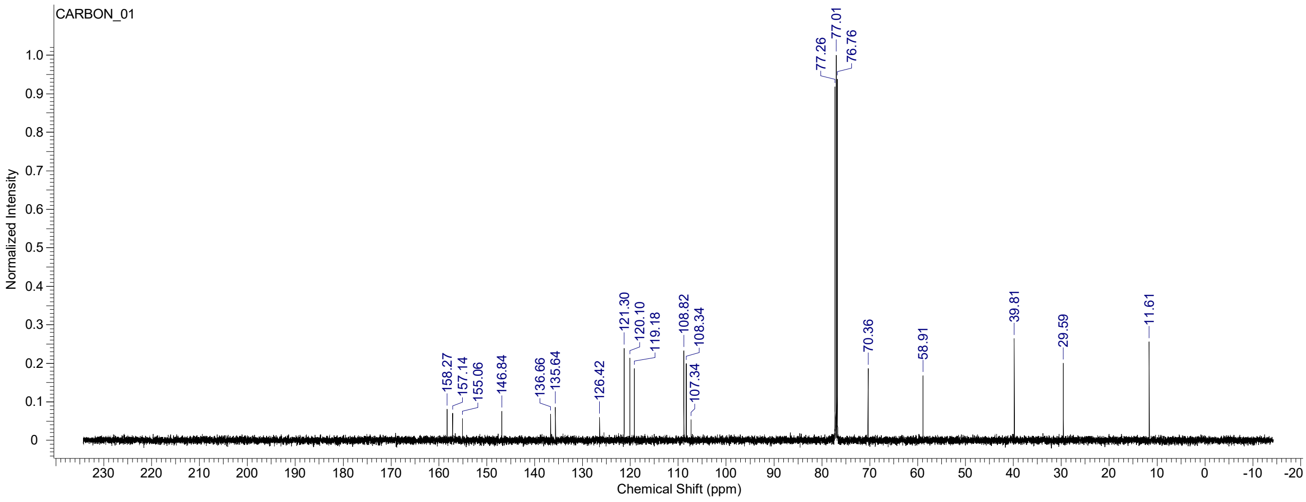
CARBON_01
Normalized Intensity
1.0 0.9 0.8 0.7 0.6 0.5 0.4 0.3 0.2 0.1 0
158.27
157.14
155.06
146.84
136.66
135.64
126.42
121.30
120.10
119.18
108.82
108.34
107.34
77.26
77.01
76.76
70.36
58.91
39.81
29.59
11.61
230 220 210 200 190 180 170 160 150 140 130 120 110 100 90 80 70 60 50 40 30 20 10 0 -10 -20
Chemical Shift (ppm)

**Compound 6c**

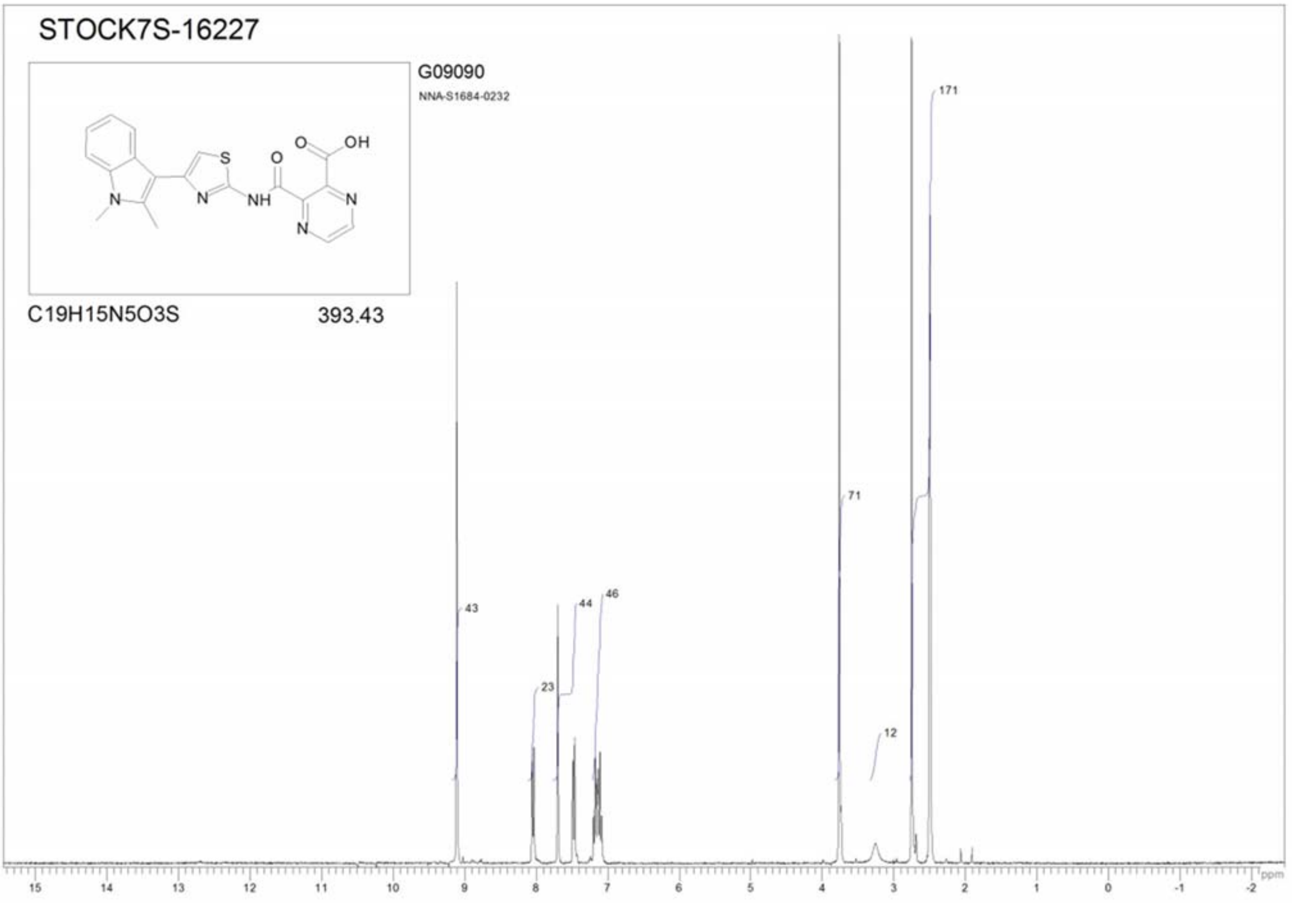
STOCK7S-16227
G09090
NNA-S1684-0232
C19H15N5O3S
393.43
171
71
43
44
46
23
12
ppm

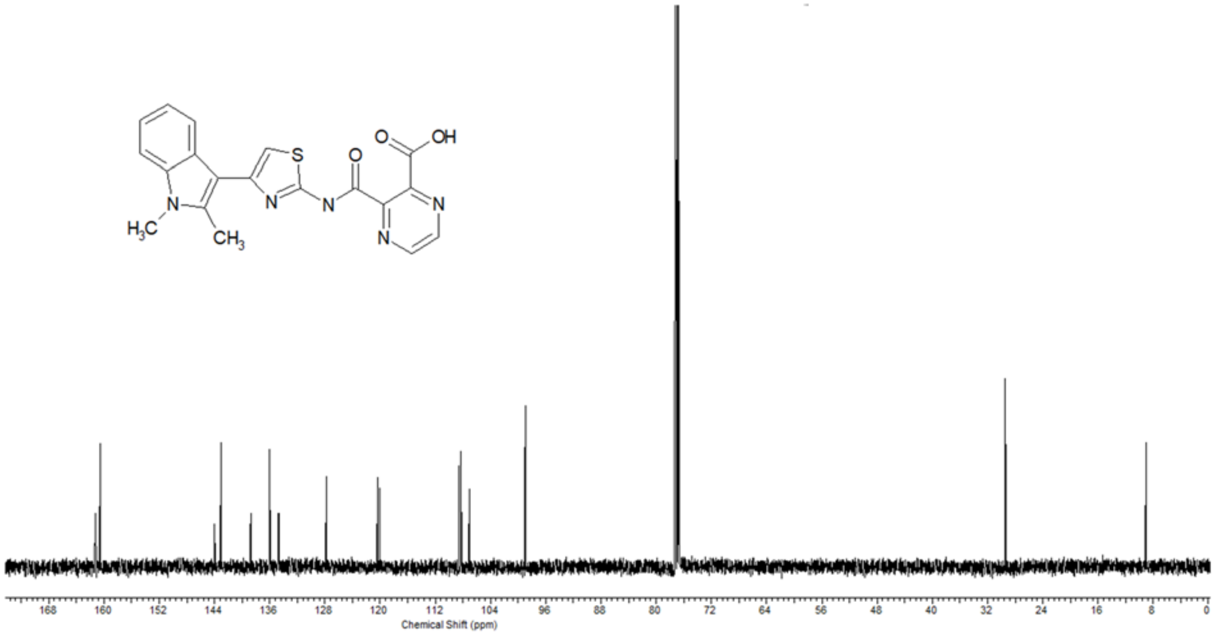
Chemical Shift (ppm)

**Compound 6d**

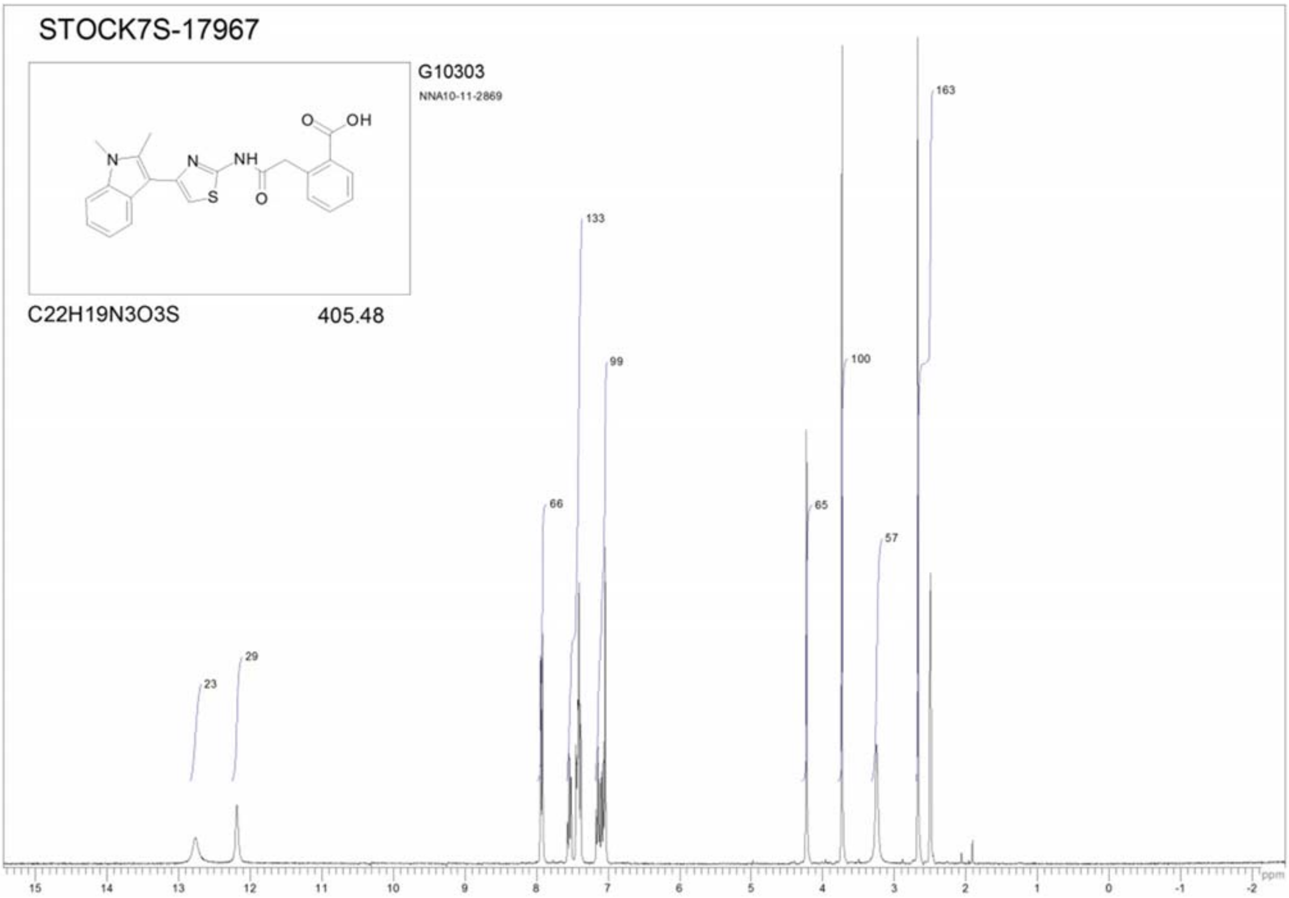

STOCK7S-17967
G10303
NNA10-11-2869
C22H19N3O3S
405.48


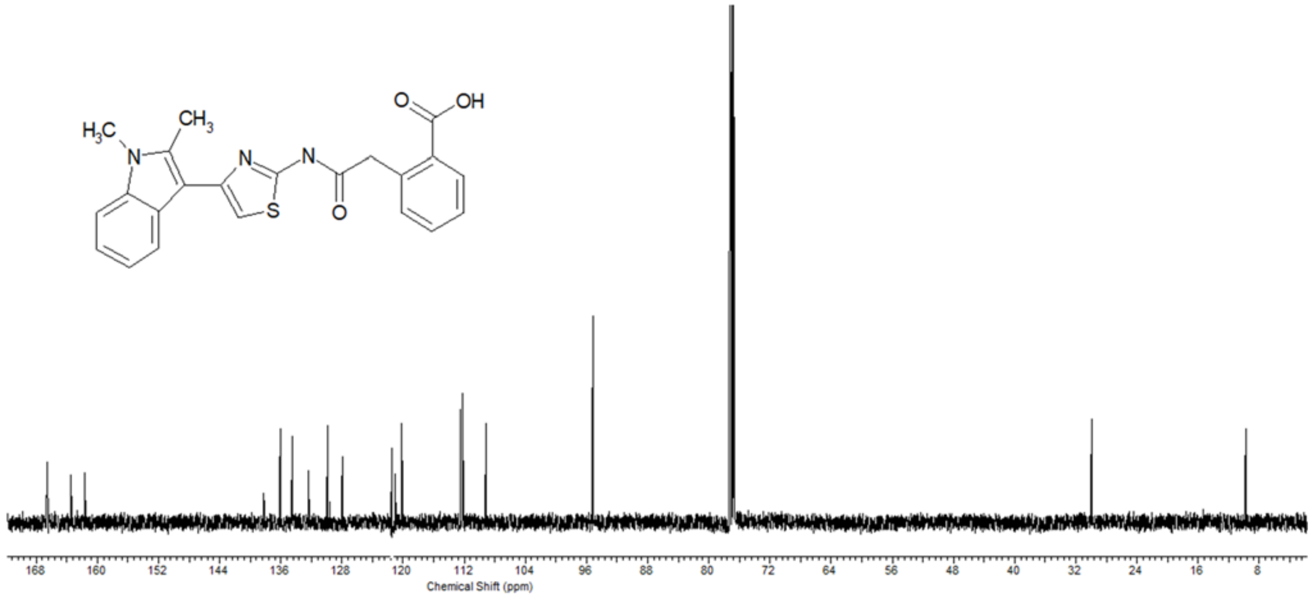

Chemical Shift (ppm)


**Compound 6e**

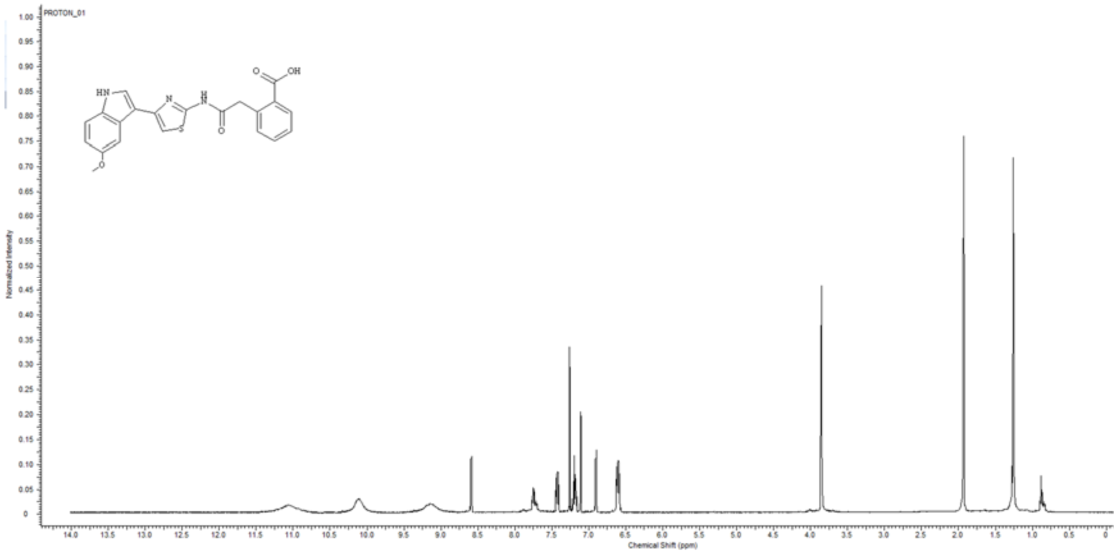
PROTON_01
Normalized Intensity
Chemical Shift (ppm)

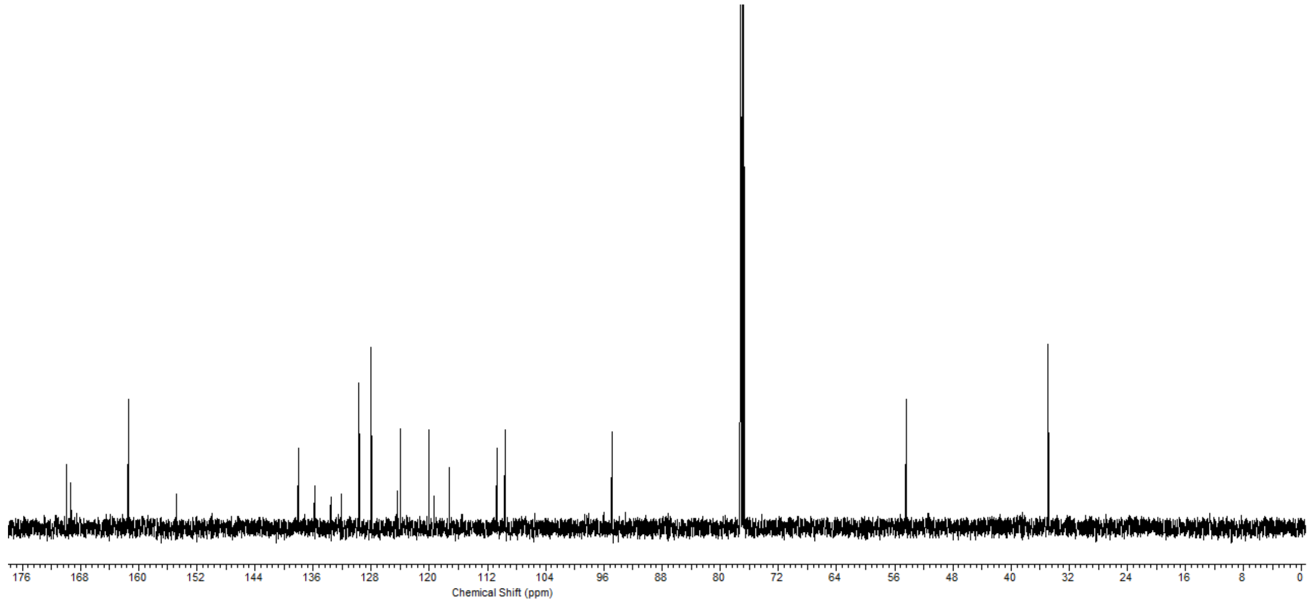
Chemical Shift (ppm)

**Compound 6f**

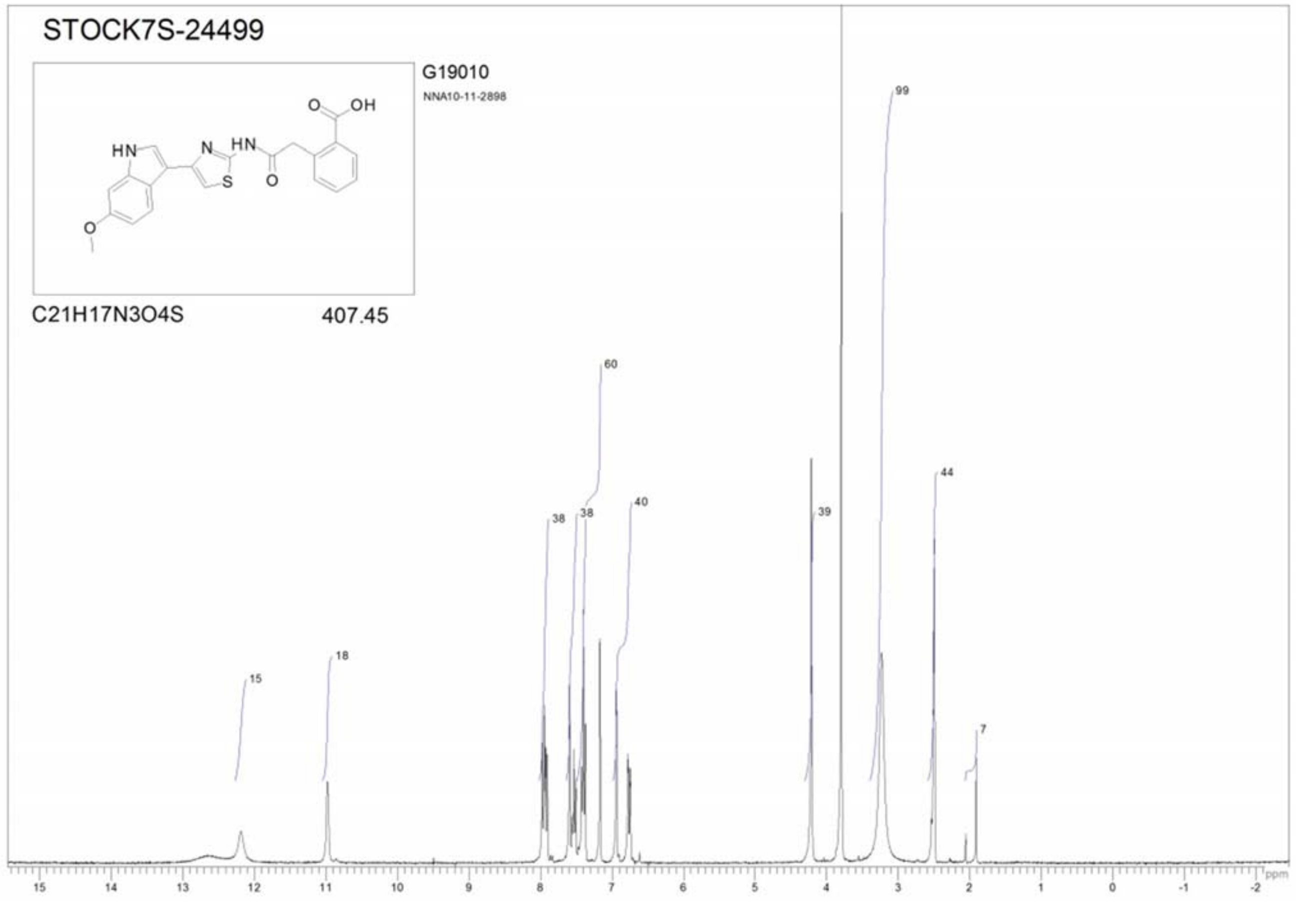


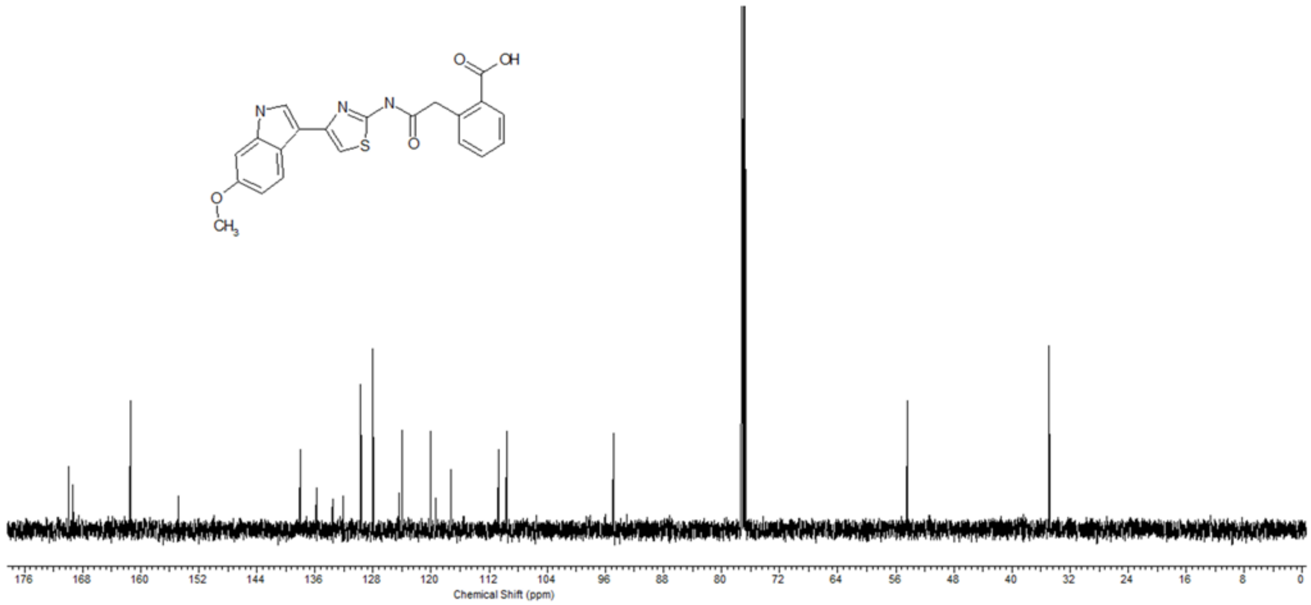


5g OXI H 28480
5l 23917 oxi H

5m 46779 oxi H
6E 24276 OXI C
